# Modelling cervical cancer elimination using single‐visit screening and treatment strategies in the context of high HIV prevalence: estimates for KwaZulu‐Natal, South Africa

**DOI:** 10.1002/jia2.26021

**Published:** 2022-10-12

**Authors:** Darcy White Rao, Cara J. Bayer, Gui Liu, Admire Chikandiwa, Monisha Sharma, Christine L. Hathaway, Nicholas Tan, Nelly Mugo, Ruanne V. Barnabas

**Affiliations:** ^1^ Bill & Melinda Gates Foundation Seattle Washington USA; ^2^ Department of Epidemiology University of North Carolina‐Chapel Hill Chapel Hill North Carolina USA; ^3^ Department of Global Health University of Washington Seattle Washington USA; ^4^ Wits RHI, Faculty of Health Sciences University of the Witwatersrand Johannesburg South Africa; ^5^ Massachusetts General Hospital Boston Massachusetts USA; ^6^ Creighton University School of Medicine Phoenix Arizona USA; ^7^ Kenya Medical Research Institute Nairobi Kenya; ^8^ Harvard Medical School Boston Massachusetts USA

**Keywords:** mathematical model, cervical cancer, HPV infection, HPV vaccine, HIV coinfection, screening

## Abstract

**Introduction:**

In settings with high HIV prevalence, cervical cancer incidence rates are up to six‐fold higher than the global average of 13.1 cases per 100,000 women‐years. To inform strategies for global cervical cancer elimination, we used a dynamic transmission model to evaluate scalable screening and treatment strategies, accounting for HIV‐associated cancer risks and weighing prevention gains against overtreatment.

**Methods:**

We developed a dynamic model of HIV‐HPV co‐infection and disease progression, which we calibrated to KwaZulu‐Natal, South Africa. Our baseline scenario reflects the current practice of HPV vaccination with a multi‐visit screening and treatment strategy involving cytology and colposcopy triage. We evaluated 13 comparator scenarios with increased vaccination coverage and one‐time, two‐time or repeat HIV‐targeted cervical cancer screening with the following single‐visit strategies: HPV DNA testing, HPV genotyping, automated visual evaluation (AVE) and HPV DNA with AVE triage. In all scenarios, HIV antiretroviral therapy, condom use and voluntary male medical circumcision continue at baseline levels. We simulated cancer incidence under each scenario from 2020 to 2120 using the 25 best‐fitting parameter sets. We present the median and range of model output from these simulations to account for parameter uncertainty.

**Results:**

We estimate that cervical cancer incidence will decrease by 87% with the continuation of current cervical cancer and HIV prevention strategies, from an age‐standardized rate per 100,000 women of 80.4 (range 58.2, 112.1) in 2020 to 10.7 (4.2, 29.9) in 2120. Scenarios scaling up vaccination and single‐visit strategies resulted in near‐ and long‐term gains. With repeat HIV‐targeted screening, incidence rates were projected to be 29–34% lower in 2030 relative to the baseline scenario, and elimination (incidence <4/100,000) was achieved with HPV DNA testing in 2095 and with AVE in 2114. A strategy of HPV DNA with AVE triage optimized the tradeoff between cancer cases averted and overtreatment.

**Conclusions:**

Single‐visit screening strategies could avert a substantial burden of cervical cancer and accelerate progress towards elimination in settings with a high burden of HIV. Increasing the screening frequency among women with HIV and reducing loss‐to‐follow‐up for treatment will be key components of a successful elimination strategy.

## 1 INTRODUCTION

Cervical cancer caused over 300,000 preventable deaths in 2018, nearly 90% of which occurred in low‐and‐middle‐income countries (LMICs) [1]. In high‐income countries, effective screening programmes have led to substantial declines in cancer incidence [2]. Vaccination against high‐risk HPV, the etiologic agent causing cervical cancer, has the potential to further decrease cancer incidence [3]. With evidence of the effectiveness of HPV vaccination and screening, the World Health Organization (WHO) Director‐General called for the global elimination of cervical cancer in 2018 [4]. However, delivery and uptake of prevention strategies remains low in many LMICs, due to barriers to healthcare access, insufficient resources [4, 5], and, recently, the COVID‐19 pandemic. Achieving global elimination targets will require a scale‐up of context‐appropriate prevention strategies in LMICs.

In sub‐Saharan Africa, the region with the highest cervical cancer incidence rates [1], high HIV prevalence contributes to the burden of cervical cancer. Women with HIV are at elevated risk of HPV acquisition and progression, and they are more likely to experience treatment failure and recurrence [6], resulting in cancer incidence six‐fold higher than women without HIV [7]. Antiretroviral therapy (ART) mitigates some of these elevated risks, particularly when started early [8], but cancer incidence remains high for women on ART [9]. Analyses accounting for HIV‐HPV synergies are essential to identify optimal strategies for cervical cancer elimination in settings with high HIV prevalence.

In this analysis, we project cervical cancer incidence over the coming century under a range of scenarios using a dynamic model fitted to KwaZulu‐Natal, South Africa, where HIV prevalence among reproductive‐aged women in 2016 was 37% [10]. The standard of care in South Africa is a multi‐visit strategy with cytology and colposcopy triage [11]. However, the proportion of women successfully treated has been low, reflecting a high loss‐to‐follow‐up between visits and shortages of equipment and trained personnel [12]. We compare incidence with this status quo strategy to a scenario with HPV DNA screening, which is recommended as an alternative to cytology [4, 13]. To account for the potential harms of overtreatment [14], we also examine the impact of HPV genotyping, and we model the use of automated visual evaluation (AVE) for both primary screening and triage following a positive HPV diagnosis. AVE is a strategy that uses machine learning to detect lesions from digital cervical images, and early data show great promise for performance and scalability [15]. Our results can inform policy decisions to accelerate cervical cancer elimination in LMICs with high HIV prevalence.

## 2 METHODS

The Data‐driven Recommendations for Interventions against Viral InfEction (DRIVE) model is a compartmental model of HIV and HPV co‐infection and natural history, adapted from a previously published model [16]. It simulates an open population of men and women aged 0–79 years, stratified by sex, 5‐year age group, sexual risk group, and HIV‐ and HPV‐associated health states. HIV health states are stratified by CD4 cell count and viral load. Oncogenic HPV natural history is modelled for two independent genotype groups: types 16/18/31/33/54/52/58 (types covered by the nonavalent [9v] HPV vaccine) and other oncogenic types. The model accounts for increased risks of HPV acquisition, precancer and cancer associated with HIV infection [7, 17], which depend on CD4 cell count and ART status. Further details of the model are provided in the Supplementary Appendix.

HPV and HIV transmission is modelled through sexual contact in heterosexual partnerships. Rates of partnership formation depend on sex, age and sexual risk group, with assortative mixing by age and risk group. The model includes cofactors that modify HIV transmission: ART, voluntary male medical circumcision (VMMC) and condoms. We model the use of ART only for individuals with full HIV viral suppression. Condom use also reduces HPV transmission, and the risk of HPV acquisition increases with declining CD4 count among individuals with untreated HIV.

### 2.1 Model parameterization and calibration

We reviewed the literature to obtain context‐specific estimates for model inputs related to demographic dynamics, sexual behaviour, use of HIV and cancer prevention interventions, and transitions between health states. After identifying parameters for which empirical evidence is limited or uncertain, we used a multi‐phased approach to calibrate our model to historical outcomes. In a preliminary step, we used hand‐calibration to assess the sensitivity of the model to specific inputs and refine prior ranges. We then used an Approximate Bayesian Computation‐Sequential Monte Carlo algorithm [18, 19, 20] in two phases to select parameter sets that provided the best fit to observed targets. Phase 1 sampled from defined ranges for sexual behaviour and HIV natural history and evaluated the fit to demographic and HIV prevalence data. Phase 2 used the 50 best‐fitting parameter sets from phase 1 to fit HPV natural history parameters to empirical targets for HPV prevalence, cervical intraepithelial neoplasia (CIN) prevalence, cervical cancer incidence and HPV genotype distribution. An in‐depth description of the methods and parameter ranges is provided in the Supplementary Appendix Section III.

### 2.2 Modelled scenarios

**Figure 1:**
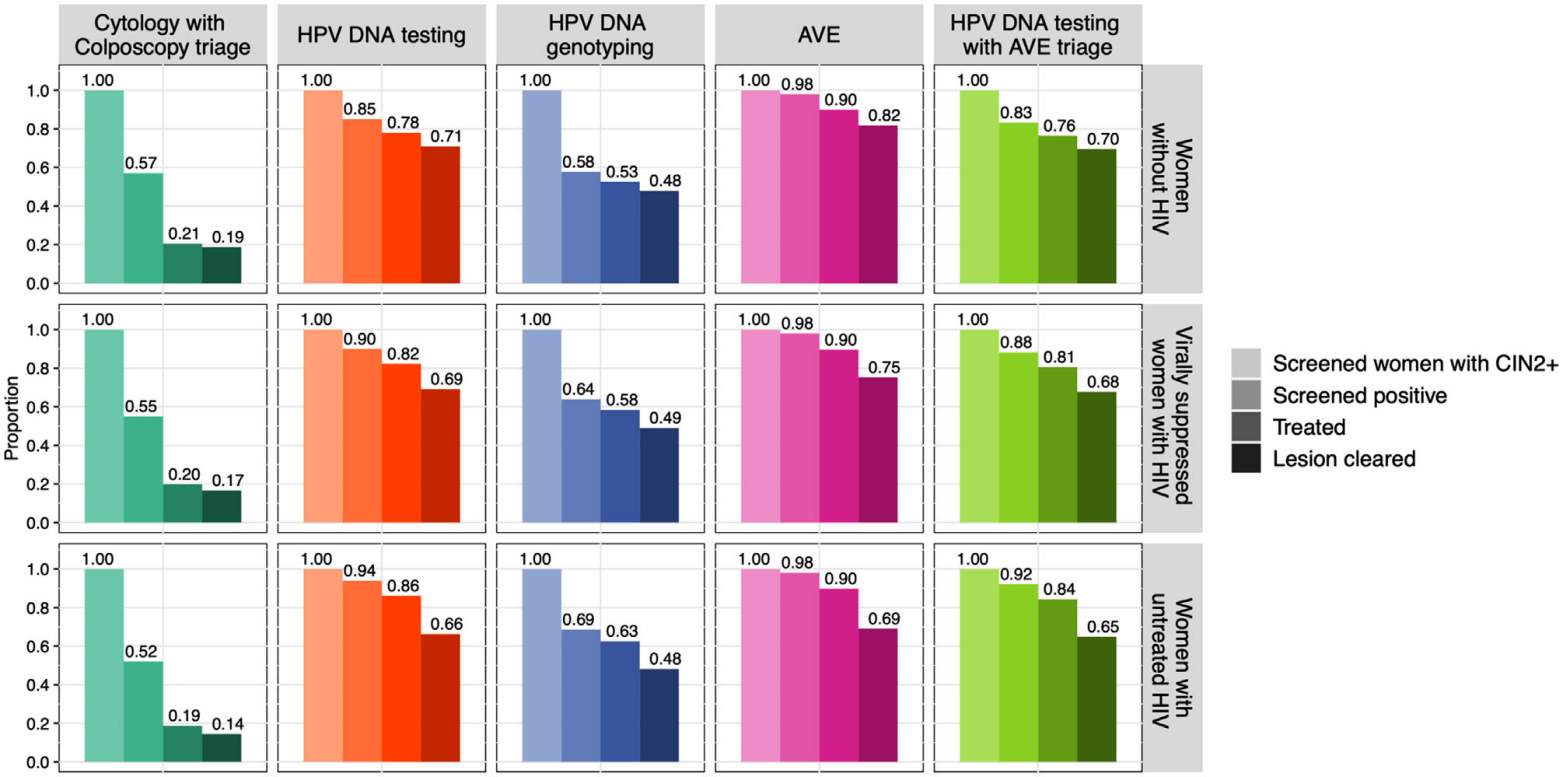
Screening and treatment cascade for the modelled scenarios by HIV status. For each scenario and group, columns depict the proportion of screened women with CIN2+ at the time of screening who screen positive (a function of test sensitivity), are treated (a function of loss to follow‐up) and who are successfully treated. Some women who are successfully treated have persistent HPV and transition to an HPV‐infected (but no CIN) state following treatment, while others return to the HPV susceptible state. For HPV genotyping, sensitivity is derived from the model based on the prevalence of HPV types 16/18/31/33/45/52/58; this varies over time with changes in HPV prevalence and type distribution as HPV vaccination is scaled up. This plot reflects the median sensitivity from model simulations in 2021. Abbreviations: AVE, automated visual evaluation; HPV, human papillomavirus.

We assessed 14 primary scenarios with varying strategies and/or coverage of HPV vaccination, screening and precancer treatment (Table S21). Key differences between the scenarios include test sensitivity, specificity and loss to follow‐up (Figure 1 and Table S22). Our baseline scenario reflects the current standard of care in South Africa: cytology, colposcopy triage and treatment of high‐grade lesions using cryotherapy or large loop excision of the transformation zone (LLETZ) [11]. In this three‐visit scenario, we assume one‐time screening between ages 35 and 39 with 48% coverage [10]. Accounting for observed challenges with loss‐to‐follow‐up and limited availability of equipment and supplies [12, 21], 72% of screen‐positive women are assumed to return for colposcopy and 50% return for treatment [22]. Fifty‐seven percent of girls [23] receive two doses of the bivalent HPV vaccine beginning in 2014, with a switch to the nonavalent vaccine (9vHPV) at ages 9–14 in 2021. In the first comparator scenario, we increase vaccine coverage to 90% in 2021 without changes to screening or treatment.

The next set of scenarios model four single‐visit screening and treatment strategies with 90% 9vHPV coverage beginning in 2021: (1) oncogenic HPV DNA testing; (2) HPV DNA testing with genotyping for HPV types 16, 18, 31, 33, 45, 52 or 58; (3) AVE; and (4) oncogenic HPV DNA testing with AVE triage. In these scenarios, 48% of individuals aged 35–39 are screened as in the baseline scenario, but the performance of the screening strategies differs (Figure 1 and Table S22). Sensitivity is highest with AVE, followed by HPV DNA testing. For HPV‐based strategies, sensitivity is lowest for HIV‐uninfected women and highest for women with untreated HIV, with the reverse pattern for specificity (Table S22). We assume 95% of screen‐positive women eligible for ablation receive immediate treatment with thermocoagulation [24, 25, 26]. Receipt of treatment is lower among women requiring LLETZ [25] (80%).

The final set of scenarios project the impact of repeat screening, including more frequent screening for women with HIV. Using the same four strategies outlined above, we model (1) twice‐lifetime screening for all women at ages 35–39 and 45–49 years, reflecting WHO targets [4], and (2) twice‐lifetime screening for women without HIV and screening every 5 years from ages 25 to 49 years for women with HIV (HIV‐targeted screening). Screening coverage within each age group is held at 48%, and 90% of girls receive 9vHPV vaccination.

In all scenarios, we use published estimates for diagnostic performance and treatment efficacy by HIV status, with estimates reported for women with HIV conservatively applied to those without viral suppression. Because diagnostic and treatment performance are associated with CD4 cell count [27, 28], we assume that estimates for women with virally suppressed HIV fall between estimates for women with detectable HIV and women without HIV. All scenarios hold the proportion of persons with HIV who are virally suppressed and the proportion of men circumcised at levels estimated from the most recent empirical data.

To account for uncertainty in screening parameters, we conducted sensitivity analyses evaluating 14 additional scenarios. The first four isolate the effect of differing performance characteristics of single‐visit strategies by holding retention for treatment, screening frequency and coverage at baseline levels. We additionally evaluate moderate retention for treatment with HIV‐targeted screening using HPV DNA testing (70% retention for thermocoagulation and 50% for LLETZ). A sixth scenario increases screening coverage from 48% to 70% for the strategy of HIV‐targeted HPV DNA testing, aligning with WHO targets [4]. The remaining eight scenarios explore variability in the performance of AVE (Table S23).

### 2.3 Simulation and analyses

Using the 25 best‐fitting parameter sets from model calibration, we simulated HPV and HIV transmission, natural history and demographic dynamics from 2020 (the year before new interventions are introduced) to 2120. Outcomes include cervical cancer incidence rates over time, age‐standardized to the 2015 World Standard Population and the number of cervical cancer cases averted under each scenario relative to baseline. Time to elimination is defined by the years at which estimated cancer incidence crosses the threshold of 4 cases per 100,000 woman‐years (elimination as a public health problem) and a higher threshold of 10 cases per 100,000 [4, 29]. To account for overtreatment, we report the number of women treated for precancer per cancer case averted. Outcomes are summarized as the median and range from the 25 model simulations.

## 3 RESULTS

**Figure 2:**
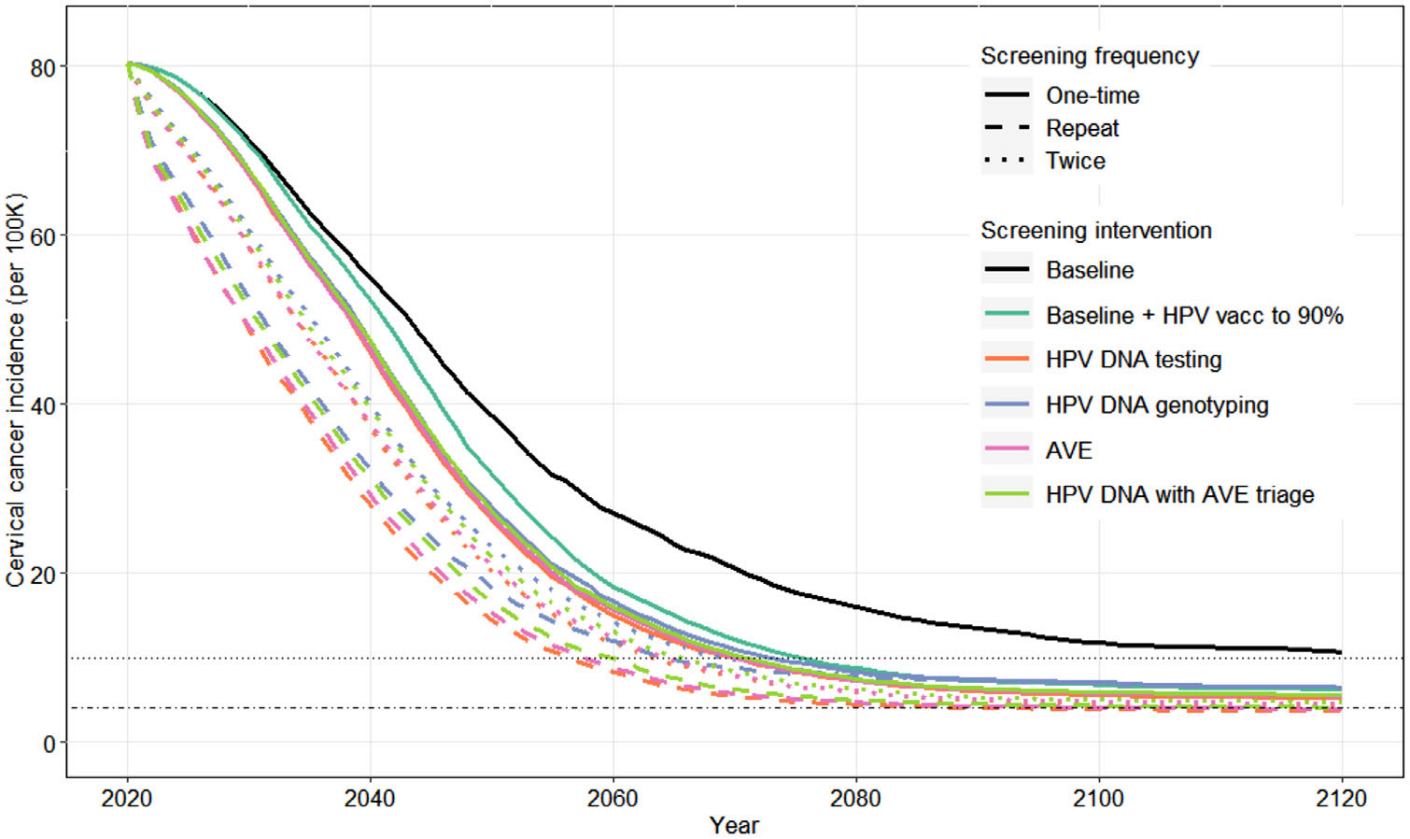
Projected cervical cancer incidence under scenarios that differ in HPV vaccination coverage and cervical cancer screening and treatment frequency, method and loss‐to‐follow‐up. The plotted lines show the median incidence under each scenario across 25 simulations. The colour of the lines defines the screening and vaccination scenario and the line type (solid, dashed or dotted) defines the frequency of screening. In scenarios with one‐time screening, 48% of women are screened between ages 35 and 39. In scenarios with twice‐lifetime screening, screening occurs at ages 35–39 and 45–49, with 48% coverage in each age group. In scenarios with repeat screening, women without HIV are screened at ages 35–39 and 45–49, and women with HIV and screened on average every 5 years from ages 25 to 49 years. In each age group, 48% of eligible women are screened. Horizontal dotted and dot‐dash lines mark the elimination thresholds of 10/100K and 4/100K cases, respectively. Abbreviations: AVE, automated visual evaluation; HPV, human papillomavirus.

The baseline scenario of cytology with colposcopy triage and 57% HPV vaccination resulted in an 87% decrease in cervical cancer incidence from 80.4 cases per 100,000 in 2020 (range 58.2–112.1) to 10.7 cases per 100,000 in 2120 (4.2–29.9; Figure 2). This reflects a 35% (25–52%) relative reduction in HIV prevalence attributable to ART, VMMC and condom use (Figures S27 and S28). Scaling up 9vHPV vaccination to 90% coverage further reduces 2120 cancer incidence to 6.3 cases per 100,000 (3.3–13.6), a 92% reduction from 2020. Owing largely to reduced loss‐to‐follow‐up for treatment (Figure S24 and Table S32), scenarios implementing single‐visit strategies are predicted to accelerate the preventative gains relative to vaccine scale‐up alone but projected 2120 incidence rates are similar to the vaccine scale‐up scenario (Figure 2 and Table 1).

**Table 1:** Projected age‐standardized cervical cancer incidence and time to elimination under modelled scenarios for all women, women living without HIV and women living with HIV

	Age‐standardized^a^ cervical cancer incidence (per 100K) *Median (range)* ^b^			Year elimination threshold reached^c^ *Median (range)* ^b^	
	Year 2030 (after 10 years)	Year 2070 (after 50 years)	Year 2120 (after 100 years)	<10/100K	<4/100K
All women					
*Three‐visit screening and treatment between ages 35 and 39*					
Baseline (cytology with colposcopy triage)	71.3 (50.4, 106.4)	20.5 (10.3, 39.3)	10.7 (4.2, 29.9)	X (2071, X)	X (X, X)
Baseline with vaccine scale‐up^d^	70.9 (50.1, 105.8)	12.1 (6.2, 21.0)	6.3 (3.3, 13.6)	2076 (2060, X)	X (2096, X)
*Single‐visit interventions between ages 35 and 39*					
HPV DNA testing	67.2 (47.5, 98.4)	9.9 (5.1, 16.9)	5.3 (2.8, 11.8)	2070 (2056, X)	X (2081, X)
HPV DNA genotyping	67.9 (48.0, 99.7)	11.0 (6.0, 19.6)	6.5 (3.4, 14.1)	2073 (2058, X)	X (2099, X)
AVE	67.3 (47.6, 98.3)	10.0 (5.2, 17.1)	5.5 (2.9, 11.9)	2070 (2056, X)	X (2082, X)
HPV DNA with AVE triage	67.7 (47.9, 99.3)	10.3 (5.3, 17.6)	5.6 (2.9, 12.2)	2071 (2057, X)	X (2084, X)
*Single‐visit interventions at ages 35–39 and 45–59*					
HPV DNA testing	58.7 (41.9, 84.9)	7.8 (4.1, 13.7)	4.3 (2.4, 10.1)	2064 (2052, X)	X (2072, X)
HPV DNA genotyping	60.8 (43.3, 88.2)	9.8 (5.6, 18.3)	6.5 (3.3, 14.1)	2070 (2055, X)	X (2096, X)
AVE	58.8 (42.1, 840)	7.9 (4.2, 13.8)	4.6 (2.5, 10.2)	2065 (2052, X)	X (2072, X)
HPV DNA with AVE triage	60.1 (42.9, 86.5)	8.4 (4.4, 14.6)	4.8 (2.6, 10.7)	2066 (2053, X)	X (2074, X)
*Single‐visit interventions with HIV‐targeted screening* ^e^					
HPV DNA testing	48.9 (35.2, 69.6)	5.5 (3.0, 10.6)	3.7 (1.8, 8.6)	2057 (2047, 2074)	2095 (2061, X)
HPV DNA genotyping	52.7 (37.5, 75.1)	8.8 (5.2, 17.0)	6.5 (3.3, 14.1)	2065 (2051, X)	X (2093, X)
AVE	49.3 (35.8, 69.7)	5.7 (3.1, 11.0)	4.0 (2.0, 8.9)	2058 (2047, 2077)	2114 (2062, X)
HPV DNA with AVE triage	51.4 (37.0, 72.3)	6.2 (3.4, 11.8)	4.2 (2.1, 9.4)	2060 (2048, 2085)	X (2064, X)
Women without HIV					
*Three‐visit screening and treatment between ages 35 and 39*					
Baseline (cytology with colposcopy triage)	32.8 (15.7, 51.3)	10.9 (4.5, 23.8)	6.3 (2.2, 18.9)	2074 (2046, X)	X (2075, X)
Baseline with vaccine scale‐up^d^	32.4 (15.5, 50.9)	6.1 (2.7, 12.3)	4.1 (1.7, 9.3)	2058 (2042, 2085)	X (2059, X)
*Single‐visit interventions between ages 35 and 39*					
HPV DNA testing	31.1 (14.8, 47.6)	5.1 (2.3, 10.4)	3.5 (1.5, 8.2)	2055 (2040, 2072)	2083 (2056, X)
HPV DNA genotyping	31.4 (14.9, 48.2)	5.7 (2.7, 12.0)	4.3 (1.8, 9.6)	2056 (2040, 2090)	X (2057, X)
AVE	31.0 (14.7, 47.4)	5.2 (2.3, 10.5)	3.6 (1.6, 8.2)	2055 (2040, 2073)	2088 (2056, X)
HPV DNA with AVE triage	31.3 (14.9, 48.1)	5.4 (2.4, 10.8)	3.7 (1.6, 8.4)	2056 (2040, 2074)	2092 (2056, X)
*Single‐visit interventions at ages 35–39 and 45–59*					
HPV DNA testing	27.8 (13.1, 41.7)	4.0 (1.8, 8.5)	3.1 (1.3, 7.0)	2051 (2037, 2064)	2071 (2051, X)
HPV DNA genotyping	28.5 (13.5, 43.1)	5.5 (2.5, 11.3)	4.3 (1.8, 9.6)	2054 (2038, 2087)	X (2055, X)
AVE	27.6 (13.0, 41.0)	4.2 (1.9, 8.5)	3.1 (1.4, 7.1)	2051 (2036, 2064)	2073 (2051, X)
HPV DNA with AVE triage	28.4 (13.4, 42.6)	4.5 (2.0, 9.1)	3.2 (1.4, 7.4)	2052 (2037, 2066)	2075 (2052, X)
*Single‐visit interventions with HIV‐targeted screening* ^e^					
HPV DNA testing	27.5 (13.0, 41.4)	3.7 (1.7, 8.0)	3.0 (1.2, 6.9)	2050 (2036, 2062)	2068 (2050, X)
HPV DNA genotyping	28.2 (13.4, 42.9)	5.3 (2.5, 11.0)	4.3 (1.8, 9.6)	2053 (2037, 2085)	X (2054, X)
AVE	27.4 (12.9, 40.8)	4.0 (1.8, 8.2)	3.1 (1.3, 7.0)	2051 (2036, 2063)	2071 (2050, X)
HPV DNA with AVE triage	28.3 (13.3, 42.4)	4.3 (1.9, 8.7)	3.2 (1.3, 7.3)	2052 (2037, 2065)	2073 (2052, X)
Women with HIV					
*Three‐visit screening and treatment between ages 35 and 39*					
Baseline (cytology with colposcopy triage)	158.1 (116.0, 244.8)	48.1 (24.6, 98.5)	24.3 (10.5, 74.6)	X (X, X)	X (X, X)
Baseline with vaccine scale‐up^d^	157.1 (115.5, 243.8)	29.3 (15.2, 57.1)	14.9 (8.2, 35.4)	X (2092, X)	X (X, X)
*Single‐visit interventions between ages 35 and 39*					
HPV DNA testing	149.9 (110.3, 229.6)	23.5 (12.4, 45.8)	12.8 (7.0, 30.3)	X (2079, X)	X (X, X)
HPV DNA genotyping	151.2 (111.3, 232.1)	26.8 (14.3, 52.7)	15.3 (8.4, 36.7)	X (2094, X)	X (X, X)
AVE	150.4 (110.6, 229.4)	24.5 (12.5, 46.1)	13.2 (7.2, 30.6)	X (2080, X)	X (X, X)
HPV DNA with AVE triage	151.2 (111.2, 231.1)	25.0 (12.8, 47.4)	13.4 (7.3, 31.2)	X (2081, X)	X (X, X)
*Single‐visit interventions at ages 35–39 and 45–59*					
HPV DNA testing	131.9 (97.1, 197.3)	18.3 (10.0, 36.3)	10.4 (5.9, 25.7)	X (2070, X)	X (X, X)
HPV DNA genotyping	135.8 (100.2, 205.1)	23.9 (13.3, 48.2)	15.2 (8.4, 36.6)	X (2093, X)	X (X, X)
AVE	132.2 (96.7, 195.9)	19.3 (10.0, 36.6)	10.8 (6.2, 26.1)	X (2071, X)	X (X, X)
HPV DNA with AVE triage	134.3 (98.6, 200.6)	20.6 (10.5, 38.5)	11.3 (6.4, 27.0)	X (2072, X)	X (X, X)
*Single‐visit interventions with HIV‐targeted screening* ^e^					
HPV DNA testing	108.3 (79.0, 156.1)	11.0 (6.2, 22.5)	7.3 (3.7, 18.0)	2076 (2057, X)	X (2114, X)
HPV DNA genotyping	115.3 (85.2, 170.3)	20.6 (11.9, 42.5)	15.1 (8.3, 36.6)	X (2091, X)	X (X, X)
AVE	109.1 (79.5, 156.7)	12.2 (6.7, 24.0)	8.2 (4.2, 19.2)	2077 (2058, X)	X (X, X)
HPV DNA with AVE triage	113.7 (82.6, 162.7)	12.9 (7.1, 25.9)	8.5 (4.7, 20.3)	2081 (2060, X)	X (X, X)

Abbreviations: AVE, automated visual evaluation; HPV, human papillomavirus; LLETZ, large loop excision of the transformation zone.

^a^ Standardized to the 2015 World Population.

^b^ Median and range of estimates from simulations using the 25 best‐fitting parameter sets.

^c^ X denotes that the elimination threshold was not reached in the simulated time horizon.

^d^ Non‐avalent hrHPV vaccination of girls aged 9–14 scaled up from 57% to 90% coverage. Vaccination coverage remains at 90% for all single‐visit scenarios.

^e^ Ages 35–39 and 45–49 for women living without HIV and every 5 years from 25 to 49 for women living with HIV.

Higher screening frequency is projected to increase near‐term reductions in cervical cancer incidence. Relative to the scenarios with one‐time single‐visit screening, median cervical cancer incidence rates are 12–13% lower in 2030 with twice‐lifetime screening and 23–28% lower with HIV‐targeted screening. Except for the HPV genotyping strategy, which wanes in effectiveness over time, scenarios with repeat screening are also estimated to be more effective over the long term. Primary HPV DNA testing is expected to be the most effective strategy, with incidence rates in 2120 declining to 3.7 per 100,000 (1.8–8.6) with HIV‐targeted screening.

Median incidence crosses below the 10 per 100,000 threshold for all scenarios except the baseline (Table 1). The elimination threshold (4 per 100,000) is only reached with HIV‐targeted screening using HPV DNA testing and AVE, although at least one of the 25 simulations crosses this threshold for all scenarios other than the baseline, and in all scenarios, at least one simulation does not reach this threshold.

Elimination is achieved earlier and with more strategies for women without HIV than for women with HIV (Table 1). Over the 100‐year time horizon, incidence per 100,000 among women without HIV in the baseline scenario decreases from 34.9 (17.7–53.9) in 2020 to 6.3 (2.2–18.9) in 2120. Among women with HIV, the corresponding incidence rates are 178.0 (115.2–257.3) in 2020 and 24.3 (10.5–74.6) in 2120. Median incidence among women with HIV drops below the 10 per 100,000 threshold only with HIV‐targeted screening and does not reach the elimination threshold in any modelled scenario. In sensitivity analyses, increasing screening coverage to 70% with HIV‐targeted HPV DNA testing is predicted to decrease the time to elimination for the total population by approximately 13 years and bring the incidence per 100,000 women with HIV in 2120 to 6.1 (2.2, 14.1; Table S32). With retention for treatment reduced to 70% with thermocoagulation and 50% with LLETZ, the elimination threshold is reached only for women without HIV (Table S32).

**Figure 3:**
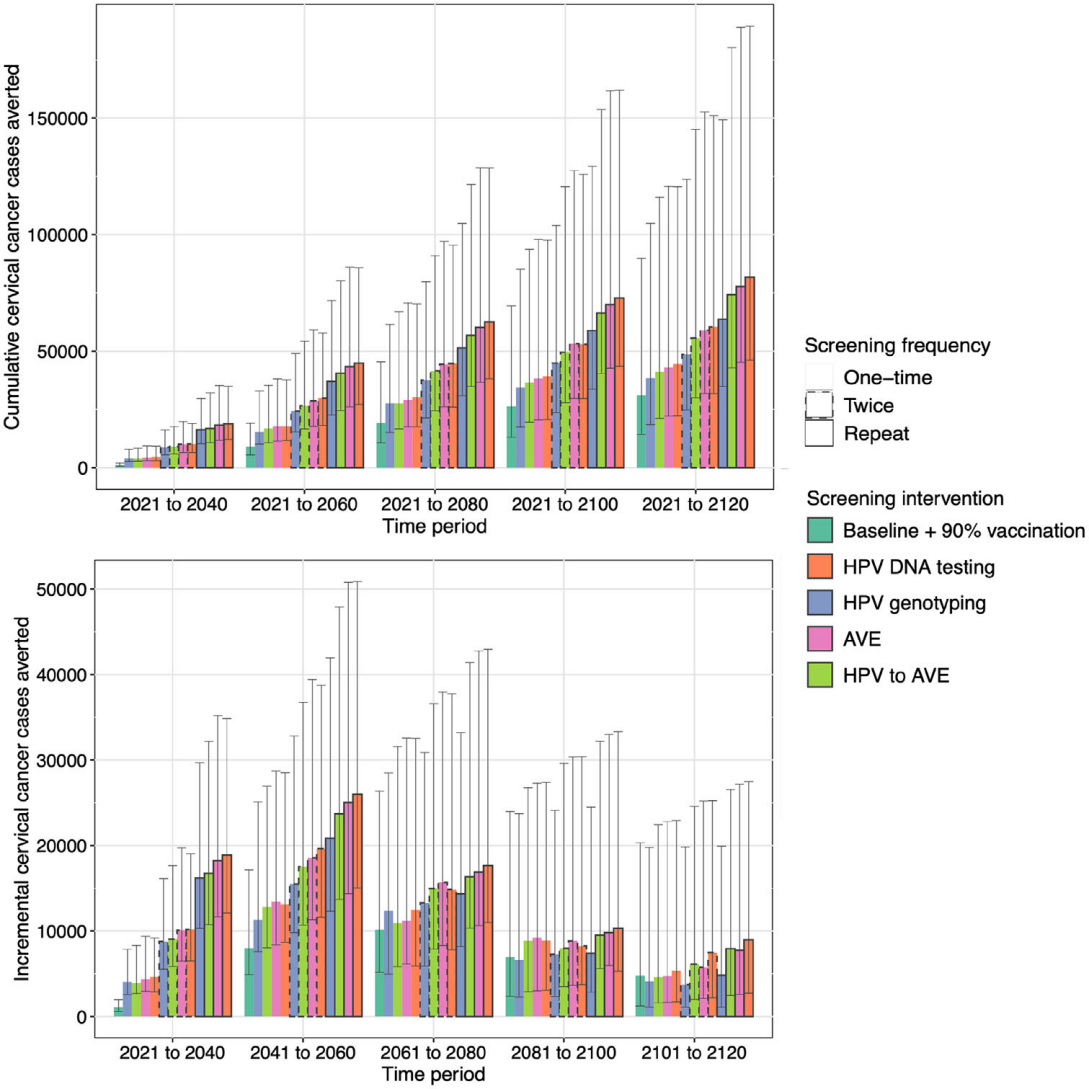
Cumulative (top) and incremental (bottom) cancer cases averted under scenarios that differ in HPV vaccination coverage and cervical cancer screening and treatment frequency, method and loss‐to‐follow‐up. Cases averted are defined with reference to the baseline strategy. The top panel shows the cumulative cases averted over time in 20‐year increments. The bottom panel shows the incremental cases averted over each 20‐year period. The columns show the median estimates from across the 25 simulations and the error bars show the range of estimates. Scenarios are ordered from lowest to highest in terms of cumulative cases averted. Note the different scales on the y‐axis for the two plots. Abbreviations: AVE, automated visual evaluation; HPV, human papillomavirus.

In addition to differences in time to elimination, the scenarios differ in the cumulative number of cancer cases averted (Figure 3). Relative to the baseline scenario, which results in an estimated 152,927 cervical cancer cases over the 100‐year period (89,136–314,863), the percent of cases averted is greatest with HIV‐targeted screening using HPV DNA testing with low loss‐to‐follow‐up (54%; 49–60%), followed by AVE (53%; 48–60%) and HPV DNA with AVE triage (50%; 46–57%). Nearly, a quarter of all cases averted with these strategies are averted within the first 20 years. In comparison, 3% of cases averted with expanded vaccination alone are averted within 20 years.

**Figure 4:**
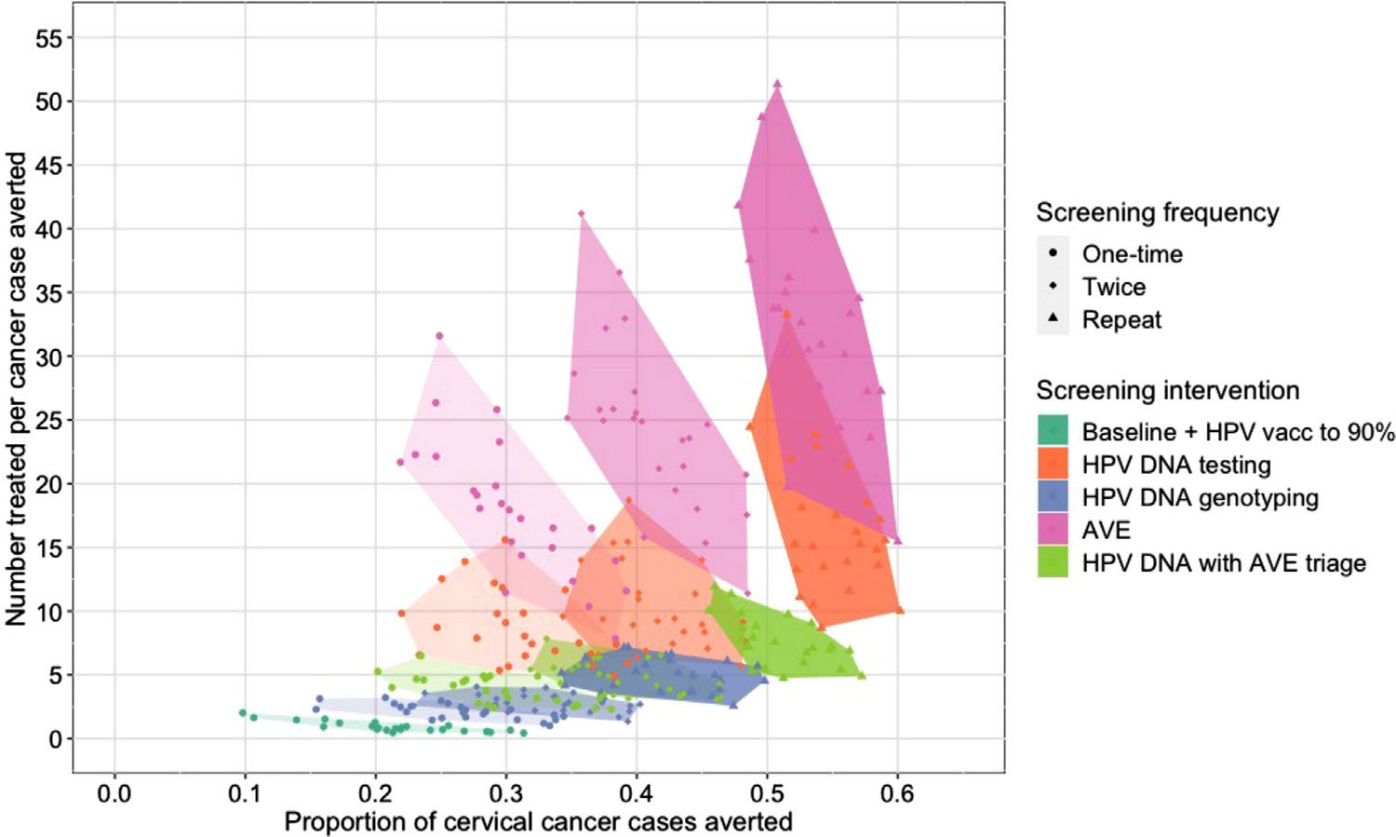
Cumulative proportion of cervical cancer cases averted (x‐axis) by the number of cases treated for precancer per cancer case averted (y‐axis) under scenarios that differ in HPV vaccination coverage and cervical cancer screening and treatment frequency, method and loss‐to‐follow‐up. Cancer cases averted are defined as the difference between the cumulative number of incident cancer cases over the simulated period with the baseline scenario and the cumulative number of cases with each comparator scenario. Here, we show the proportion of cancer cases with the baseline scenario that are averted. The y‐axis shows the ratio of the cumulative number of cases treated with ⩽CIN3 to the number of cancer cases averted. The points show the results from each of the 25 simulations with each scenario. The shaded regions denote the convex hull of the points for each scenario. Lighter shading and round points indicate scenarios with one‐time screening; medium shading and diamond points indicate scenarios with twice‐lifetime screening; darker shading and triangular points indicate scenarios with repeat screening. Abbreviations: AVE, automated visual evaluation; HPV, human papillomavirus.

Figure 4 displays the tradeoff between cancer cases averted and overtreatment. While HIV‐targeted HPV DNA and AVE maximize cases averted, these strategies require 15.3 (8.7, 33.3) and 30.9 (15.5, 51.3) treatments per cancer case averted, respectively. The use of HPV DNA testing with AVE triage is projected to prevent slightly fewer cancer cases but requires only 7.2 treatments per cancer case averted (4.7, 11.9).

In sensitivity analyses lowering the sensitivity and specificity of AVE by up to 20%, projected cancer incidence is similar to the primary analysis, but the efficiency of screening is lower (Figure S26 and Table S32). The number needed to treat to avert one cancer case reaches 58.7 (27.4, 100.0) with AVE as primary screening and 9.1 (5.9, 16.9) with AVE as triage.

## 4 DISCUSSION

Using a dynamic transmission and natural history model that captures HPV‐HIV synergies, we project that cervical cancer elimination is within reach in KwaZulu‐Natal, South Africa. In our baseline scenario reflecting current practice, we estimate that age‐standardized cervical cancer incidence in KwaZulu‐Natal will decline 87% to an expected rate of 10.7 per 100,000 in 2120. This reduction is primarily attributable to the effects of moderate 9vHPV vaccine coverage and HIV prevention interventions. By increasing vaccine coverage to 90%, adopting single‐visit screening and treatment strategies, and providing more frequent services for women with HIV, tens of thousands of cancer cases could be averted and elimination achieved before the end of the century.

Our results are consistent with other models that have simulated the path to elimination. A recent comparative modelling analysis predicted that most countries in sub‐Saharan Africa would reach the threshold of 10 cancer cases per 100,000 with 90% 9vHPV vaccination and no improvements to screening [29]. Scale‐up of once‐ or twice‐lifetime HPV DNA screening was necessary to reach the 4 per 100,000 threshold, particularly in countries starting with an age‐standardized incidence >25 per 100,000. However, the models used in this analysis did not account for HIV–HPV interactions. Using an HIV‐HPV model parameterized to South Africa, van Schalkwyk et al. [21] estimated a reduction in age‐standardized incidence to 12 per 100,000 in 2100 with current practice. Although the scenarios and context are not directly comparable to our model of KwaZulu‐Natal province, the findings are similar in that strategies with higher screening frequency and referral to treatment yield near‐term gains and increase the likelihood of elimination.

A key objective of our analysis was to evaluate scalable strategies. While increasing the coverage and frequency of screening with cytology would also be expected to reduce cancer incidence, limited availability of equipment, supplies and qualified personnel has impeded the effectiveness of the current cytology‐based screening programme [5, 12, 21, 30]. For comparison, we explore a range of strategies that could feasibly be implemented using existing infrastructure and personnel. South Africa has more than 250 GeneXpert machines [11], which can be leveraged for HPV DNA testing and genotyping to provide rapid, accurate results [4, 30]. Although not yet available, work is underway to train algorithms for AVE using smartphone images [31], which could be used in diverse clinical or community‐based settings.

An important determinant of the impact of screening on cervical cancer incidence is the loss‐to‐follow‐up for treatment. HPV testing and AVE can provide rapid results, thereby facilitating same‐visit treatment and increasing retention [30]. The use of HPV testing in screen‐and‐treat programmes has been shown to be feasible and effective for women with and without HIV [30] and is recommended by the WHO [4]. To increase capacity for treatment, we assume a switch from cryotherapy to thermocoagulation, which overcomes some of the challenges with cryotherapy and can be conducted with portable battery‐operated devices [32, 33]. In our baseline status quo scenario, 36% of women with abnormal Pap smears receive colposcopy and treatment [22], higher than the 26% of women who received indicated follow‐up within 18 months of a high‐risk Pap result in a study in South Africa [34]. Lower baseline follow‐up for treatment would increase the expected number of cancer cases averted with single‐visit strategies.

Of note, our primary scenarios assume that screening coverage in each targeted age group is 48%, below the WHO goal of 70% [4]. Screening coverage estimates for South Africa are inconsistent; some suggest the proportion of women screened and/or screening frequency are higher than we have assumed [21, 35]. However, the reductions in cancer incidence that would be expected with higher screening coverage and frequency have not been observed [12, 21], suggesting that uptake is low. Our scenarios examine a critical first step towards improving screening through the adoption of new technologies and management algorithms. With these more scalable single‐visit interventions in place, our sensitivity analysis indicates that efforts to expand coverage would accelerate the timeline to elimination. Increasing coverage will be especially valuable for women with HIV. Given the structure of our model with 5‐year age groups, we approximated the recommended 3‐year screening intervals for women with HIV [13] with 5‐year average intervals. Our results demonstrate the impact of HIV‐targeted screening and support efforts to improve fidelity to guidelines.

An important tradeoff to screening and treating more women is the treatment of individuals whose infections may clear spontaneously. Cervical treatment may increase the risk of adverse reproductive outcomes [14], and treatment of women without precancer wastes valuable healthcare resources. We found that overtreatment was highest with AVE‐and‐treat, followed by HPV DNA testing. Using HPV genotyping and AVE to triage HPV‐positive results helped mitigate overtreatment with relatively modest reductions in cancer cases averted. Several novel biomarkers, including the E6 oncoprotein and viral methylation [36], also have the potential to improve screening specificity. With more empirical data on these new methods, cost‐effectiveness and budget impact analyses will be valuable to identify efficient and affordable strategies.

Strengths of our analysis include the use of a dynamic model that represents HIV transmission and disease progression and the associations with the risk of HPV acquisition and natural history. Our model is calibrated to available data on demographics, HIV and HPV epidemiology using a multi‐phased Bayesian approach. We defined scenarios for simulation based on the review of the literature and consultation with South African collaborators to reflect feasible changes to the screening and treatment programme. Additionally, our model differs from other models assessing cervical cancer elimination by estimating overtreatment to compare alternative strategies more comprehensively.

Our analysis has several limitations. Although we validated our model to external targets for HIV prevalence and incidence, we did not have an independent data set for the validation of cervical cancer outcomes. To account for uncertainty in model parameters, including sexual behaviour and the natural history of infection, we utilized a Bayesian calibration procedure to fit our model to an extensive set of empirical infection and disease targets. This reduced the precision of our estimates but provided a more complete portrayal of the range of likely outcomes. Another source of uncertainty is in our assumptions about future behaviours and technologies. Sexual and care‐seeking behaviours will likely change over time, limiting the accuracy of long‐term predictions. However, this limitation affects all models simulating future health events. Importantly, our conclusions regarding the relative impact of single‐visit strategies are consistent, if not stronger, in analyses restricting the time horizon to 10–20 years.

Limited data were available on the clinical performance of AVE, as it has not yet been widely implemented [15, 37]. We explored a range of assumptions for sensitivity and specificity, with results suggesting that AVE may be an effective strategy to triage HPV DNA results even with up to 20% lower performance than reported by Hu et al. [15]. As this technology is further tested, updated model‐based analyses and economic evaluations will be warranted. For HPV‐based strategies, we derived estimates of specificity from the simulations as the proportion of all screened women ⩽CIN1 who screen negative. With this approach, the specificity of HPV DNA testing (Table S22) is higher than published estimates [38, 39, 40]. A potential explanation for this discrepancy is that our model may simulate a lower prevalence of HPV‐infected states ⩽CIN1 than among sampled populations, leading it to underestimate overtreatment with HPV‐based strategies. The modelled specificity of the HPV genotyping strategy is also higher and the sensitivity lower than observed in a study that evaluated testing for HPV types 16/18/31/33/35/45/52/58 [39]. Because our model groups HPV35 with other oncogenic types not included in the 9vHPV vaccine, we modified the strategy to target only the other seven types. In practice, HPV35 would likely be included in genotyping algorithms, thereby increasing the sensitivity of this strategy.

With our compartmental model structure, we were not able to simulate differences in screening intervals or surveillance based on individual screening history [13, 41]. Future analyses with individual‐based models would be valuable to refine recommendations. Additionally, we made the simplifying assumption that all women with HIV would be similarly likely to undergo screening. Data suggest that untreated women may be screened at rates similar to women without HIV [21], in which case, our model would overestimate the impact of screening for women with HIV. However, our scenarios make conservative assumptions regarding screening coverage and frequency, which may balance this effect. We also did not model ART discontinuation, which might shift the distribution of cancer and mortality risks among women with HIV. However, we fit our model to the proportion of people with HIV who are virally suppressed, thus representing effective ART coverage.

## 5 CONCLUSIONS

Our model provides strong evidence that cervical cancer elimination is achievable in the coming century in settings with a high dual burden of HIV and cervical cancer. The need for multiple visits combined with shortages of supplies and equipment has limited the effectiveness of screening programmes in many LMICs to date. The scenarios we evaluated leverage effective, scalable single‐visit strategies that have the potential to increase access to screening and reduce barriers to treatment. Our results highlight the importance of more frequent screening for women with HIV, suggesting that integration of HIV and cervical cancer services could help to decrease disparities and accelerate reductions in incidence.

## COMPETING INTERESTS

NM receives research funding from Merck Ltd. RVB declares support from the US National Institutes of Health and the Bill and Melinda Gates Foundation, and funding from Regeneron Pharmaceuticals for manuscript writing and abstract submission outside the submitted work. CJB receives payment from Merck, although this relationship began after the submission of the manuscript. The other authors have no conflicts to declare.

## AUTHORS’ CONTRIBUTIONS

DWR, CJB, GL, MS, NT and RVB developed the model, with programming and simulations for the present analysis done by CJB and CLH. The study was conceptualized by DWR, CJB, GL and RVB. All authors contributed to scenario definition and analysis planning. DWR wrote the first draft of the manuscript. All authors contributed to the interpretation of the findings, provided critical review and revisions, and approved the final version.

## FUNDING

This work was supported by the National Center for Advancing Translational Sciences under Award Number KL2 TR002317, and funding from the National Cancer Institute (U01 CA199334) and the World Health Organization. Partial support for this research came from a Eunice Kennedy Shriver National Institute of Child Health and Human Development grant, P2C HD042828, to the Center for Studies in Demography & Ecology at the University of Washington.

## DISCLAIMER

The content is solely the responsibility of the authors and does not necessarily represent the official views of the US National Institutes of Health.

## Supporting information


**Supplementary Appendix**: Model inputs and methods.

## Data Availability

Model inputs and methods are presented in the Supplementary Appendix. Model code is available upon request with submission of a concept note to the authors.
