# Supplementary material for: Modelling cervical cancer elimination using single‐visit screening and treatment strategies in the context of high HIV prevalence: estimates for KwaZulu‐Natal, South Africa
Source: J Int AIDS Soc. 2022 Oct 12;25(10):e26021. doi: 10.1002/jia2.26021 (PMC9557021; doi:10.1002/jia2.26021)
Supplement: Supplementary file 1 — Supplementary Appendix: Model inputs and methods. [file JIA2-25-e26021-s001.docx]

**Supplemental Appendix**

Supplement to: Rao DW, Bayer CJ, Liu G, Chikandiwa A, Sharma M, Hathaway CL, Tan N, Mugo N, Barnabas RV. Modeling cervical cancer elimination using single-visit screening and treatment strategies in the context of high HIV prevalence: estimates for KwaZulu-Natal, South Africa

Table of Contents

[I. Model overview 4](#_Toc107601305)

[II. Modules and parameter values 4](#_Toc107601306)

[II.a. Demography 4](#_Toc107601307)

[II.b. Sexual behavior 6](#_Toc107601308)

[II.c. Natural history 8](#_Toc107601309)

[II.c.i. HIV 8](#_Toc107601310)

[II.c.ii. HPV 10](#_Toc107601311)

[II.d. Transmission probabilities 14](#_Toc107601312)

[II.e. Interventions to prevent and treat HIV and HPV/cervical cancer 15](#_Toc107601313)

[II.e.i. HIV antiretroviral therapy (ART) 15](#_Toc107601314)

[II.e.ii. Condoms 17](#_Toc107601315)

[II.e.iii. Circumcision 17](#_Toc107601316)

[II.e.iv. Historical HPV vaccination and cervical cancer screening and treatment (pre-2021) 19](#_Toc107601317)

[II.f. Future scenarios for cervical cancer screening, treatment, and HPV vaccination 21](#_Toc107601318)

[III. Calibration and validation 26](#_Toc107601319)

[III.a. Calibration 26](#_Toc107601320)

[III.a.i. Phase 0 26](#_Toc107601321)

[III.a.ii. Bayesian calibration approach 26](#_Toc107601322)

[III.a.iii. Phase 1 26](#_Toc107601323)

[III.a.iv. Phase 2 35](#_Toc107601324)

[III.a.v. Approximate Bayesian Computation-Sequential Monte Carlo (ABC-SMC) algorithm 45](#_Toc107601325)

[III.a.vi. Likelihood calculation 46](#_Toc107601326)

[III.b. Validation 46](#_Toc107601327)

[IV. Differential equations 55](#_Toc107601328)

[IV.a. Demography 57](#_Toc107601329)

[Fertility 57](#_Toc107601330)

[Aging 58](#_Toc107601331)

[Mortality 58](#_Toc107601332)

[IV.b. Sexual Behavior 58](#_Toc107601333)

[Mixing matrix 58](#_Toc107601334)

[Rate of partner change 59](#_Toc107601335)

[IV.c. Transmission Probabilities 60](#_Toc107601336)

[Per-partnership probability of transmission 60](#_Toc107601337)

[Force of infection 61](#_Toc107601338)

[IV.d. Natural History and Interventions 61](#_Toc107601339)

[HIV 61](#_Toc107601340)

[HPV 63](#_Toc107601341)

[Vaccine-type HPV and precancer equations 64](#_Toc107601342)

[Non-vaccine-type HPV and precancer equations 65](#_Toc107601343)

[Cervical cancer equations 65](#_Toc107601344)

[V. Additional Model Output 66](#_Toc107601345)

[V.a. Sensitivity analyses 66](#_Toc107601346)

[Isolating the impact of differences in screening test performance 66](#_Toc107601347)

[Moderate loss to follow-up 67](#_Toc107601348)

[Screening coverage increased to 70% 67](#_Toc107601349)

[Variable AVE screening and diagnostic performance 68](#_Toc107601350)

[V.b. Cervical cancer incidence under additional counterfactual scenarios 72](#_Toc107601351)

[V.c. Projected HIV prevalence 73](#_Toc107601352)

[VI. References 74](#_Toc107601353)

# I. Model overview

The *DRIVE (Data-driven Recommendations for Interventions against Viral infEction)* model is a deterministic, compartmental model parameterized to represent transmission of human papillomavirus (HPV) and progression to cervical cancer in KwaZulu-Natal, South Africa (KZN), a region with high HIV prevalence.^1,2^ The model and the accompanying technical appendix were adapted from a previously-published model fit to this context.^3^ The primary objectives of the model are to evaluate and predict the impact of HPV vaccination, cervical cancer screening and treatment, and HIV prevention and treatment on cervical cancer outcomes. Beginning in 1925, the model generates a population stratified by age, gender and sexual risk group. This population engages in heterosexual transmission of oncogenic HPV (high-risk HPV; hrHPV) and, beginning in 1980, HIV infection.

HIV progression is tracked by both CD4+ T-cell (CD4) count and HIV RNA concentration (viral load), and infected individuals may achieve viral suppression with initiation of antiretroviral therapy (ART), beginning in 2004. Men and women infected with hrHPV may clear the infection, and infections in women may progress through stages of precancerous lesions to cervical cancer. A key feature of our model is representation of the interaction between HIV and HPV, whereby HIV infection increases the risk of HPV acquisition and the rate of disease progression. The model is calibrated to reproduce observed population-level HPV and HIV dynamics using maximum-likelihood estimation.

Model dynamics are governed by a system of differential equations that are solved in MATLAB using a 4^th^-order Runge-Kutta numerical method. HPV is introduced in 1925 to allow HPV transmission dynamics and cervical cancer incidence to equilibrate prior to the introduction of HIV infection in 1980. The model simulates events in discrete time with two-month intervals. At each time step, differential equations are evaluated to estimate population demographics and the number of persons in each infection, disease, or treatment state for the following time step. The dynamic nature of our transmission model captures population-level effects such as herd immunity.

This work was facilitated through the use of advanced computational, storage, and networking infrastructure provided by the Hyak supercomputer system at the University of Washington.

# II. Modules and parameter values

## II.a. Demography

The model represents a dynamic, open population of men and women aged 0 to 79 in KwaZulu-Natal. To allow HPV transmission dynamics to equilibrate prior to the introduction of HIV in 1980, the model is initialized in 1925. Because data on population demographics are not available prior to 1950, we projected backwards from more recent estimates. The United Nations Population Division estimates the South African population by gender and age from 1950 to 2020, and Statistics South Africa calculates the proportion of the population residing in KZN in the years 2002 to 2019.^4,5^ KZN’s share of the total population decreased linearly during this period, and we assumed the trend was slightly steeper between 1925 and 2002 to provide an initial population size large enough to absorb HIV-associated deaths and match observed data for KZN in 2002. In this way, we derived the KZN population in 1950 by gender and age from national data, and then fit exponential distributions for each gender and age group to project backwards to 1925 (Table S1).

At each two-month time step, we calculate the number of births, natural deaths, and persons aging to the next five-year group. Total fertility rates determine how many male and female newborns enter the population over time. We model a 56% linear decline in fertility from 1960 to 2000, followed by a ten-year stabilization until 2010, and then a second 24% linear decrease from 2010 values by 2020 consistent with literature, United Nations Population Division total fertility estimates, and KZN population estimates.^4-7^ These rates are applied to women aged 15-49, stratified by age and by CD4 cell count in women with HIV^8,9^ (Table S2). The proportion of births from mothers with HIV that result in perinatal infection decreases linearly from 34% in 2004 to 29.2% by 2005, and to 7.1% by 2008, capturing improvements in services for pregnant women living with HIV^10-12^ (Table S3). We assume a 1:1 gender ratio at birth and that infected newborns are born into the acute stage of HIV.

To age the population, one-fifth of each compartment moves to the next age group annually. Upon aging to the next five-year group, individuals are re-distributed into the closest unfilled risk group to match observed data on the age distribution of low, moderate, and high-risk individuals (defined by annual number of sexual partnerships; see section on Sexual Behavior). All compartments, except for the youngest 0 to 4 age group, thus experience inflows from the prior age group and outflows into the next age group.

Persons leave the population due to death or aging past age 79. To model deaths, we apply South African age- and gender-specific mortality rates (Table S4). Before 1950, we use United Nations 2019 Population Prospects abridged life tables from the period of 1950 to 1955.^4^ We then assume that background mortality decreases linearly to 1985 Population Prospects values. After the start of the generalized HIV epidemic in 1980, we account for both background and HIV-specific mortality. To estimate background mortality rates, we subtract HIV-specific mortality from all-cause mortality estimates from the IHME Global Burden of Disease Study,^13^ and we assume linear changes in background mortality between 1985 and 2000 and between 2000 and 2020.

For future simulations, we model a 50% linear decline in fertility rates from 2020 to 2035 to match projected United Nations Population Division estimates for population size, age distribution, and total fertility.^4,5^

**Table S1. Initial population size by age and gender.** The population distribution by gender and 5-year age groups was backward projected from observed data on the total South Africa population and proportion residing in KZN assuming exponential growth between 1925 and 1950.^4,5^

| Age group | Initial population size | |
| --- | --- | --- |
|  | **Men** | **Women** |
| 0 – 4 | 232193 | 234484 |
| 5 – 9 | 159981 | 163174 |
| 10 – 14 | 130604 | 133156 |
| 15 – 19 | 116664 | 114322 |
| 20 – 24 | 105385 | 99000 |
| 25 – 29 | 95487 | 87233 |
| 30 – 39 | 88296 | 80039 |
| 35 – 39 | 81236 | 71998 |
| 40 – 44 | 73180 | 65881 |
| 45 – 49 | 62270 | 56376 |
| 50 – 54 | 49095 | 47787 |
| 55 – 59 | 38061 | 38751 |
| 60 – 64 | 27479 | 32112 |
| 65 – 69 | 18576 | 23810 |
| 70 – 74 | 12335 | 15545 |
| 75 – 79 | 6595 | 7570 |
| TOTAL | 1297437 | 1271238 |

**Table S2. Baseline fertility rates by age and HIV status.** Fertility rates before 1960.^8,9^ Linear decreases in 1960 and 2010 are applied to this matrix. Women on ART are assumed to have equal fertility to women without HIV.

| Age group | Annual fertility rates before 1960 | | | | | |
| --- | --- | --- | --- | --- | --- | --- |
|  | **HIV-uninfected or women with HIV on ART** | **Acute** | **CD4 >500** | **CD4 350-500** | **CD4 200-350** | **CD4 <200** |
| 0 – 14 | 0 | 0 | 0 | 0 | 0 | 0 |
| 15 – 19 | 0.1555 | 0.1555 | 0.1555 | 0.0902 | 0.0902 | 0.0638 |
| 20 – 24 | 0.3058 | 0.3058 | 0.3058 | 0.1774 | 0.1774 | 0.1254 |
| 25 – 29 | 0.3120 | 0.3120 | 0.3120 | 0.1809 | 0.1809 | 0.1279 |
| 30 – 34 | 0.2323 | 0.2323 | 0.2323 | 0.1347 | 0.1347 | 0.0953 |
| 35 – 39 | 0.1483 | 0.1483 | 0.1483 | 0.0860 | 0.0860 | 0.0608 |
| 40 – 44 | 0.0596 | 0.0596 | 0.0596 | 0.0346 | 0.0346 | 0.0244 |
| 45 – 49 | 0.0194 | 0.0194 | 0.0194 | 0.0112 | 0.0112 | 0.0079 |
| 50 – 79 | 0 | 0 | 0 | 0 | 0 | 0 |

**Table S3. Proportion of births from women living with HIV that result in mother-to-child transmission.** The mother to child transmission (MTCT) rate decreases linearly from 2004 to 2005 and from 2005 to 2008.^10-12^

| Year | Proportion of births with MTCT |
| --- | --- |
| Before 2004 | 0.340 |
| By 2005 | 0.292 |
| After 2008 | 0.071 |

**Table S4. Background mortality rates by age and gender.** The population follows the background mortality rate of South Africa. Before 1950, we use United Nations data from the period of 1950 to 1955.^4^ We then assume that background mortality decreases linearly until 1985, the start of the generalized HIV epidemic. Background mortality changes linearly between 1985 and 2000 and between 2000 and 2020 according to estimates from the IHME Global Burden of Disease Study.^13^

|  | **Annual background mortality rates** | | | | | | | |
| --- | --- | --- | --- | --- | --- | --- | --- | --- |
| **Age group** | **Before 1950** | | **By 1985** | | **By 2000** | | **By 2020** | |
|  | **Men** | **Women** | **Men** | **Women** | **Men** | **Women** | **Men** | **Women** |
| **0 – 4** | 0.1901 | 0.1628 | 0.0570 | 0.0474 | 0.0144 | 0.0118 | 0.0054 | 0.0046 |
| **5 – 9** | 0.0087 | 0.0074 | 0.0025 | 0.0019 | 0.0013 | 0.0010 | 0.0004 | 0.0003 |
| **10 – 14** | 0.0048 | 0.0044 | 0.0016 | 0.0012 | 0.0010 | 0.0008 | 0.0002 | 0.0001 |
| **15 – 19** | 0.0060 | 0.0049 | 0.0025 | 0.0017 | 0.0022 | 0.0018 | 0.0007 | 0.0005 |
| **20 – 24** | 0.0085 | 0.0056 | 0.0038 | 0.0024 | 0.0044 | 0.0050 | 0.0021 | 0.0010 |
| **25 – 29** | 0.0094 | 0.0065 | 0.00438 | 0.0031 | 0.0079 | 0.0087 | 0.0030 | 0.0018 |
| **30 – 34** | 0.0105 | 0.0073 | 0.0052 | 0.0038 | 0.0110 | 0.0093 | 0.0040 | 0.0026 |
| **35 – 39** | 0.0122 | 0.0083 | 0.0064 | 0.0046 | 0.0131 | 0.0095 | 0.0049 | 0.0030 |
| **40 – 44** | 0.0144 | 0.0096 | 0.0080 | 0.0057 | 0.0141 | 0.0086 | 0.0055 | 0.0033 |
| **45 – 49** | 0.0166 | 0.0105 | 0.0101 | 0.0067 | 0.0172 | 0.0094 | 0.0074 | 0.0045 |
| **50 – 54** | 0.0206 | 0.0136 | 0.0140 | 0.0090 | 0.0227 | 0.0120 | 0.0108 | 0.0059 |
| **55 – 59** | 0.0255 | 0.0180 | 0.0186 | 0.0120 | 0.0282 | 0.0153 | 0.0161 | 0.0086 |
| **60 - 64** | 0.0352 | 0.0270 | 0.0278 | 0.0185 | 0.0378 | 0.0218 | 0.0230 | 0.0117 |
| **65- 69** | 0.0506 | 0.0420 | 0.0428 | 0.0304 | 0.0466 | 0.0303 | 0.0321 | 0.0165 |
| **70 - 74** | 0.0771 | 0.0674 | 0.0685 | 0.0517 | 0.0680 | 0.0452 | 0.0405 | 0.0231 |
| **75 - 79** | 0.1190 | 0.1070 | 0.1120 | 0.0881 | 0.0839 | 0.0559 | 0.0570 | 0.0353 |

## II.b. Sexual behavior

In our model, sexual activity begins in the 10-14 age group. In each sexually active age group, the population is divided into three risk groups with variable rates of partnership formation (Table S5). The distribution of these risk groups by age was derived from Africa Centre cohort partnership data,^14^ calculated as the percentage of the cohort in age groups 15-19 through 45-49 who reported 0-1 recent partners (low risk), 2-4 recent partners (moderate risk), or 5 or more recent partners (high risk). In age groups for which no respondents reported 5 or more recent partners, we borrowed information from neighboring age groups to estimate the proportion we would expect to be high-risk given that the sample size for some age groups was small. Individuals aged 10-14 are assumed to be predominately low-risk, and the risk distribution for ages 45-49 is extrapolated up to age 79. The risk distribution derived from male partner data is used for both men and women for simplicity. However, as described below, the rates of partner change and coital frequency within risk and age groups are calibrated and allowed to vary by gender.

Risk groups are distinguished by the number of sexual partners each person is expected to have per year. While data are available on the distribution of partnership counts from the Africa Centre cohort data,^14^ we calibrate the yearly partner change rates for each risk group and gender using prior ranges informed by these data (Table S24). In addition to uncertainty in reported estimates due to reporting biases, this approach is intended to partially account for the effects of concurrency on increasing transmission. While this does not fully capture the effects of concurrency on transmission dynamics, our compartmental model structure is not equipped to represent concurrent partnerships;^15^ using partnership numbers as reported would underestimate the rate of infection spread. However, we define bounds on our priors to keep partnership counts within a plausible range. These parameters are defined such that higher risk groups have fewer acts per partnership per year, reflecting an assumption of shorter partnership duration in higher risk groups. We calibrate the distribution of acts per partnership by age among women (Table S24). Among men, we assume those aged 10-19 have the same number of acts per partnership as women, whereas men aged 20-79 have equal acts to women of the next lowest age group, reflecting age disparities in relationships.

Using methods similar to other models,^16^ we define mixing matrices to describe patterns of sexual contact by age and sexual-risk groups. Informed by data on age discrepancies in KZN,^17,18^ we define an age mixing matrix with partnerships being most likely to form between women and men of the next oldest 5-year age group (see section IV.b.). To account for uncertainty in this distribution, we calibrate a mixing parameter ($\epsilon_{\alpha}$; Table S24), which determines the degree to which the distribution of partnerships by male and female age group deviates from this off-diagonal matrix. The parameter $\epsilon_{\alpha}$ can range from 0, which corresponds to no change from this off-diagonal matrix, to 1 (random mixing), in which case mixing is proportional to the relative sizes of all compartments. For mixing by risk group, we apply a parameter of $\epsilon_{r}=0.3$ (Table S6).^16^ In this case, a value of $\epsilon_{r}=0$ would correspond to an identity matrix with purely assortative (like-with-like) risk group mixing, whereas $\epsilon_{r}=1$ corresponds to random mixing.

Because our model assumes purely heterosexual contact, the modeled number of partnerships expected for men must be consistent with the number expected for women. The observed data used to inform these parameters are subject to selection and response biases that may result in imbalances, and our calibration procedure may also result in imbalanced parameters. We thus adjust contact rates such that the number of partnerships among men in a given age and risk group equals the number of partnerships that women have with men of that same age and risk group. We assume that this adjusted contact rate is equally driven by estimated rates for men and women due to lack of data to assume otherwise.

We assume that sexual behavior will remain unchanged in future simulations.

**Table S5. Sexual risk distribution by age.** Risk distribution is derived from partner data from the Africa Centre cohort (now on AHRI) for KwaZulu Natal, South Africa.^14^ The population aged 15-49 was divided in groups defined as low-risk (0-1 recent partners), moderate-risk (2-4 recent partners), or high risk (5+ recent partners). Children 10-14 are assumed to be predominantly low-risk, and the risk distribution for ages 45-49 is extrapolated up to age 79. The risk distribution from male partner data is used for both men and women in the model.

| **Age group** | **Risk distribution** | | |
| --- | --- | --- | --- |
|  | **Low-risk** | **Moderate-risk** | **High-risk** |
| **10 – 14** | 0.980 | 0.015 | 0.005 |
| **15 – 19** | 0.509 | 0.408 | 0.083 |
| **20 – 24** | 0.472 | 0.443 | 0.085 |
| **25 – 29** | 0.510 | 0.412 | 0.078 |
| **30 – 34** | 0.605 | 0.342 | 0.054 |
| **35 – 39** | 0.766 | 0.203 | 0.031 |
| **40 – 44** | 0.818 | 0.168 | 0.014 |
| **45 – 49** | 0.851 | 0.148 | 0.001 |
| **50 – 54** | 0.851 | 0.148 | 0.001 |
| **55 – 59** | 0.851 | 0.148 | 0.001 |
| **60 - 64** | 0.851 | 0.148 | 0.001 |
| **65- 69** | 0.851 | 0.148 | 0.001 |
| **70 - 74** | 0.851 | 0.148 | 0.001 |
| **75 - 79** | 0.851 | 0.148 | 0.001 |

**Table S6. Sexual mixing by sexual risk group (*ϵ_r_*).** ^16^

| Mixing by sexual risk group |
| --- |
| 0.3 |

## II.c. Natural history

### II.c.i. HIV

HIV is introduced to the model in 1980 with an initial prevalence of 2% across all population subgroups. HIV infection occurs either through heterosexual transmission or mother-to-child transmission. The natural history of HIV infection is then modeled in stages defined by CD4 count and viral load as shown in Figure S1. When a person becomes HIV-infected, s/he enters the acute stage characterized by a short duration and high probability of onward HIV transmission. The person then progresses through stages of decreasing CD4 count. Viral load decreases from the acute to asymptomatic phases and then increases. Transition rates are based on literature describing the average duration in each stage by gender and age.^19-22^ HIV-associated mortality rates with untreated infection are estimated from studies of untreated persons living with HIV and depend on CD4 cell count and age.^23-25^ The combinations of disease progression and mortality rates imply that untreated women have a longer average expected survival than untreated men, and persons aged 5-49 live longer with untreated infection than young children and older adults. Children under age four have the highest HIV-specific mortality, and adults >50 years are assumed to experience HIV mortality rates two times that of persons aged 5-49.^26,27^

**Figure S1. HIV CD4 and viral load state transitions and durations for women (top) and men (bottom).** Natural history of HIV infection. Values for CD4 count in the acute phase and for viral load in each phase of infection are approximated to capture the trajectory of disease over time. The implications of each CD4 and viral load state for mortality, HIV transmission, and susceptibility to HPV and cervical cancer are described in below sections of the appendix.

**Table S7. The duration of time in each CD4 stage by gender and age.^20,22^** These average durations determine the rate at which individuals transition between states. The actual time spent in each state is slightly lower on average due to the effects of background and disease-specific mortality rates.

| Age group | Duration of time spent in CD4 stage with untreated infection (years) | | | | |
| --- | --- | --- | --- | --- | --- |
|  | **Acute** | **CD4 ≥500** | **CD4 350-500** | **CD4 200-350** | **CD4 ≤200** |
|  | **Men** | | | | |
| 0 – 4 | 0.25 | 0.25 | 3.56 | 4.67 | 2.13 |
| 5 – 49 | 0.25 | 0.25 | 3.56 | 4.67 | 3.70 |
| 50 – 79 | 0.25 | 0.25 | 2.85 | 4.51 | 1.85 |
|  | **Women** | | | | |
| 0 – 4 | 0.25 | 0.93 | 3.71 | 4.68 | 2.13 |
| 5 – 49 | 0.25 | 0.93 | 3.71 | 4.68 | 3.70 |
| 50 – 79 | 0.25 | 0.29 | 3.34 | 4.23 | 1.85 |

**Table S8. The duration of time in each viral load stage by gender and age.^19,21^** We define time in the asymptomatic stage such that the total time spent in all viral load stages matches the total time spent in CD4 stages. This is based on data indicating that, while there is variability in time to AIDS, the trajectory in the pre-AIDS and AIDS phases are similar across groups.^19^

| Age group | Duration of time spent in viral load stage (years) | | | | |
| --- | --- | --- | --- | --- | --- |
|  | **Acute** | **Asymptomatic** | **Pre-AIDS symptomatic** | **AIDS** | **Late-stage** |
|  | **Men** | | | | |
| 0 – 4 | 0.25 | 5.03 | 4.00 | 0.75 | 0.83 |
| 5 – 49 | 0.25 | 6.60 | 4.00 | 0.75 | 0.83 |
| 50 – 79 | 0.25 | 3.88 | 4.00 | 0.75 | 0.83 |
|  | **Women** | | | | |
| 0 – 4 | 0.25 | 5.87 | 4.00 | 0.75 | 0.83 |
| 5 – 49 | 0.25 | 7.44 | 4.00 | 0.75 | 0.83 |
| 50 – 79 | 0.25 | 4.13 | 4.00 | 0.75 | 0.83 |

**Table S9. HIV-associated mortality rates by age and CD4 count.** HIV-associated mortality is estimated from observational studies of untreated persons with HIV and depends on CD4 cell count and age.^23-25^ Mortality from observed studies reflects both background and HIV-specific mortality, so we derived HIV-specific mortality by subtracting the average background mortality rate among men and women aged 30-34 (approximately the median age for the included studies) from the estimated rates. Children under age four have the highest disease mortality and adults >50 years have rates two times that of persons aged 5-49.^26,27^

| Age group | Annual HIV-associated mortality rates | | | | |
| --- | --- | --- | --- | --- | --- |
|  | **Acute** | **CD4 ≥500** | **CD4 350-500** | **CD4 200-350** | **CD4 ≤200** |
| 0 – 4 | 0 | 0.4700 | 0.4700 | 0.4700 | 0.4700 |
| 5 – 49 | 0 | 0.0035 | 0.0255 | 0.0455 | 0.2655 |
| 50 – 79 | 0 | 0.0071 | 0.0511 | 0.0911 | 0.5311 |

### II.c.ii. HPV

The model begins simulation in 1925 with an HPV prevalence of 20% among 15-44-year-old men and women. A 55-year burn-in period allows HPV transmission and cervical cancer incidence dynamics to equilibrate prior to the introduction of HIV in 1980. Because we are interested in cervical cancer outcomes, our model represents infection only with oncogenic HPV types (high-risk HPV; hrHPV). Infections are grouped as either nonavalent vaccine-type hrHPV (types 16, 18, 31, 33, 45, 52, and 58) or non-vaccine type hrHPV (all other oncogenic types).

Oncogenic HPV in women can progress to precancerous lesions (categorized as cervical intraepithelial neoplasia (CIN) grades 1, 2, or 3) and cervical cancer (categorized as local, regional, or distant) as shown in Figure S2. We assume that HPV infection persists throughout all stages of CIN and cervical cancer disease. CIN1,2,3 can regress and HPV infection can clear naturally. All women who clear HPV develop partial natural immunity against reinfection with the same HPV type group (nonavalent vaccine-type or non-vaccine type) that wanes over time (Table S13). Due to uncertainty in empirical data, we calibrate the strength of natural immunity protection among women (Table S26) and acquisition, clearance, progression, and regression rates for each stage of infection and each infection type group (vaccine type or non-vaccine type; Table S27). In men, we model infection with the same two groups of HPV types, with clearance resulting in a transition back to the susceptible state – we assume that men who clear HPV do not develop natural immunity.^28^ However, men clear HPV faster than women,^29^ at a rate that is calibrated in the model to reproduce observed HPV prevalence data (Table S26).

Persons living with HIV who have progressed past the acute stage of HIV experience higher rates of HPV acquisition, immunity waning, and disease progression, and lower rates of HPV clearance and disease regression relative to HIV-negative individuals.^30^ CD4 cell count is inversely correlated with the development of cervical cancer. Risk multipliers for HPV acquisition, clearance, and immunity waning among women with HIV relative to HIV-negative women are based on previous modeling,^3^ while the risk multipliers for CIN progression or regression in women with HIV are more uncertain, and therefore calibrated (Table S29). Cervical cancer-associated mortality rates in women with HIV were modified from rates in women without HIV^31^ to account for increased mortality with decreasing CD4 cell count^32^. In our model, the effect of HIV infection on mortality decreases as cervical cancer progresses.^32^


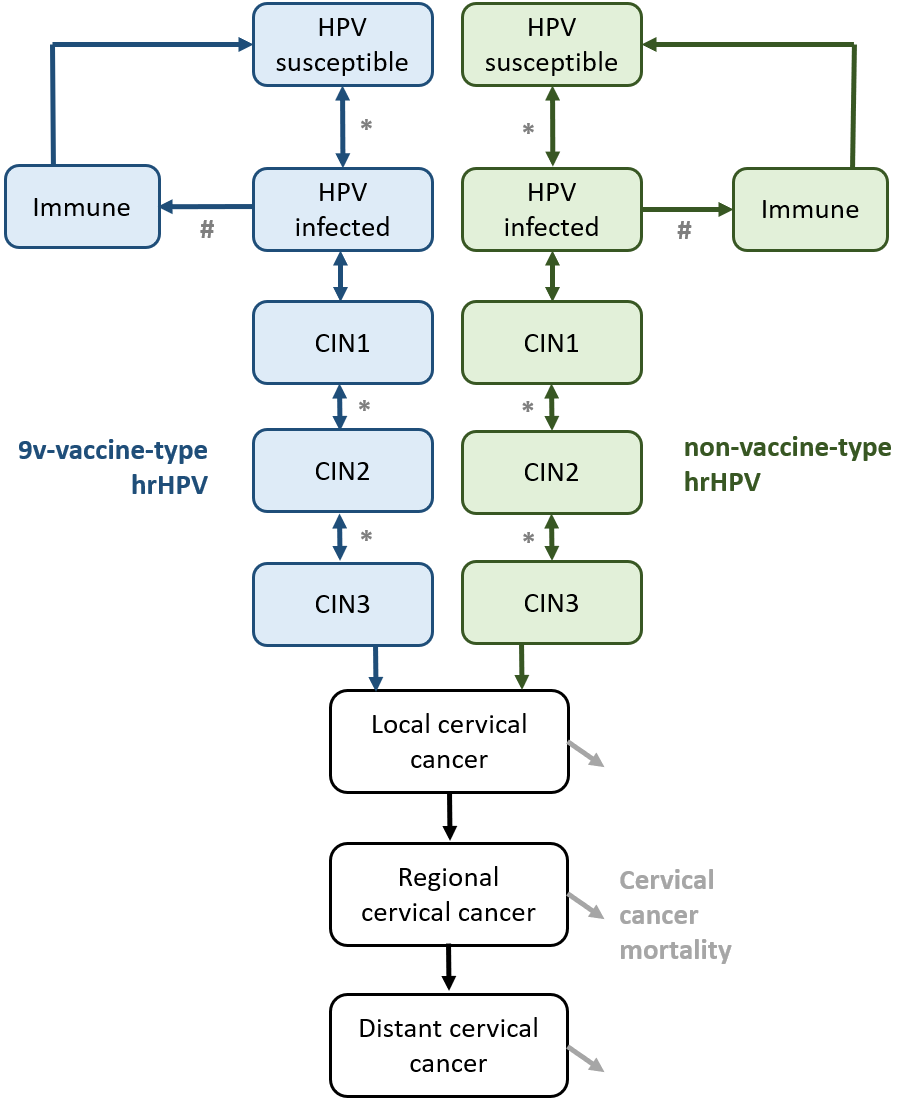


**Figure S2. HPV state transitions and effect modification with concurrent HIV infection.** A diagram of the natural history of HPV infection in relation to HIV infection dynamics. Individuals can be infected with 9v-vaccine-type and non-vaccine-type hrHPV simultaneously.

*HPV state transitions depend on HIV status, CD4 count, and receipt of ART. #Only women develop natural immunity.

**Table S10.** **Cervical cancer-associated mortality by cancer disease stage and CD4 count among women living with HIV.** ^31,32^

|  | **Annual cervical cancer-associated mortality rates** | | |
| --- | --- | --- | --- |
|  | **Local** | **Regional** | **Distant** |
| **HIV uninfected** | 0.0624 | 0.1502 | 0.5207 |
| **HIV-positive on ART** | 0.2071 | 0.2418 | 0.5207 |
| **CD4 ≥500** | 0.2071 | 0.2418 | 0.5207 |
| **CD4 350-500** | 0.2515 | 0.2936 | 0.5207 |
| **CD4 250-350** | 0.3052 | 0.3563 | 0.5207 |
| **CD4 ≤250** | 0.4489 | 0.5240 | 0.5207 |

**Table S11. Cervical cancer progression rates.^33^**

| Monthly cervical cancer progression rates | |
| --- | --- |
| Local to Regional | 0.020 |
| Regional to Distant | 0.025 |

**Table S12.** **HPV type distribution.** This type distribution was used to guide calibration of progression, regression, and clearance parameters for nonavalent vaccine-type and non-vaccine-type infections. We estimated the distribution from published studies reporting on the proportion of cases at different stages of disease testing positive for specific HPV types.^34-41^ The type distribution for CIN2 is estimated as the average of the distributions for CIN1 and CIN3.

|  | Oncogenic HPV type distribution by stage of infection | | | | |
| --- | --- | --- | --- | --- | --- |
|  | **HPV** | **CIN1** | **CIN2** | **CIN3** | **Cervical Cancer** |
| 9v-type HPV | 0.4682 | 0.5192 | 0.6281 | 0.7371 | 0.8578 |
| Non-9v-type HPV | 0.5318 | 0.4808 | 0.3719 | 0.2629 | 0.1422 |

**Table S13. Annual rate of natural immunity waning.^42^**

| Annual rate of waning from immune to susceptible |
| --- |
| 0.024 |

**Table S14.** **HPV incidence multipliers for persons living with HIV by CD4 count and treatment status.** Estimates were calibrated to the KwaZulu-Natal setting, as described in a previous publication^3^. As described below, persons on ART are assumed to be virally suppressed.

| HPV acquisition multipliers for persons living with HIV | |
| --- | --- |
| On ART | 1.00 |
| CD4 ≥500 | 1.78 |
| CD4 350-500 | 1.99 |
| CD4 200-350 | 2.12 |
| CD4 ≤200 | 2.32 |

**Table S15.** **HPV clearance multipliers for persons living with HIV by CD4 count and treatment status.** These multipliers modify the clearance rates from HPV infected to uninfected among men living with HIV and from HPV infected to immune among women living with HIV. Estimates were calibrated to the KwaZulu-Natal setting, as described in a previous publication.^3^ As described below, persons on ART are assumed to be virally suppressed.

| HPV clearance multipliers for persons living with HIV | |
| --- | --- |
| On ART | 0.60 |
| CD4 ≥500 | 0.60 |
| CD4 350-500 | 0.55 |
| CD4 200-350 | 0.45 |
| CD4 ≤200 | 0.30 |

**Table S16.** **Multipliers for the rate of natural immunity waning among persons living with HIV by CD4 count and for treatment status**. Estimates were calibrated to the KwaZulu-Natal setting, as described in a previous publication.^3^ As described below, persons on ART are assumed to be virally suppressed.

| Natural immunity waning multipliers for persons living with HIV | |
| --- | --- |
| On ART | 1.42 |
| CD4 ≥500 | 1.42 |
| CD4 350-500 | 1.57 |
| CD4 200-350 | 1.97 |
| CD4 ≤200 | 2.83 |

## II.d. Transmission probabilities

The number of people who acquire HIV or HPV infection at each time step is determined by gender, age, and risk-group specific forces of infection. These rates are a function of the rate of partner change, the prevalence of infection, patterns of sexual mixing, and per-partnership transmission probabilities. Per-partnership transmission probabilities reflect the cumulative risk of acquiring HIV or HPV from all sexual acts assumed to occur within a partnership (per-partnership transmission = $1-\left( 1-p \right)^{a}$, where $a$ is the number of acts and $p$ is the probability of transmission per act). The number of acts per partnership depends on the age and risk group of the HIV- or HPV-negative partner and is highest for young adults and those in lower risk groups, reflecting assumptions of higher coital frequency for young adults and that partnerships among low-risk individuals are of longer duration.

For HIV transmission, the probability of transmission per sex act depends on the viral load of the partner with HIV. The risk of HIV transmission is highest during the initial acute stage of infection. Risk decreases during the asymptomatic phase of HIV infection before gradually rising as individuals progress to pre-AIDS symptomatic and AIDS stages.^19,43-45^ We calibrate HIV transmission per act in the asymptomatic stage and apply risk multipliers from literature across the other stages of infection (Tables S17, S24). We assume the probability of male-to-female HIV transmission is equal to the probability of female-to male transmission across all viral load stages. For HPV, the probability of transmission per act is assumed to be equivalent for vaccine-type and non-vaccine-type hrHPV and across all stages of pre-cancer and cervical cancer. Due to a lack of robust empirical data, we calibrate this value (Table S26).

As a proxy for decreased sexual activity due to pain and sickness during late-stage HIV, we reduce HIV per-act transmission to 10% of the AIDS rate and, similarly, reduce HPV per-act transmission by 90%.^45^ We assume women with regional or distant cervical cancer (FIGO stages 2-4) also decrease their sexual activity due to pain, reducing HIV and HPV transmission by 50%.

**Table S17. Risk multipliers for HIV transmission by viral load.** The probability of HIV transmission per act depends on the viral load of the partner living with HIV.^19,43-46^

| Risk multiplier for HIV transmission | | | | |
| --- | --- | --- | --- | --- |
| Acute | **Asymptomatic** | **Pre-AIDS symptomatic** | **AIDS** | **Late-stage** |
| 9.0 | 1.0 | 2.5 | 7.0 | 0.7 |

## II.e. Interventions to prevent and treat HIV and HPV/cervical cancer


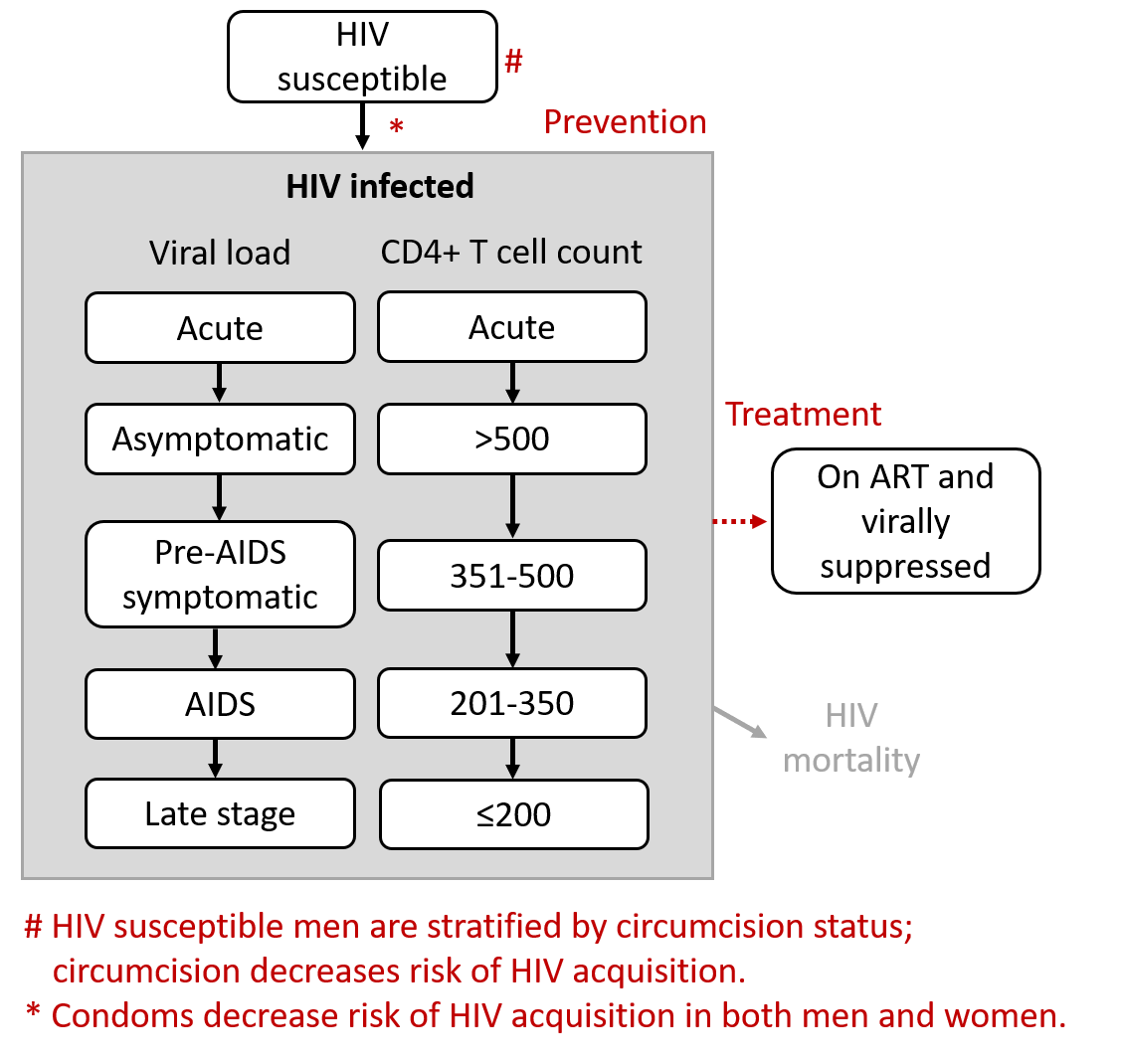


**Figure S3. Effects of HIV-focused interventions on HIV natural history.**

### II.e.i. HIV antiretroviral therapy (ART)

Beginning in 2004, individuals may achieve viral suppression with initiation of ART. We only model the effect of ART among individuals who achieve viral suppression: persons with HIV who initiate treatment without achieving viral suppression are assumed to have no benefit from treatment and are not tracked in our model. Persons on treatment with viral suppression are assumed to have zero probability of transmitting HIV,^47,48^ and to have the same fertility rates as HIV-negative women. HIV-associated mortality is reduced among treated persons, and it decreases over time as the average CD4 count at ART initiation increases,^49-52^ as described below in this section. Persons who are treated with viral suppression are assumed to experience rates of HPV acquisition comparable to individuals without HIV, but HPV clearance, disease progression rates, disease regression rates, and cervical cancer-associated mortality are equivalent to untreated individuals with high (>500) CD4 count.

We define ART coverage as the percentage of all persons living with HIV and age-eligible for ART who are on treatment and virally suppressed. We assume individuals aged 10-14 or older are eligible for ART. The scale-up of ART over time depends on CD4 count and gender. Reflecting changes in policy for treatment eligibility in South Africa,^53^ ART becomes available in 2004 to persons with a CD4 count ≤200 cells/µL. The initiation threshold is subsequently raised to CD4 ≤350 cells/µL in 2011 and to CD4 ≤500 cells/µL in 2015. In 2016, ART becomes available to all individuals living with HIV regardless of their CD4 cell count, including those with acute infection. Informed by empirical data on ART coverage and viral suppression over time in South Africa and KwaZulu Natal specifically,^2,54-57^ we model higher rates of viral suppression in women than men. The proportion of men living with HIV who are treated with viral suppression increases from 0% in 2004 to approximately 44% in 2017, whereas the proportion of women who achieve viral suppression reaches 60% in 2017 (the latest year for which data were available) (Table S18).^2,54-57^

Among individuals of the same gender and CD4 cell count, the probability of ART initiation is uniform by age or risk group. However, we do not model the process of discontinuation of ART and resulting loss of viral suppression, such that the cumulative probability of being on ART increases with age. This results in proportions with viral suppression that are too low in younger ages and too high in older ages compared to observed data. To control this age differential, we apply a minimum bound of 0.85*(target population-level viral suppression) and a maximum bound of 1.04*(target population-level viral suppression) within each age group; eligible individuals are initiated on ART with viral suppression if coverage falls below the minimum for a given age group, and we discontinue treatment for some individuals if coverage exceeds the maximum within an age group. These age-specific minimum and maximum limits ensure that viral suppression is distributed more appropriately in all age groups while also matching the population-level levels of viral suppression by gender in the observed data.

Trends in HIV-associated mortality among treated persons living with HIV mirror changes to the ART initiation threshold to reflect higher baseline health among persons initiating treatment over time. HIV-associated mortality with treatment is defined relative to background mortality, informed by empirical data,^49-52^ and is applied additively. From 2004 to 2011, HIV-associated excess mortality among virally suppressed persons living with HIV is 0.5x the background rate (i.e., the mortality rate from background causes and HIV combined among persons with viral suppression is 1.5 times the background mortality rate). This multiplier decreases to 0.4x, 0.25x, and 0.15x the background mortality rate in 2011, 2015, and 2016, respectively.

In our simulations projecting forward in time, we assume no future scale-up of ART. The proportion of persons living with HIV who are virally suppressed remains at the estimated levels for 2017 for the duration of the simulation.

**Table S18. Proportion of persons living with HIV on ART and virally suppressed in KZN.** We derived estimates of viral suppression from observed data from 2005-2011 and 2017.^2,54-57^ Between these coverage targets, we assume a linear increase in viral suppression.

| Proportion virally suppressed over time by gender | | |
| --- | --- | --- |
| Year | **Men** | **Women** |
| 2004 | 0 | 0 |
| 2005 | 0.0057 | 0.0077 |
| 2006 | 0.0218 | 0.0294 |
| 2007 | 0.0476 | 0.0643 |
| 2008 | 0.0820 | 0.1108 |
| 2009 | 0.1152 | 0.1557 |
| 2010 | 0.1416 | 0.1913 |
| 2011 | 0.1760 | 0.2378 |
| 2017 | 0.4421 | 0.5975 |

**Table S19. HIV-associated excess mortality with viral suppression.^49-52^**

| Time period | **Multiplier on background mortality** |
| --- | --- |
| 2004 to 2011 | 0.5 |
| 2011 to 2015 | 0.4 |
| 2015 to 2016 | 0.25 |
| After 2016 | 0.15 |

**
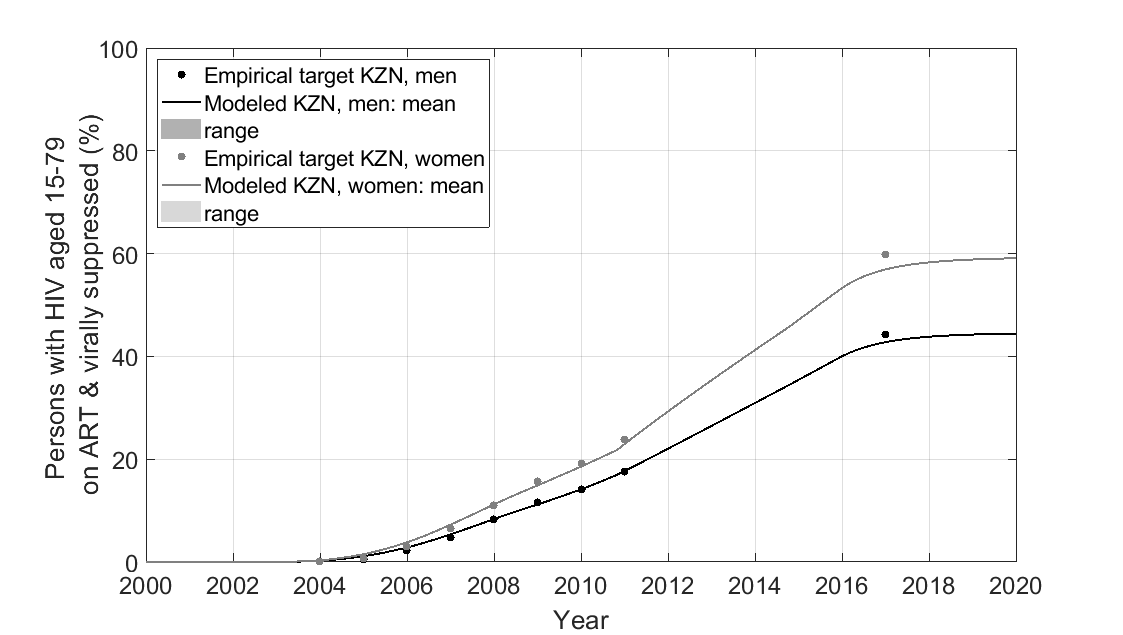
**

**Figure S4. Proportion of persons living with HIV on ART and virally suppressed over time.** Shaded regions represent the range of estimates using the 25 best-fitting model parameter sets, although given that the proportion with viral suppression was a model input, there is little variability in this outcome.

### II.e.ii. Condoms

Condom use is defined as the average proportion of the population ever using condoms multiplied by the percent of sexual acts for which those persons use a condom. Condom use is initiated in the model in 1995, scales up linearly until 2000, and then remains constant. We calibrate the level of condom use in 2000 (see Table S26 in section III.a.iii). We assume condoms reduce HIV acquisition in both men and women by 80%.^58^ Condoms reduce HPV acquisition among women by 70% and HPV acquisition among men by 46%^59,60^. Condoms provide better coverage of the vaginal canal and cervix than the lower penile shaft and scrotum, providing a plausible mechanism for differential HPV protection by gender.

### II.e.iii. Circumcision

We assume that men without HIV who receive voluntary medical male circumcision (VMMC) have decreased risk of HIV. Data suggest that men circumcised by a medical professional have a 60% lower risk of acquiring HIV.^61,62^ Although there is evidence that VMMC also reduces the risk of acquiring HPV for men without HIV,^63,64^ other studies have reported more equivocal findings.^65-68^ Our model does not account for this effect. As a result, our model may overestimate HPV transmission; however, VMMC is held constant across scenarios, such that we do not expect this to meaningfully change our conclusions.

We model medical circumcision beginning in 1960 for age groups 15-19 and 20-24. Prior to the initiation of the South Africa National VMMC program in 2010, circumcision was primarily targeted to young adult men as a rite of passage.^69^ In addition to accounting for the historical practice of circumcision,^70,71^ starting circumcision in 1960 among youth results in circumcision prevalence among men aged 50 and older in 2012 corresponding to observed estimates.^72^ We assume coverage increases linearly from 1960 to 2000 and between 2000 and 2008 to match coverage levels estimated from SABSSM data.^69,73^ Following initiation of the national VMMC program in 2010, we model scale-up of circumcision for all men aged 15 or older at levels extrapolated backwards from 2012-2017 SABSSM and DHS data.^1,2,72^ We assume country-level VMMC coverage by age is reflective of that in KwaZulu-Natal, as the overall proportion of men who received VMMC is similar in SA and KZN,^2,72^ and province-level estimates are not provided by age.

In our future scenarios, the proportion of men circumcised remains at 2017 levels for the duration of the simulations.

**Table S20. Proportion of HIV-negative men who receive VMMC.^1,2,69,72,73^** *Prior to 2010, the coverage of circumcision in men aged 25 and older slowly increases as men circumcised between ages 15-24 age into these groups. We begin tracking this in the model in 2010 to ensure that coverage matches observed targets, with additional men newly circumcised in these older ages if necessary.

| VMMC coverage among HIV-negative men over time by age group | | | | |
| --- | --- | --- | --- | --- |
|  | **Age group** | | | |
| Year | **15-19** | **20-24** | **25-49** | **50-79** |
| 1960 | 0.040 | 0.060 | * | * |
| 2000 | 0.100 | 0.130 | * | * |
| 2008 | 0.114 | 0.161 | * | * |
| 2010 | 0.143 | 0.201 | 0.140 | 0.120 |
| 2012 | 0.172 | 0.242 | 0.191 | 0.143 |
| 2017 | 0.459 | 0.420 | 0.318 | 0.204 |

**
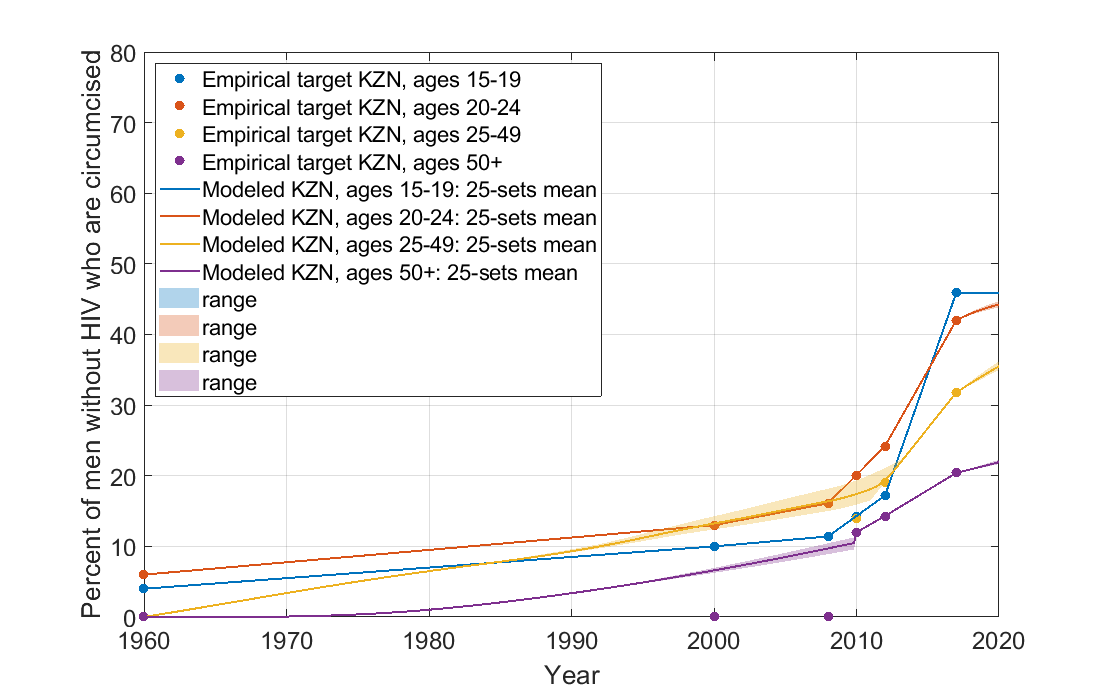
**

**Figure S5. Percentage of men without HIV circumcised over time.** Shaded regions represent the range of estimates using the 25 best-fitting model parameter sets.


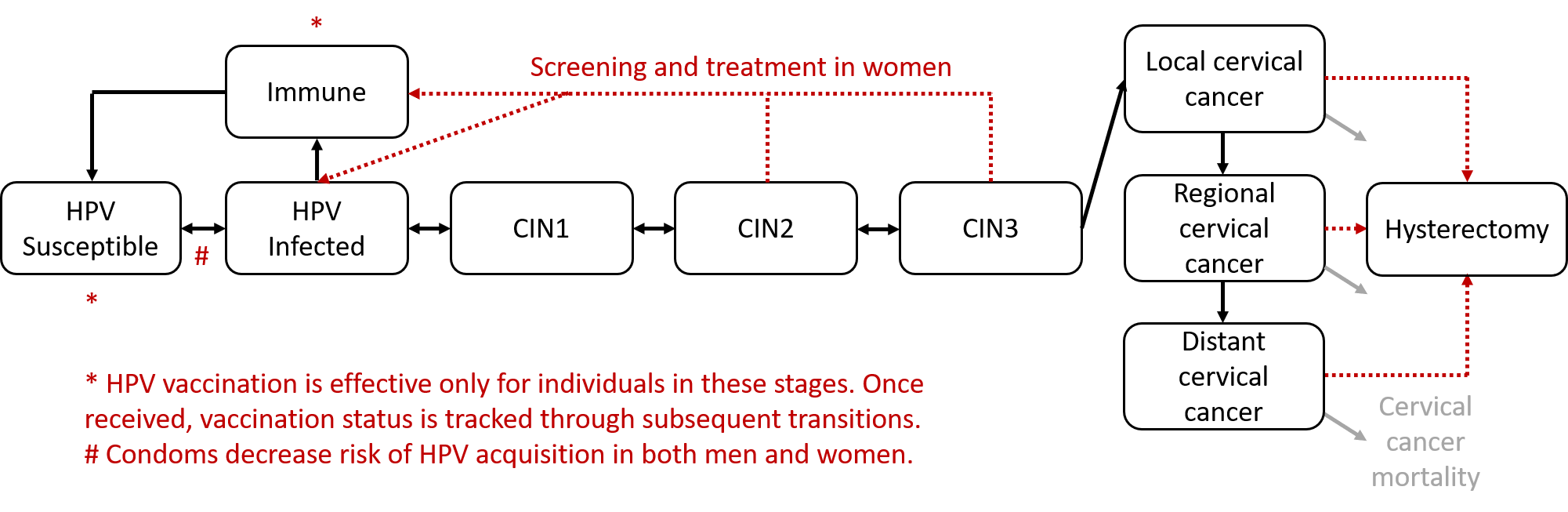


**Figure S6. Effects of HPV and cervical cancer-focused interventions on HPV natural history.**

### II.e.iv. Historical HPV vaccination and cervical cancer screening and treatment (pre-2021)

We model HPV vaccination beginning in 2014 for 57% of nine-year-old girls, consistent with last-dose coverage levels of the school-based national program in South Africa.^74^ Our model was designed to evaluate the impact of the nonavalent HPV vaccine, in that we group HPV infections into types corresponding to those covered by this vaccine (types 16, 18, 31, 33, 45, 52, and 58) and other oncogenic types. However, the current vaccination program in South Africa uses the bivalent vaccine, which targets only two of the seven oncogenic types in the nonavalent vaccine (types 16 and 18). To account for this, in the years 2014-2020 (when we assume bivalent vaccination is conducted), we adjust the 57% coverage by a factor of (0.7/0.9), based on evidence that HPV types 16 and 18 contribute to approximately 70% of cervical cancer cases relative to the 90% attributable to one or more of the types included in the nonavalent vaccine.^75^ In this calculation, we do not account for cross-protection against additional HPV types^75^. We assume that vaccination provides complete protection against the seven oncogenic types included in the nonavalent vaccine and 0% protection against other hrHPV types. The vaccine is ineffective for persons with an active nonavalent-type HPV infection at the time of vaccination. On the basis of evidence of the durability of the vaccine-induced immune response,^76^ we assume lifelong protection from vaccination. Vaccine efficacy and uptake are assumed not to vary by HIV status.

Beginning in 2000, we model once-per-lifetime cervical cancer screening for women in the age range of 35-39. Although South African policy during this time frame^77^ recommended screening every 10 years beginning at age 30, observed data suggest low compliance with this schedule.^78,79^ Beginning in 2011, recommendations additionally called for screening every 3 years in women living with HIV with low CD4 count, and for all women living with HIV from 2016 onwards, yet data do not show the expected increases in screening uptake corresponding with these changes^80^. Informed by consultation with in-country experts, we conservatively assume only one lifetime screen in the baseline scenario. Screening coverage increases linearly from 0% in 2000 to 18% of women aged 35-39 by 2003, and to 48% of women aged 35-39 by 2016^1^.

In line with South African screening guidelines,^81^ screening is with cytology (Papanicolaou smear) followed by colposcopy-guided biopsy for individuals with AS-CUS+ (Figure 3). Based on results of a meta-analysis,^82^ we assume that 57% of CIN2+ and cervical cancer cases are correctly identified. Of the women who test positive, 72% return for triage using colposcopic biopsy, and 51% of women with confirmed CIN2+ are treated.^83^ In this historical scenario, we assume that treatment is with large loop excision of the transformation zone (LLETZ, also referred to as a loop electrosurgical excision procedure or LEEP). Although cryotherapy is recommended for lesions that can be treated with ablative therapy, the availability of cryotherapy is limited in South Africa, such that we make the simplifying assumption of treatment with LLETZ only.^81^ Among women confirmed to have cervical cancer after triage, 40% return for hysterectomy treatment^83^. These rates reflect challenges with follow-up and retention for cervical cancer prevention and treatment, particularly with a multi-visit screening and treatment approach.^84-87^

Based on data from a recent meta-analysis, we assume that LLETZ successfully treats CIN2+ lesions 91% of the time in women without HIV and 77% of the time in women living with HIV^88^. Additionally, evidence suggests that 28% of women treated with LLETZ have persistent HPV infection, including cases with residual or recurrent CIN.^89^ Because this estimate is drawn from studies of primarily HIV-negative women, we used the estimate of 9% treatment failure in women without HIV above to calculate the percent of women who would have persistent HPV with successfully treated lesions (18.5%). Lacking data on differential HPV persistence by HIV status, we assume that HPV persistence after lesion clearance is the same among women living with HIV. Women with a successfully treated lesion who also clear HPV are assumed to develop temporary, partial immunity against reinfection with the same modeled HPV types present at the time of treatment (nonavalent vs. other hrHPV).

We assume that hysterectomy is 100% effective at treating cervical cancer. We do not account for treatment or recurrence of late-stage cancers that have spread to other organ systems. After hysterectomy, women are assumed to be infertile, unable to acquire or transmit HPV, and to have no increase in mortality due to their prior cancer status. Because the focus of our analysis is on cervical cancer incidence, we do not model symptomatic diagnosis of cervical cancer or treatment with other methods such as radiation or chemotherapy.

If a woman is coinfected with vaccine-type and non-vaccine-type hrHPV, her disease status is determined by her most advanced HPV type. We assume cervical screening and treatment to be equally effective for both type groups.

## II.f. Future scenarios for cervical cancer screening, treatment, and HPV vaccination

We model fourteen primary scenarios with differing strategies, coverage, and loss to follow-up for screening and treatment and HPV vaccination coverage (Table S21). The baseline scenario is a continuation of current/historical practice using cytology and colposcopy. Although guidelines call for repeat screening,^90^ we make a conservative assumption of once-lifetime screening between ages 35-39 at 48% coverage.^91,92^ In this scenario, 57% of girls age 9-14 are vaccinated,^93^ although we assume a switch from the bivalent to the nonavalent vaccine in 2021. If this change does not occur in reality, we would expect cervical cancer incidence to decline to a lesser extent than projected. However, we also assume no protection with a single dose of the vaccine; as of 2018, an estimated 71% of girls in South Africa had received at least one dose, and there is evidence that single-dose vaccination may provide substantial protection.^76,94^ In our first comparator scenario, effective vaccine coverage is scaled up to 90% in 2021, again with the nonavalent vaccine. If evidence more conclusively demonstrates the efficacy of single-dose vaccination, current levels of engagement would provide a solid foundation for reaching this benchmark. We assume the vaccine is 100% effective against covered types (HPV16/18/31/33/54/52/58) for girls who are uninfected with those types at the time of vaccination, and the duration of protection is lifelong.

**Table S21: Cervical cancer screening, treatment, and HPV vaccination in modeled scenarios**

|  | **Screening** | | | **Treatment** | | **Vaccination** | |
| --- | --- | --- | --- | --- | --- | --- | --- |
| **Scenario** | **Strategy** | **Ages** | **Coverage** | **Strategy** | **Retention*** | **Strategy** | **Coverage** |
| 0 | Cytology + colposcopy triage | 35 | 48% | Cryotherapy/ LLETZ | 72% return for colposcopy, 50% for treatment[3] | 9v, ages 9-14 | 57% (2017 coverage) ^93^ |
| 1 |  |  |  |  |  |  | 90% |
| 2 | HPV DNA testing |  |  | Thermal ablation/ LLETZ | 95% for TA; 80% for LLETZ |  |  |
| 3 | HPV DNA genotyping (HPV16/18/31/33/45/52/58) |  |  |  |  |  |  |
| 4 | AVE |  |  |  |  |  |  |
| 5 | HPV DNA with AVE triage |  |  |  |  |  |  |
| 6 | HPV DNA testing | 35, 45 |  |  |  |  |  |
| 7 | HPV DNA genotyping (HPV16/18/31/33/45/52/58) |  |  |  |  |  |  |
| 8 | AVE |  |  |  |  |  |  |
| 9 | HPV DNA with AVE triage |  |  |  |  |  |  |
| 10 | HPV DNA testing | HIV-: 35, 45  HIV+: every 5 years ages 25-49 |  |  |  |  |  |
| 11 | HPV DNA genotyping (HPV16/18/31/33/45/52/58) |  |  |  |  |  |  |
| 12 | AVE |  |  |  |  |  |  |
| 13 | HPV DNA with AVE triage |  |  |  |  |  |  |
| Abbreviations: AVE, automated visual evaluation; HPV, human papillomavirus; LLETZ, large loop excision of the transformation zone | | | | | | | |

The remaining twelve comparator scenarios model implementation of the single-visit strategies for screening and treatment shown in Table S21, all with 90% coverage with nonvalent HPV vaccination. While genotyping strategies commonly focus on HPV types 16, 18, and 45, our modeled strategy is based on evidence from a recent study in South Africa that concluded that treating women who test positive for any of the types covered by the nonavalent vaccine or HPV 35 optimized prevention goals for both HIV-uninfected women and women living with HIV.^95^ We modified this strategy to exclude HPV 35 because our model groups HPV 35 with other high-risk types.

For each of these strategies, we evaluate a scenario with once-lifetime screening (as in the baseline scenario), a scenario with twice-lifetime screening at ages 35-39 and 45-59, and with twice-lifetime screening at ages 35-39 and 45-49 for women without HIV and every 5 years between ages 25-49 for women with HIV. Although women with HIV are recommended to screen every 3 years,^90^ given the structure of our model with 5-year age groupings, we approximate this by implementing screening in each 5-year group. Additionally, because our model is compartmental and doesn’t track screening history, a random subset of individuals is screened in each age group. This implies that any given individual may not screen at each 5- or 10-year interval. In all scenarios, we assume that ART-naïve women living with HIV are equally likely to screen as treated women living with HIV. In reality, untreated women may be more difficult to engage in repeat screening,^96^ which would lessen the impact of the repeat screening strategies. All scenarios hold the proportion of women screened in each age group at 48%, as in the baseline scenario. In a sensitivity analysis, we explored the impact of increasing coverage to 70% with repeat HPV DNA testing, the most effective strategy (results presented in Figure S25, Table S33).

Based on a review of the literature, we defined model inputs governing the sensitivity and specificity of screening and diagnostic tests and the effectiveness of treatment to vary by HIV status. Table S22 shows the parameters that distinguish the interventions used in our scenarios. To isolate the impact of screening and diagnostic performance on model outcomes, we ran scenarios using the single-visit strategies with all other parameters (screening frequency, coverage, retention for treatment) the same as scenario 1 (results presented in Figure S24, Table S33). Most of the parameters listed were used as inputs to the model, however for HPV-based testing, the specificity estimates presented were derived from model results. Sensitivity was also derived for the HPV DNA genotyping strategy. As discussed in more detail below, because our model simulates and tracks HPV infection and progression through CIN states, we controlled test performance by specifying the proportion of individuals with any high-risk HPV infection (for primary HPV DNA testing) or with one of the types targeted by genotyping who would screen positive. We then derived the sensitivity and specificity presented in the table based on the prevalence of (type-specific) HPV infection and the proportion assumed to screen positive. Because the prevalence and type distribution of HPV changes over time and is variable across simulations, we present the median and range of estimates for these parameters in 2021 and in 2120.

**Table S22:** Key parameters for modeled scenarios, by groups defined by HIV status and viral suppression from antiretroviral therapy

|  | **HIV-negative women** | **Untreated women with HIV** | **Virally suppressed women with HIV^a^** | **Refs** |
| --- | --- | --- | --- | --- |
| **Screening and triage performance characteristics^b^** | | | |  |
| Cytology |  |  |  |  |
| Sensitivity^c^ | 0.57 | 0.52 | 0.55 | ^98,99,102^ |
| Specificity^c^ | 0.93 | 0.85 | 0.89 | ^98,99,102^ |
| Colposcopy |  |  |  |  |
| Sensitivity | 1.0 | 1.0 | 1.0 | ^Asm^ |
| Specificity | 1.0 | 1.0 | 1.0 | ^Asm^ |
| HPV DNA testing |  |  |  |  |
| Sensitivity | 0.85 | 0.94 | 0.90 | ^104^ |
| Specificity [median (range)]^d^ | 2021: 0.93 (0.81, 0.96)  2120: 0.97 (0.93, 1.00) | 2021: 0.71 (0.54, 0.88)  2120: 0.88 (0.79, 0.98) | 2021: 0.81 (0.66, 0.91)  2120: 0.93 (0.85, 0.99) | ^Derived^ |
| HPV DNA genotyping (16/18/31/33/45/52/58) |  |  |  |  |
| Sensitivity [median (range)]^d^ | 2021: 0.60 (0.46, 0.71)  2120: <0.01 (<0.01, 0.02) | 2021: 0.70 (0.54, 0.82)  2120: <0.01 (<0.01, 0.04) | 2021: 0.65 (0.54, 0.76)  2120: <0.01 (<0.01, 0.03) | ^Derived^ |
| Specificity [median (range)]^d^ | 2021: 0.97 (0.89, 0.98)  2120: 1.00 (1.00, 1.00) | 2021: 0.84 (0.70, 0.94)  2120: 1.00 (1.00, 1.00) | 2021: 0.90 (0.79, 0.96)  2120: 1.00 (1.00, 1.00) | ^Derived^ |
| Automated Visual Evaluation (AVE) |  |  |  |  |
| Sensitivity^e^ | 0.98 | 0.98 | 0.98 | ^109^ |
| Specificity^e^ | 0.84 | 0.78 | 0.81 | ^109,111-113^ |
| HPV DNA testing with AVE triage |  |  |  |  |
| Sensitivity^f^ | 0.83 | 0.92 | 0.88 | ^104,109^ |
| Specificity [median (range)]^d,f^ | 2021: 0.99 (0.97, 0.99)  2120: 0.99 (0.99, 1.00) | 2021: 0.94 (0.90, 0.97)  2120: 0.97 (0.95, 1.00) | 2021: 0.96 (0.93, 0.98)  2120: 0.99 (0.97, 1.00) | ^Derived^ |
| **Treatment** | | | |  |
| Treatment failure (all methods)^g^ | 9% | 23% | 16% | ^124^ |
| HPV persistence (ablative therapies)^h^ | 39% | 39% | 39% | ^125^ |
| HPV persistence (LLETZ)^h^ | 19% | 19% | 19% | ^125^ |
| **Loss to follow-up** | | | |  |
| Three-visit strategy |  |  |  |  |
| Colposcopy | 28% | 28% | 28% | ^126^ |
| Treatment | 50% | 50% | 50% | ^126^ |
| Single-visit strategies |  |  |  |  |
| Thermal ablation | 5% | 5% | 5% | ^Asm^ |
| LLETZ^i^ | 20% | 20% | 20% | ^Asm^ |
| Acronyms and abbreviations: Asm, assumption; AVE, automated visual evaluation; HPV, human papillomavirus; LLETZ, large loop excision of the transformation zone  ^a^Test performance and treatment efficacy for women with viral suppression is assumed to fall between values for HIV-negative and untreated HIV-positive populations; ^b^Sensitivity and specificity are defined with reference to CIN2+ as the disease state; ^c^Threshold of ASCUS+. ^d^Test performance is derived from the model and varies over time with changes in HPV prevalence and type distribution as HPV vaccination is scaled up, as described above. Here we present the median and range of derived test performance values across simulations from the combined one-time, two-time, and repeat screening scenarios with each screening strategy. ^e^There is limited information on the diagnostic accuracy of AVE,^109^ and we could not find estimates stratified by HIV status. As described above, based on evidence from studies evaluating other visual methods (VIA and digital cervicography) the base analysis assumes no difference in sensitivity by HIV status, but slightly lower specificity. Sensitivity analyses varied these assumptions (Table S23); ^f^We assume that the diagnostic performance of AVE as a primary screening strategy is the same when applied to individuals who screen positive for HPV; ^g^Treatment failure refers to the proportion treated with CIN who remain in that CIN state post-treatment. ^h^HPV persistence refers to the proportion of women treated whose lesions resolve but who remain HPV-positive; ^i^Based on evidence from screen-and-treat programs reporting high retention in treatment,^116-118^ we assume that 95% of screen-positive women with eligible lesions receive treatment with thermal ablation. Receipt of treatment is lower among women requiring referral to LLETZ.^85,118,127^ | | | | |

**Table S23:** Automated visual evaluation (AVE) screening and triage performance characteristics^a^ for sensitivity analyses, by groups defined by HIV status and viral suppression from antiretroviral therapy

|  | **HIV-negative women** | **Untreated women with HIV** | **Virally suppressed women with HIV**^b^ | **Refs** |
| --- | --- | --- | --- | --- |
| **Automated Visual Evaluation (AVE) as a primary screening strategy** | | | | |
| Main analysis | | | | |
| Sensitivity | 0.98 | 0.98 | 0.98 | ^109^ |
| Specificity | 0.84 | 0.78 | 0.81 | ^109,111-113^ |
| Relative 10% lower performance for untreated women with HIV (no change for HIV-negative women, virally suppressed women midway between) | | | | |
| Sensitivity | 0.98 | 0.88 | 0.93 | ^Asm^ |
| Specificity | 0.84 | 0.70 | 0.77 | ^Asm^ |
| Relative 10% lower performance for all | | | | |
| Sensitivity | 0.88 | 0.88 | 0.88 | ^Asm^ |
| Specificity | 0.76 | 0.70 | 0.73 | ^Asm^ |
| Relative 20% lower performance for untreated women with HIV, 10% lower for HIV-negative women, virally suppressed women midway between) | | | | |
| Sensitivity | 0.88 | 0.78 | 0.83 | ^Asm^ |
| Specificity | 0.76 | 0.62 | 0.69 | ^Asm^ |
| Relative 20% lower performance for all | | | | |
| Sensitivity | 0.78 | 0.78 | 0.78 | ^Asm^ |
| Specificity | 0.67 | 0.62 | 0.65 | ^Asm^ |
| **HPV DNA testing with AVE triage^c^** | | | | |
| Main analysis | | | | |
| Sensitivity^f^ | 0.83 | 0.92 | 0.88 | ^104,109^ |
| Specificity [median (range)]^d,f^ | 2021: 0.99 (0.97, 0.99)  2120: 0.99 (0.99, 1.00) | 2021: 0.94 (0.90, 0.97)  2120: 0.97 (0.95, 1.00) | 2021: 0.96 (0.93, 0.98)  2120: 0.99 (0.97, 1.00) | ^Derived^ |
| AVE relative 10% lower performance for untreated women with HIV (no change for HIV-negative women, virally suppressed women midway between) | | | | |
| Sensitivity^e^ | 0.83 | 0.83 | 0.84 | ^Asm^ |
| Specificity^e^ | 2021: 0.99 (0.98, 0.99)  2120: 0.99 (0.99, 1.00) | 2021: 0.90 (0.86, 0.94)  2120: 0.97 (0.94, 0.99) | 2021: 0.95 (0.92, 0.97)  2120: 0.98 (0.97, 1.00) | ^Asm^ |
| AVE Relative 10% lower for all | | | | |
| Sensitivity^e^ | 0.75 | 0.83 | 0.79 | ^Asm^ |
| Specificity^e^ | 2021: 0.99 (0.97, 0.99)  2120: 0.99 (0.99, 1.00) | 2021: 0.90 (0.86, 0.94)  2120: 0.97 (0.94, 0.99) | 2021: 0.94 (0.91, 0.96)  2120: 0.98 (0.96, 1.00) | ^Asm^ |
| AVE Relative 20% lower performance for untreated women with HIV, 10% lower for HIV-negative women, virally suppressed women midway between) | | | | |
| Sensitivity^e^ | 0.75 | 0.74 | 0.75 | ^Asm^ |
| Specificity^e^ | 2021: 0.99 (0.97, 0.99)  2120: 0.99 (0.99, 1.00) | 2021: 0.87 (0.83, 0.92)  2120: 0.96 (0.92, 0.99) | 2021: 0.93 (0.89, 0.95)  2120: 0.98 (0.96, 1.00) | ^Asm^ |
| AVE Relative 20% lower performance for all | | | | |
| Sensitivity^e^ | 0.67 | 0.74 | 0.71 | ^Asm^ |
| Specificity^e^ | 2021: 0.98 (0.96, 0.99)  2120: 0.99 (0.98, 1.00) | 2021: 0.87 (0.83, 0.92)  2120: 0.96 (0.92, 0.99) | 2021: 0.92 (0.88, 0.95)  2120: 0.97 (0.95, 1.00) | ^Asm^ |
| Acronyms and abbreviations: Asm, assumption; AVE, automated visual evaluation; HPV, human papillomavirus  ^a^Sensitivity and specificity are defined with reference to CIN2+ as the disease state; ^b^Test performance and treatment efficacy for women with viral suppression is assumed to fall between values for HIV-negative and untreated HIV-positive populations;  ^c^We assume that the diagnostic performance of AVE as a primary screening strategy is the same when applied to individuals who screen positive for HPV; ^d^Test performance is derived from the model and varies over time with changes in HPV prevalence and type distribution as HPV vaccination is scaled up, as described above. | | | | |

Detailed description of assumptions and sources used to derive model inputs and parameters related to screening and treatment

*Differences in screening test performance by HIV and ART status:*

There is mixed evidence on whether the performance of cytology depends on HIV status. Several studies have shown that overall agreement between cytology and histology is low for women living with HIV.^97,98^ Differences in sensitivity by HIV status in many of these studies have not been significant, but sample sizes have been small.^98-100^ There is suggestion of lower specificity among women living with HIV,^98,99^ and studies have also shown lower specificity with decreasing CD4 count.^101^ We assume slightly lower sensitivity and specificity in women living with HIV to capture these potential differences, as a key focus of this analysis is understanding the impact of cervical cancer prevention strategies in the context of high HIV prevalence. For diagnostic accuracy among HIV-uninfected women, we use the values reported by Arbyn et al.,^102^ as the included studies were in settings where HIV prevalence is low. We derive estimates for women living with HIV by applying the relative sensitivity^98,99^ and specificity^98^ observed by HIV status in available studies.

In the scenarios with cytology-based screening, we modeled triage with colposcopy. The South African guidelines recommend colposcopy with biopsy, but acknowledge that this may not always be feasible due to limited availability of equipment and laboratory care.^103^ However, for this analysis, we will conservatively model the guidelines.

HPV DNA testing consistently demonstrates lower specificity for women living with HIV, and there is a suggestion of higher sensitivity.^95,104,105^ There is also evidence that the specificity decreases with lower CD4 count among women living with HIV.^101,106,107^ We used estimates of sensitivity by HIV status from a meta-analysis by Kelly et al., ^104^ which align with estimates from other studies and reviews.^95,108^ These values determine the proportion of women in CIN2+ states who screen positive. We additionally made the simplifying assumption that these estimates of sensitivity for CIN2+ correspond to the proportion of individuals with HPV who test positive across disease states. We assume that no individuals without an active HPV infection test positive. We then derived the specificity for these strategies by counting the number of women in states $\leq$CIN1 who would screen negative divided by the total number of women in states $\leq$CIN1 who were screened.

For the HPV genotyping strategy, we applied the same sensitivity estimates used for HPV DNA testing to determine the proportion of women with an HPV16/18/31/33/45/52/58 infection who screen positive. That is, we assume that the likelihood of screening positive given an active HPV infection is the same across the modeled HPV type groups. As described above, we derive the specificity by taking the proportion of women screened in states $\leq$CIN1 who would screen negative (all women who are not infected with one of the specified HPV types and those who are infected with the specified types but who screen negative) divided by the total number of women in states $\leq$CIN1 who were screened. For HPV genotyping, we also derived sensitivity by dividing the number of women screened in states CIN2+ who test positive by the total number of women in states CIN2+ who screen.

As AVE is a relatively new technology, there is limited information on the diagnostic accuracy of this strategy,^109,110^ and we could not find estimates stratified by HIV status. Studies evaluating other visual strategies (VIA and/or digital cervicography (DC)) have reported inconsistent findings on sensitivity by HIV status, but there is a consistent signal of lower specificity for women living with HIV.^111-113^ As such, our base analysis assumes no difference in sensitivity by HIV status but slightly lower specificity. We conducted sensitivity analyses to explore a range of assumptions regarding diagnostic accuracy, including lower performance for women with HIV (Table S23; results presented in Figure S26 and Table S33). In the scenario using AVE as triage following a positive HPV test, we assumed that the performance of AVE is the same as when used as a primary screening strategy.

For all strategies in which inputs are stratified by HIV status, we assumed that estimates presented for WLHIV correspond to women with detectable viral load. On the basis of evidence that test performance for women with HIV who have higher CD4 count trends in the direction of performance for HIV-uninfected women,^101,107^ we assumed that test performance values for women with viral suppression fall between the estimates for women without HIV and women with untreated HIV.

*Treatment retention and effectiveness*

In these single-visit scenarios, treatment is with thermal ablation as an alternative to cryotherapy, or with LLETZ for women with lesions ineligible for ablative therapy. Studies have reported comparable effectiveness with cryotherapy and thermal ablation,^114,115^ but thermal ablation is more efficient and more scalable – it can be implemented using portable devices and does not require refrigerant gas, the need for which has limited access to treatment in some settings.^84,85^ We therefore assume that use of thermal ablation would facilitate higher retention for treatment following positive screening tests in these scenarios. Assumptions about retention are informed by studies reporting on the percent of women lost to follow-up in screen-and-treat programs. In several, these data showed retention of ~90% or higher,^116-118^ although others have reported retention of 43-66% of those offered cryotherapy.^84,85,119^ Those reporting lower retention highlight issues in service delivery specific to cryotherapy, suggesting that higher retention may be feasible with thermal ablation.^84,85^ Retention appears to be lower for LLETZ than cryotherapy, which is as expected given that LLETZ usually requires a referral.^85,118^ To explore the impact of lower retention, we conducted a sensitivity analysis with moderate loss to follow-up of 70% for thermal ablation and 50% for LLETZ (results presented in Figure S25, Table S33).

For HPV-based testing, we assume that a visual examination (i.e., visual inspection with acetic acid) will be performed to determine eligibility for ablative therapy. Studies have reported the percentage of women requiring excisional treatment (LLETZ) in the range of 12%-35%^115,116,119-123^ without stratification by disease stage. Although there is evidence that women living with HIV are more likely to have larger lesions ineligible for ablative therapy,^120^ it is not clear to what extent this reflects women living with HIV having more progressed disease at the time of screening. We thus assume that, within a CIN state, the proportion of women requiring LLETZ is the same across HIV status groups. We assume that 0% of women in CIN1, 10% of women in CIN2, and 30% of women in CIN3 require LLETZ.

In a meta-analysis by Debeaudrap et al.,^124^ treatment efficacy was similar for cryotherapy and LLETZ among women living with HIV. There were marginally significant differences when restricted to women treated for high-grade lesions, however, with more treatment failure following cryotherapy. Treatment failure was also higher, but non-significantly so, for treatment of high-grade compared to low-grade lesions. Overall, women living with HIV were twice as likely to experience treatment failure as HIV uninfected women, and this association was not statistically significantly different by treatment method. The authors did not have enough data to examine differences by ART status or CD4, but some studies included the review reported higher treatment failure with lower CD4 count. Studies comparing thermal ablation with cryotherapy have reported that these methods have similar efficacy as well, both overall and among WLHIV.^114,115^ For simplicity, we assumed the same treatment efficacy for all treatment methods and for all CIN states. We further assumed that estimates for women with HIV apply to women with untreated HIV, and treatment efficacy for women with viral suppression falls between values for women without HIV and those with untreated HIV.

In addition to treatment failure (resulting in women remaining in the CIN compartment in which they were treated), we accounted for persistent HPV post-treatment among those who clear their lesion as in the historical scenario. From a 2017 systematic review by Hoffman and colleagues, the median HPV persistence at 3 months post-treatment (including women with residual or recurrent CIN) is 48% for cryotherapy and 28% for LLETZ.^125^ It is worth noting that in this review, the cryotherapy estimate was based on data from only 1 study, and estimates of type-specific persistence are not presented by treatment method (overall, type-specific persistence was considerably lower than overall persistence at 18% vs. 28-30%). In a study in Zambia, the proportion of participants with persistent hrHPV post-treatment was similar for cryotherapy, thermal ablation, and LLETZ (60%, 58%, and 53%; p=0.48).^115^ However, the sample in this study was 50% HIV-positive and measures were taken at 6 months post-treatment, so the higher estimates may reflect re-infection as well as persistence. Despite the limitations, we used the estimates from Hoffman et al.^125^ and assumed that persistence is the same with thermal ablation as cryotherapy. The data in this review were from studies in primarily HIV-negative unknown status samples, however. Applying the same values for all women would mean that fewer WLHIV have persistent HPV after successful treatment, since the percent of women who have persistent HPV but are cleared of CIN is equal to the overall percent persistent minus (1- treatment efficacy), and treatment efficacy is lower for WLHIV. Lacking data on persistence stratified by HIV status, we used the data from this review to estimate the proportion of treated HIV-negative women who would have persistent HPV without CIN and applied that same percentage to women with HIV.

# III. Calibration and validation

## III.a. Calibration

We took a phased approach to model calibration. Briefly, we used hand-calibration to explore the sensitivity of model outcomes to individual parameters (Phase 0). This exploratory step informed our decision to divide the formal Bayesian calibration into two phases. In Phase 1, we used a Bayesian algorithm to fit 22 sexual behavior and HIV natural history parameters to observed demographic and HIV prevalence data. Randomly resampling from the 50 best-fitting parameter sets of Phase 1, we then used the same Bayesian algorithm to fit 23 HPV natural history parameters to observed data on HPV prevalence, CIN prevalence, cervical cancer incidence, and type distribution (Phase 2). The range of outcomes generated from the 25 best-fitting sets of Phase 2 and their corresponding resampled Phase 1 parameter values capture our uncertainty in model predictions.

### III.a.i. Phase 0

In greater detail, we first identified parameters for which empirical evidence was limited. We assumed a uniform prior distribution for each parameter, and we used published estimates and expert opinion to assign upper and lower bounds. By systematically varying parameters within the specified ranges, we verified that the parameters included in the calibration and their prior ranges could plausibly produce model results fitting the observed data. We also assessed the relative impact of individual parameters on different model outcomes. From this exploratory process, we determined that HIV outcomes are more sensitive to sexual behavior parameters than HPV-related outcomes, that HIV outcomes are tightly linked to demographic outcomes, and that pre-cancer and cancer outcomes are solely driven by the HPV natural history parameters. These findings allowed us to narrow prior ranges and divide our formal Bayesian calibration into two Phases, thereby increasing efficiency. To further increase efficiency, we fixed parameters with negligible impact on all model outcomes to the most robust estimates from literature.

### III.a.ii. Bayesian calibration approach

For Phases 1 and 2, we used the Approximate Bayesian Computation-Sequential Monte Carlo (ABC-SMC) algorithm to efficiently explore the multidimensional parameter space and identify parameter sets that generated model outcomes that best fit observed data.^128-130^ The ABC-SMC approach focuses sampling in the highest likelihood areas and avoids resampling the same parameter sets. Because a Bayesian calibration approach frames parameters as probabilistic and unknown, this allowed us to systematically estimate the range of outcomes given uncertainty in the underlying parameters.

We used the summed-log-likelihood as our metric of model fit, and maximum likelihood estimation (MLE) to evaluate the probability that potential parameter sets produced observed data. The point in multidimensional parameter space that maximizes the likelihood function is the most likely to have produced the observed trends in transmission of infection and development of disease. (Note: although ABC-SMC is an “approximate” Bayesian method in that it doesn’t require computation of a likelihood, we used the summed-log-likelihood as our fit metric.)

### III.a.iii. Phase 1

For the first ABC-SMC iteration, we fit 22 sexual behavior and HIV natural history parameters (Table S24) to observed demographic and HIV prevalence data (Table S25). We sampled a total of 91,980 parameter sets, from which we took the range of values for individual parameters from among the 50 best-fitting sets to define parameter uncertainty.

**Table S24: Phase 1 calibrated sexual behavior and HIV natural history parameters.** Values presented are the Mean [Uncertainty Range] of the 50 best-fitting parameter sets after Phase 1, and the Mean [Uncertainty Range] of the Phase 1 values corresponding to the 25 best-fitting parameter sets after Phase 2.

|  | **Parameter** | **Sexual risk group** | **Age group** | **Prior**  ***[lb, ub]*** | **Phase 1**  ***Mean [Uncertainty Range]*** | **Phase 2**  ***Mean [Uncertainty Range]*** | **References** |
| --- | --- | --- | --- | --- | --- | --- | --- |
| **1** | **Men, annual partnerships** | **High** | **15-19** | 0.20, 9.15 | 2.95  [0.25, 7.64] | 2.23  [0.27, 7.50] | Prior bounds were defined around observed data on male partnerships ^14^, with ranges informed by Phase 0. Partnerships for the 10-14 age group were set as 0.5x calibrated partnerships in the 15-19 age group. |
| **2** |  |  | **20-24** | 5.00, 18.30 | 10.88  [5.02, 18.23] | 12.33  [5.02, 17.32] |  |
| **3** |  |  | **25-29** | 6.25, 37.50 | 24.76  [7.45, 37.35] | 21.28  [8.99, 35.22] |  |
| **4** |  |  | **30-44** | 6.25, 37.50 | 19.76  [7.43, 36.89] | 20.32  [7.89, 34.78] |  |
| **5** |  |  | **45-79** | 5.00, 18.00 | 12.11  [5.24, 17.83] | 13.85  [5.24, 17.83] |  |
| **6** |  | **Moderate risk multiplier on high risk** | **15-79** | 0.11, 0.87 | 0.15  [0.11, 0.29] | 0.16  [0.11, 0.29] | Prior range informed by observed estimates and Phase 0. |
| **7** |  | **Low risk multiplier on moderate risk** | **15-79** | 0.15, 0.75 | 0.27  [0.16, 0.59] | 0.24  [0.16, 0.52] |  |
| **8** | **Women, annual partnerships** | **High** | **15-19** | 2.25, 13.50 | 9.39  [3.37, 13.36] | 10.07  [5.92, 13.34] | Prior bounds were defined around observed data on female partnerships ^14^, with ranges informed by Phase 0. Partnerships for the 10-14 age group were set as 0.5x calibrated partnerships in the 15-19 age group. |
| **9** |  |  | **20-24** | 6.50, 39.00 | 27.20  [7.18, 38.19] | 25.84  [13.18, 37.52] |  |
| **10** |  |  | **25-29** | 5.00, 28.50 | 13.09  [5.27, 28.28] | 10.70  [5.21, 26.69] |  |
| **11** |  |  | **30-44** | 5.00, 21.00 | 8.29  [5.09, 15.42] | 8.58  [5.31, 14.51] |  |
| **12** |  |  | **45-79** | 5.00, 15.00 | 10.50  [5.35, 14.89] | 9.33  [5.65, 14.89] |  |
| **13** |  | **Moderate risk multiplier on high risk** | **15-79** | 0.11, 0.87 | 0.51  [0.18, 0.86] | 0.47  [0.18, 0.86] | Prior range informed by observed estimates and Phase 0. |
| **14** |  | **Low risk multiplier on moderate risk** | **15-79** | 0.15, 0.75 | 0.49  [0.24, 0.74] | 0.49  [0.24, 0.73] |  |
| **15** | **Women, annual acts per partnership** | **Low** | **15-19** | 53.13, 88.56 | 67.65  [53.68, 87.50] | 65.71  [58.17, 76.37] | Prior range informed by Phase 0. Acts per partnership for the 10-14 age group were set as 0.5x the 15-19 age group. We assume acts per partnership for moderate and high-risk individuals are 0.60x acts in the next lowest risk group.  Phase 0 demonstrated that fit to observed HPV and CIN2/3 prevalence by age was sensitive to age trends in female acts. Among men, we assume those aged 10-19 have the same number of acts per partnership as women, whereas men aged 20-79 have equal acts to women of the next lowest age group, reflecting age disparities in relationships. |
| **16** |  |  | **20-24** | 52.47, 87.44 | 71.46  [53.73, 86.02] | 72.94  [54.24, 84.97] |  |
| **17** |  |  | **25-29** | 42.15, 70.25 | 56.07  [44.64, 69.02] | 55.39  [47.20, 62.32] |  |
| **18** |  |  | **30-44** | 40.95, 68.25 | 49.13  [41.04, 62.07] | 49.22  [41.04, 59.50] |  |
| **19** |  |  | **45-79** | 39.53, 65.88 | 52.82  [39.59, 65.05] | 54.02  [42.06, 64.40] |  |

| **20** | **Condom use in 2000 (x% of people * y% of the time)** | 0.11, 0.45 | 0.29  [0.11, 0.45] | 0.28  [0.16, 0.40] | Prior bounds were informed by data on self-reported condom use for contraception ^1^. The lower bound uses data from married women, and the upper bound uses data from women aged 20-24, who were the most sexually active. |
| --- | --- | --- | --- | --- | --- |
| **21** | **Mixing parameter by age**  (0 < $\epsilon_{a}$ < 1), where ($\epsilon_{a}=0)$ indicates completely off-diagonal mixing, and ($\epsilon_{a}=1)$ indicates completely random mixing | 0.10, 0.60 | 0.46  [0.13, 0.59] | 0.43  [0.13, 0.56] | Prior set according to expert opinion (Ruanne Barnabas, personal communications) and published literature^131,132^ indicating that mixing by age is more assortative than random. |
| **22** | **HIV transmission rate per act** | 0.0004, 0.0020 | 0.0008  [0.0006, 0.0013] | 0.0009  [0.0007, 0.0013] | Prior set according to data on female-to-male and male-to-female transmission in developed and developing countries ^46^, and narrowed in Phase 0 to exclude values inconsistent with observed cohort data. |

**Table S25: Phase 1 targets**

| **Phase 1: Demography calibration targets** | | | | | | | | |
| --- | --- | --- | --- | --- | --- | --- | --- | --- |
| **Criteria** | **Gender** | **Year** | **Age Group** | **Occurrences** | **N** | **Mean** | **Variance** | **Reference** |
| **Total population size** | **Men and women combined** | **2001** | **0-79** |  |  | 9348178 | 4.06E+10 | Statistics South Africa Census 2001 Post-enumeration survey, Table 2.7: Adjusted total population- full universe ^133^ |
|  | **Men and women combined** | **2011** | **0-79** |  |  | 10159121 | 1.21E+10 | Statistics South Africa Census 2011 Post-enumeration survey, Table 14: Adjusted total population- full universe ^134^ |
|  | **Men and women combined** | **2019** | **0-79** |  |  | 11192662 | 1.21E+10 |  |
| **Criteria** | **Gender** | **Year** | **Age Group** | **Occurrences** | **N** | **Mean** | **Variance** | **Reference** |
| **Population age distribution** | **Men and women combined** | **1996** | **0-4** | 964546 | 8244075 | 0.1170 | 1.25E-08 | Statistics South Africa Primary tables Census '96 and 2001 compared, Table 4.1: KwaZulu-Natal ^135^ |
|  |  |  | **5-9** | 1005944 | 8244075 | 0.1220 | 1.30E-08 |  |
|  |  |  | **10-14** | 1018218 | 8244075 | 0.1235 | 1.31E-08 |  |
|  |  |  | **15-19** | 914305 | 8244075 | 0.1109 | 1.20E-08 |  |
|  |  |  | **20-24** | 851953 | 8244075 | 0.1033 | 1.12E-08 |  |
|  |  |  | **25-29** | 684675 | 8244075 | 0.0831 | 9.24E-09 |  |
|  |  |  | **30-34** | 590583 | 8244075 | 0.0716 | 8.07E-09 |  |
|  |  |  | **35-39** | 504746 | 8244075 | 0.0612 | 6.97E-09 |  |
|  |  |  | **40-44** | 408926 | 8244075 | 0.0496 | 5.72E-09 |  |
|  |  |  | **45-49** | 337933 | 8244075 | 0.0410 | 4.77E-09 |  |
|  |  |  | **50-54** | 248877 | 8244075 | 0.0302 | 3.55E-09 |  |
|  |  |  | **55-59** | 212751 | 8244075 | 0.0258 | 3.05E-09 |  |
|  |  |  | **60-64** | 178471 | 8244075 | 0.0216 | 2.57E-09 |  |
|  |  |  | **65-69** | 158208 | 8244075 | 0.0192 | 2.28E-09 |  |
|  |  |  | **70-74** | 95372 | 8244075 | 0.0116 | 1.39E-09 |  |
|  |  |  | **75-79** | 68567 | 8244075 | 0.0083 | 1.00E-09 |  |
| **Population age distribution** | **Men and women combined** | **2011** | **0-4** | 1198134 | 10159121 | 0.1179 | 1.02E-08 | Statistics South Africa Statistical Release P0301 Community Survey 2016, Table 2.1: Census 2011 and Community Survey 2016 ^136^ |
|  |  |  | **5-9** | 1042528 | 10159121 | 0.1026 | 9.06E-09 |  |
|  |  |  | **10-14** | 1038857 | 10159121 | 0.1023 | 9.04E-09 |  |
|  |  |  | **15-19** | 1119535 | 10159121 | 0.1102 | 9.65E-09 |  |
|  |  |  | **20-24** | 1102388 | 10159121 | 0.1085 | 9.52E-09 |  |
|  |  |  | **25-29** | 980929 | 10159121 | 0.0966 | 8.59E-09 |  |
|  |  |  | **30-34** | 729230 | 10159121 | 0.0718 | 6.56E-09 |  |
|  |  |  | **35-39** | 612615 | 10159121 | 0.0603 | 5.58E-09 |  |
|  |  |  | **40-44** | 499102 | 10159121 | 0.0491 | 4.60E-09 |  |
|  |  |  | **45-49** | 454637 | 10159121 | 0.0448 | 4.21E-09 |  |
|  |  |  | **50-54** | 384397 | 10159121 | 0.0378 | 3.58E-09 |  |
|  |  |  | **55-59** | 325571 | 10159121 | 0.0320 | 3.05E-09 |  |
|  |  |  | **60-64** | 271326 | 10159121 | 0.0267 | 2.56E-09 |  |
|  |  |  | **65-69** | 175673 | 10159121 | 0.0173 | 1.67E-09 |  |
|  |  |  | **70-74** | 137821 | 10159121 | 0.0136 | 1.32E-09 |  |
|  |  |  | **75-79** | 86378 | 10159121 | 0.0085 | 8.30E-10 |  |
| **Population age distribution** | **Men and women combined** | **2019** | **0-4** | 1231101 | 11192662 | 0.1100 | 8.75E-09 | Statistics South Africa Statistical Release P0302 2019 Mid-year population estimates, Table 6 and Table 11 ^5^ |
|  |  |  | **5-9** | 1196909 | 11192662 | 0.1069 | 8.53E-09 |  |
|  |  |  | **10-14** | 1136163 | 11192662 | 0.1015 | 8.15E-09 |  |
|  |  |  | **15-19** | 980573 | 11192662 | 0.0876 | 7.14E-09 |  |
|  |  |  | **20-24** | 1006031 | 11192662 | 0.0899 | 7.31E-09 |  |
|  |  |  | **25-29** | 1061072 | 11192662 | 0.0948 | 7.67E-09 |  |
|  |  |  | **30-34** | 1008573 | 11192662 | 0.0901 | 7.33E-09 |  |
|  |  |  | **35-39** | 805405 | 11192662 | 0.0720 | 5.97E-09 |  |
|  |  |  | **40-44** | 613052 | 11192662 | 0.0548 | 4.63E-09 |  |
|  |  |  | **45-49** | 517032 | 11192662 | 0.0462 | 3.94E-09 |  |
|  |  |  | **50-54** | 423932 | 11192662 | 0.0379 | 3.26E-09 |  |
|  |  |  | **55-59** | 376538 | 11192662 | 0.0336 | 2.90E-09 |  |
|  |  |  | **60-64** | 306529 | 11192662 | 0.0274 | 2.38E-09 |  |
|  |  |  | **65-69** | 244864 | 11192662 | 0.0219 | 1.91E-09 |  |
|  |  |  | **70-74** | 178784 | 11192662 | 0.0160 | 1.40E-09 |  |
|  |  |  | **75-79** | 106104 | 11192662 | 0.0095 | 8.39E-10 |  |

| **Phase 1: HIV calibration targets** | | | | | | | | |
| --- | --- | --- | --- | --- | --- | --- | --- | --- |
| **Criteria** | **Gender** | **Year** | **Age Group** | **Cases** | **N** | **Mean** | **Variance** | **Reference** |
| **HIV Prevalence among Men** | **Men** | **2003** | **15-19** | 57 | 5624 | 0.0101 | 1.78E-06 | Africa Centre cohort (now on AHRI) data request ^14^ |
|  |  |  | **20-24** | 455 | 5141 | 0.0885 | 1.57E-05 |  |
|  |  |  | **25-29** | 1027 | 3643 | 0.2819 | 5.56E-05 |  |
|  |  |  | **30-34** | 1253 | 2773 | 0.4519 | 8.93E-05 |  |
|  |  |  | **35-39** | 728 | 1956 | 0.3722 | 1.19E-04 |  |
|  |  |  | **40-44** | 441 | 1638 | 0.2692 | 1.20E-04 |  |
|  |  |  | **45-49** | 309 | 1362 | 0.2269 | 1.29E-04 |  |
|  | **Men** | **2005** | **15-19** | 74 | 5624 | 0.0132 | 2.31E-06 |  |
|  |  |  | **20-24** | 519 | 5141 | 0.1010 | 1.77E-05 |  |
|  |  |  | **25-29** | 1214 | 3643 | 0.3332 | 6.10E-05 |  |
|  |  |  | **30-34** | 1243 | 2773 | 0.4483 | 8.92E-05 |  |
|  |  |  | **35-39** | 707 | 1956 | 0.3615 | 1.18E-04 |  |
|  |  |  | **40-44** | 527 | 1638 | 0.3217 | 1.33E-04 |  |
|  |  |  | **45-49** | 284 | 1362 | 0.2085 | 1.21E-04 |  |
|  | **Men** | **2006** | **15-19** | 41 | 5624 | 0.0073 | 1.29E-06 |  |
|  |  |  | **20-24** | 466 | 5141 | 0.0906 | 1.60E-05 |  |
|  |  |  | **25-29** | 1139 | 3643 | 0.3127 | 5.90E-05 |  |
|  |  |  | **30-34** | 1175 | 2773 | 0.4237 | 8.81E-05 |  |
|  |  |  | **35-39** | 815 | 1956 | 0.4167 | 1.24E-04 |  |
|  |  |  | **40-44** | 533 | 1638 | 0.3254 | 1.34E-04 |  |
|  |  |  | **45-49** | 394 | 1362 | 0.2893 | 1.51E-04 |  |
| **HIV Prevalence among Men** | **Men** | **2007** | **15-19** | 58 | 5624 | 0.0103 | 1.81E-06 |  |
|  |  |  | **20-24** | 552 | 5141 | 0.1074 | 1.86E-05 |  |
|  |  |  | **25-29** | 1106 | 3643 | 0.3036 | 5.80E-05 |  |
|  |  |  | **30-34** | 1116 | 2773 | 0.4025 | 8.67E-05 |  |
|  |  |  | **35-39** | 785 | 1956 | 0.4013 | 1.23E-04 |  |
|  |  |  | **40-44** | 648 | 1638 | 0.3956 | 1.46E-04 |  |
|  |  |  | **45-49** | 393 | 1362 | 0.2885 | 1.51E-04 |  |
|  | **Men** | **2008** | **15-19** | 58 | 5624 | 0.0103 | 1.81E-06 |  |
|  |  |  | **20-24** | 557 | 5141 | 0.1083 | 1.88E-05 |  |
|  |  |  | **25-29** | 1124 | 3643 | 0.3085 | 5.86E-05 |  |
|  |  |  | **30-34** | 1079 | 2773 | 0.3891 | 8.57E-05 |  |
|  |  |  | **35-39** | 911 | 1956 | 0.4657 | 1.27E-04 |  |
|  |  |  | **40-44** | 500 | 1638 | 0.3053 | 1.29E-04 |  |
|  |  |  | **45-49** | 370 | 1362 | 0.2717 | 1.45E-04 |  |
|  | **Men** | **2009** | **15-19** | 52 | 5624 | 0.0092 | 1.63E-06 |  |
|  |  |  | **20-24** | 403 | 5141 | 0.0784 | 1.41E-05 |  |
|  |  |  | **25-29** | 1010 | 3643 | 0.2772 | 5.50E-05 |  |
|  |  |  | **30-34** | 1271 | 2773 | 0.4583 | 8.95E-05 |  |
|  |  |  | **35-39** | 1081 | 1956 | 0.5527 | 1.26E-04 |  |
|  |  |  | **40-44** | 574 | 1638 | 0.3504 | 1.39E-04 |  |
|  |  |  | **45-49** | 498 | 1362 | 0.3656 | 1.70E-04 |  |
| **Criteria** | **Gender** | **Year** | **Age Group** | **Cases** | **N** | **Mean** | **Variance** | **Reference** |
| **HIV Prevalence among Women** | **Women** | **2003** | **15-19** | 555 | 5622 | 0.0987 | 1.58E-05 | Africa Centre cohort (now on AHRI) data request ^14^ |
|  |  |  | **20-24** | 1797 | 5489 | 0.3274 | 4.01E-05 |  |
|  |  |  | **25-29** | 1957 | 3869 | 0.5058 | 6.46E-05 |  |
|  |  |  | **30-34** | 1502 | 3174 | 0.4732 | 7.85E-05 |  |
|  |  |  | **35-39** | 844 | 2404 | 0.3511 | 9.48E-05 |  |
|  |  |  | **40-44** | 540 | 2075 | 0.2602 | 9.28E-05 |  |
|  |  |  | **45-49** | 387 | 1829 | 0.2116 | 9.12E-05 |  |
|  | **Women** | **2005** | **15-19** | 431 | 5622 | 0.0767 | 1.26E-05 |  |
|  |  |  | **20-24** | 1794 | 5489 | 0.3268 | 4.01E-05 |  |
|  |  |  | **25-29** | 1942 | 3869 | 0.5019 | 6.46E-05 |  |
|  |  |  | **30-34** | 1428 | 3174 | 0.4499 | 7.80E-05 |  |
|  |  |  | **35-39** | 909 | 2404 | 0.3781 | 9.78E-05 |  |
|  |  |  | **40-44** | 522 | 2075 | 0.2516 | 9.07E-05 |  |
|  |  |  | **45-49** | 370 | 1829 | 0.2023 | 8.82E-05 |  |
|  | **Women** | **2006** | **15-19** | 489 | 5622 | 0.0870 | 1.41E-05 |  |
|  |  |  | **20-24** | 1732 | 5489 | 0.3155 | 3.93E-05 |  |
|  |  |  | **25-29** | 1804 | 3869 | 0.4663 | 6.43E-05 |  |
|  |  |  | **30-34** | 1505 | 3174 | 0.4742 | 7.86E-05 |  |
|  |  |  | **35-39** | 875 | 2404 | 0.3640 | 9.63E-05 |  |
|  |  |  | **40-44** | 530 | 2075 | 0.2554 | 9.17E-05 |  |
|  |  |  | **45-49** | 320 | 1829 | 0.1750 | 7.89E-05 |  |
| **HIV Prevalence among Women** | **Women** | **2007** | **15-19** | 527 | 5622 | 0.0937 | 1.51E-05 |  |
|  |  |  | **20-24** | 1793 | 5489 | 0.3267 | 4.01E-05 |  |
|  |  |  | **25-29** | 2022 | 3869 | 0.5226 | 6.45E-05 |  |
|  |  |  | **30-34** | 1527 | 3174 | 0.4811 | 7.87E-05 |  |
|  |  |  | **35-39** | 898 | 2404 | 0.3735 | 9.73E-05 |  |
|  |  |  | **40-44** | 698 | 2075 | 0.3364 | 1.08E-04 |  |
|  |  |  | **45-49** | 361 | 1829 | 0.1974 | 8.66E-05 |  |
|  | **Women** | **2008** | **15-19** | 531 | 5622 | 0.0945 | 1.52E-05 |  |
|  |  |  | **20-24** | 1697 | 5489 | 0.3092 | 3.89E-05 |  |
|  |  |  | **25-29** | 1993 | 3869 | 0.5151 | 6.46E-05 |  |
|  |  |  | **30-34** | 1587 | 3174 | 0.5000 | 7.88E-05 |  |
|  |  |  | **35-39** | 968 | 2404 | 0.4027 | 1.00E-04 |  |
|  |  |  | **40-44** | 701 | 2075 | 0.3378 | 1.08E-04 |  |
|  |  |  | **45-49** | 491 | 1829 | 0.2685 | 1.07E-04 |  |
|  | **Women** | **2009** | **15-19** | 621 | 5622 | 0.1105 | 1.75E-05 |  |
|  |  |  | **20-24** | 1794 | 5489 | 0.3268 | 4.01E-05 |  |
|  |  |  | **25-29** | 1896 | 3869 | 0.4900 | 6.46E-05 |  |
|  |  |  | **30-34** | 1692 | 3174 | 0.5331 | 7.84E-05 |  |
|  |  |  | **35-39** | 1071 | 2404 | 0.4455 | 1.03E-04 |  |
|  |  |  | **40-44** | 778 | 2075 | 0.3749 | 1.13E-04 |  |
|  |  |  | **45-49** | 495 | 1829 | 0.2706 | 1.08E-04 |  |


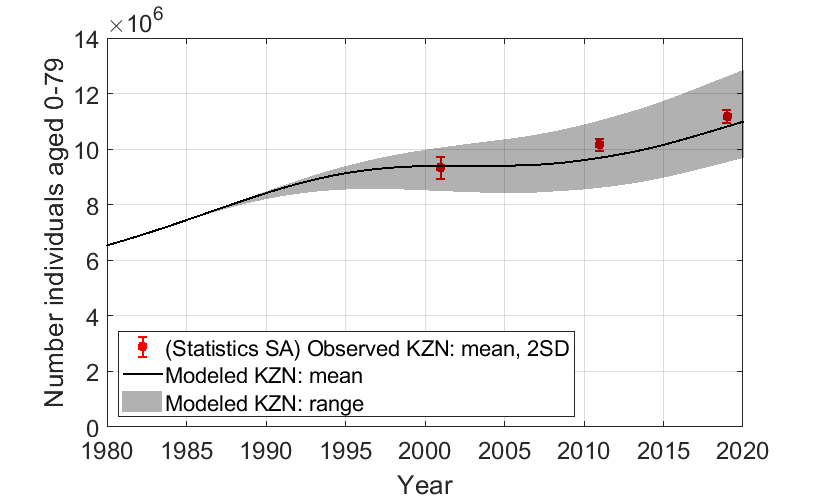


**Figure S7. Model fit to observed total population size data over time.** Standard deviation of the observed data calculated from the given absolute error assuming that the total population size follows a normal distribution. Shaded region represents the range of estimates using the 25 best-fitting model parameter sets.


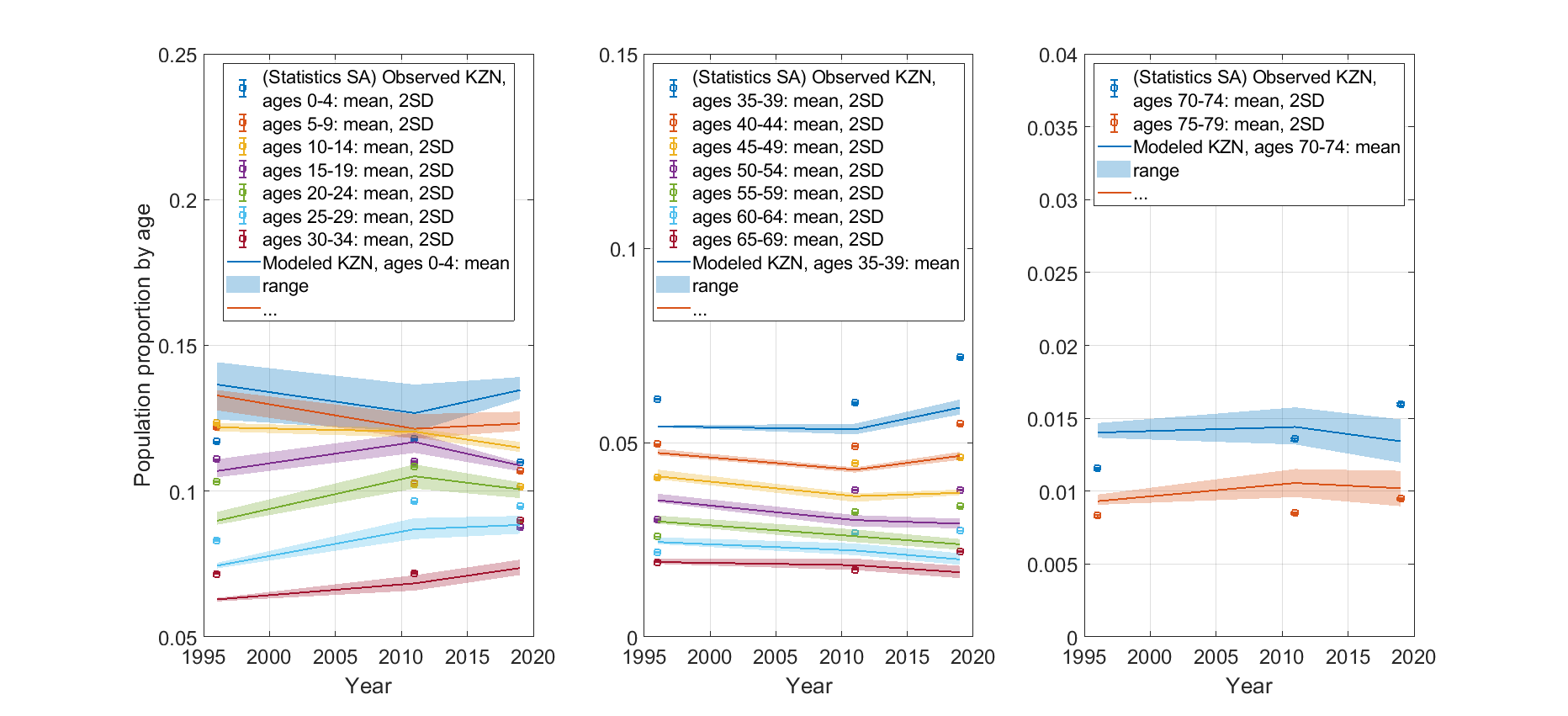


**Figure S8. Model fit to observed population age distribution data over time.** Standard deviation of the observed data calculated assuming that population proportions follow a normal approximation of the binomial distribution. Shaded regions represent the range of estimates using the 25 best-fitting model parameter sets.


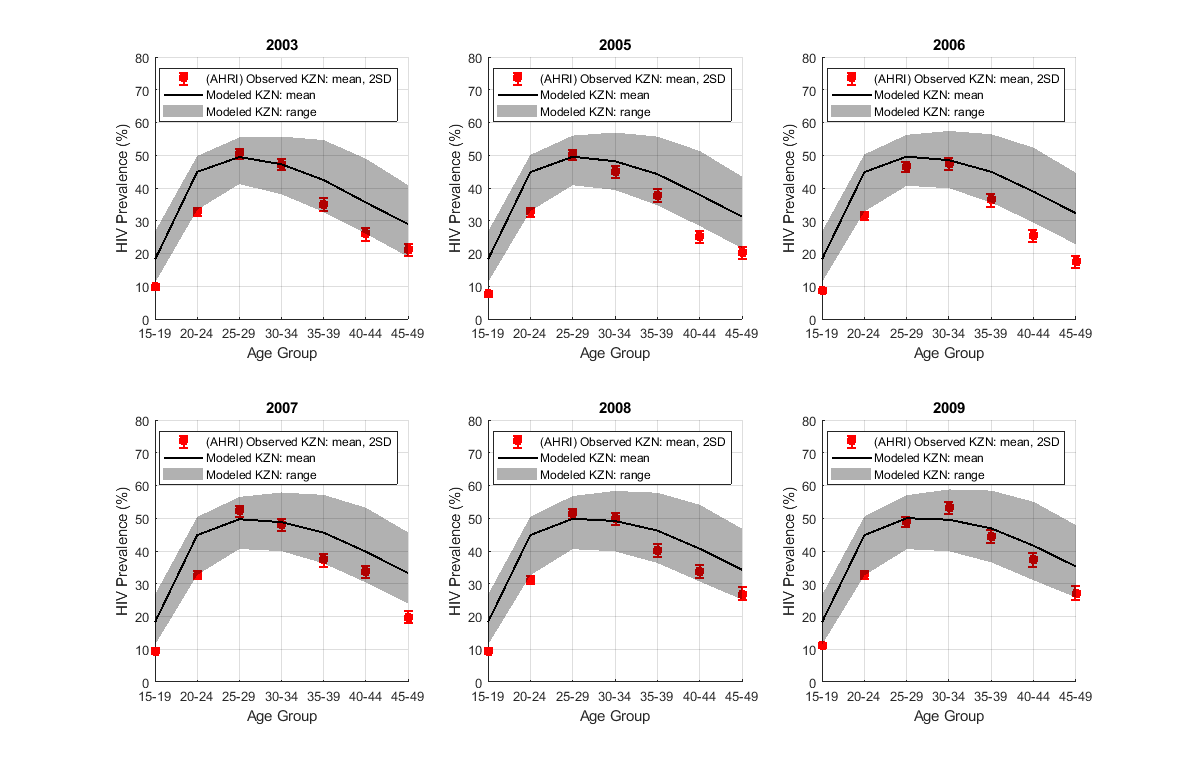


**Figure S9. Model fit to observed HIV prevalence data in women by age over time.** Standard deviation of the observed data calculated assuming that prevalence proportions follow a normal approximation of the binomial distribution. Shaded regions represent the range of estimates using the 25 best-fitting model parameter sets.


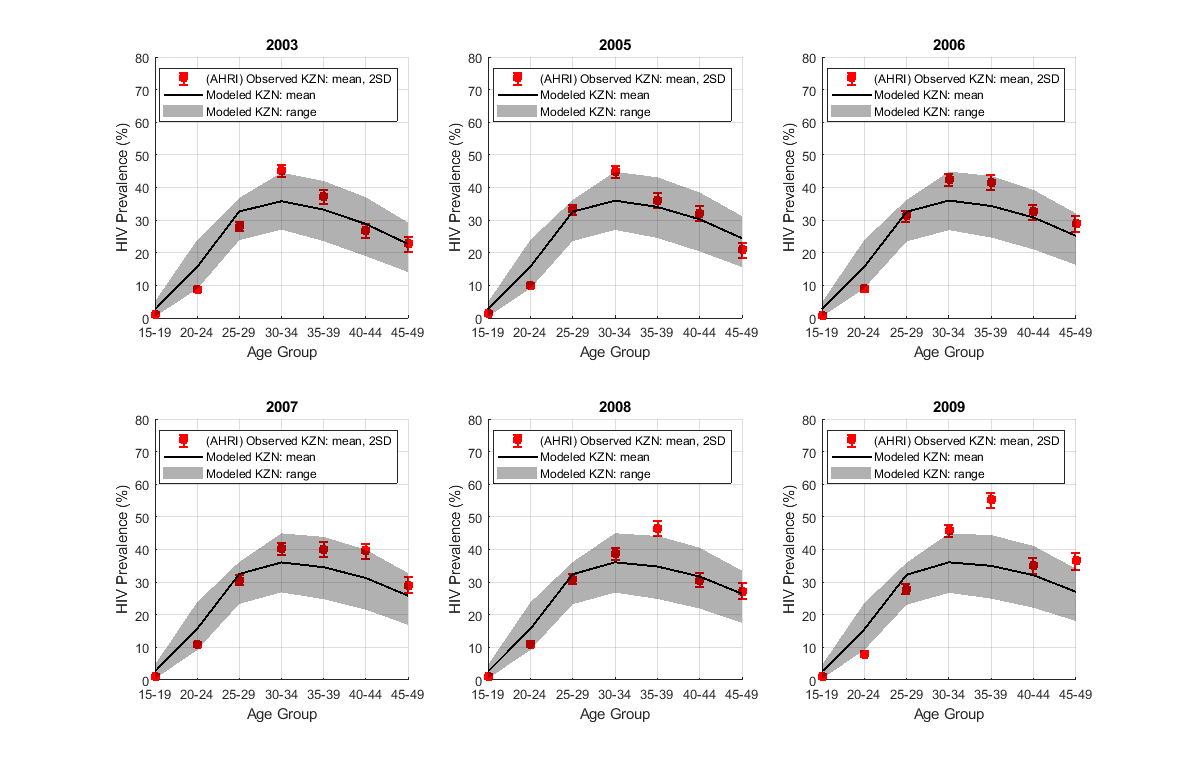


**Figure S10. Model fit to observed HIV prevalence data in men by age over time.** Standard deviation of the observed data calculated assuming that prevalence proportions follow a normal approximation of the binomial distribution. Shaded regions represent the range of estimates using the 25 best-fitting model parameter sets.

### III.a.iv. Phase 2

We then used ABC-SMC to fit 23 HPV natural history parameters (Table S26) to empirical data on HPV prevalence, CIN prevalence, cervical cancer incidence, and type distribution (Table S30). For each Phase 2 simulation, we randomly resampled the sexual behavior and HIV natural history parameters from the 50 best-fitting parameter sets of Phase 1. After sampling a total of 25,760 parameter sets, the range of individual parameters among the 25 best-fitting Phase 2 sets and their corresponding Phase 1 sampled values defined parameter uncertainty.

For cervical cancer incidence, we fit our model to 2018 GLOBOCAN estimates.^137^ Calculated by the International Agency for Research on Cancer (IARC), GLOBOCAN cancer incidence estimates are based on available measures of cancer incidence, mortality, and mortality-to-incidence ratios. Where registry data are limited for a given country, information from neighboring countries and/or other population groups are used. Registry data are incomplete in many low- and middle-income countries, such that the methods used to derive these estimates introduce uncertainty in the magnitude and age distribution of cases. We acknowledge the limitation of this approach, in that any biases in the GLOBOCAN estimates would be propagated in our model. However, given the lack of alternative reliable data on cervical cancer incidence, these estimates provided the most robust measures to use in refining our HPV natural history parameters.

**Table S26: Phase 2 calibrated HPV natural history parameters.** Values presented are Mean [Uncertainty Range] of the 25 best-fitting parameter sets after Phase 2.

|  | **Parameter** | | **Prior**  ***[lb, ub]*** | **Phase 2**  ***Mean [Uncertainty Range]*** | **References** |
| --- | --- | --- | --- | --- | --- |
| **1** | **HPV transmission rate per act** | | 0.001, 0.040 | 0.008  [0.003, 0.016] | Prior based on observed data ^138^, and narrowed in Phase 0 to exclude values inconsistent with observed cohort data. |
| **2** | **Multiplier for extent of natural immunity protection provided by recent type-specific infection clearance** | | 0.5, 1.0 | 0.7  [0.5, 1.0] | Prior lower bound based on observed data ^139^. |
| **3** | **HPV infection to CIN1 progression multiplier** | **Vaccine-type (vt)** | 0.4, 1.5 | 0.8  [0.4, 1.4] | Prior range based on exploration of a broad range of values in Phase 0. |
| **4** |  | **Non-vaccine-type (nvt)** | 0.5, 4.0 | 2.1  [0.8, 4.0] |  |
| **5** | **CIN1 to CIN2 progression multiplier** | **vt** | 1.0, 10.0 | 3.8  [1.4, 7.8] |  |
| **6** |  | **nvt** | 1.0, 20.0 | 9.3  [2.5, 18.8] |  |
| **7** | **CIN2 to CIN3 progression multiplier** | **vt** | 0.5, 3.0 | 1.6  [0.5, 2.8] |  |
| **8** |  | **nvt** | 1.0, 10.0 | 5.8  [2.1, 9.0] |  |
| **9** | **CIN3 to cervical cancer progression multiplier** | **vt** | 1.6, 16.0 | 10.6  [4.7, 16.0] |  |
| **10** |  | **nvt** | 8.0, 40.0 | 28.4  [12.0, 37.6] |  |
| **11** | **HPV infection clearance to naturally immune (women) or susceptible (men) multiplier** | **vt** | 0.8, 2.8 | 2.0  [1.3, 2.8] |  |
| **12** |  | **nvt** | 0.75, 1.8 | 1.4  [0.8, 1.8] |  |
| **13** | **CIN1 to HPV infection regression multiplier** | **vt** | 1.0, 3.5 | 2.1  [1.1, 3.4] |  |
| **14** |  | **nvt** | 0.1, 1.0 | 0.7  [0.2, 1.0] |  |
| **15** | **CIN2 to CIN1 regression multiplier** | **vt** | 0.05, 2.0 | 1.4  [0.8, 1.9] |  |
| **16** |  | **nvt** | 0.05, 1.0 | 0.6  [0.1, 0.8] |  |
| **17** | **CIN3 to CIN2 regression multiplier** | **vt** | 0.05, 1.5 | 0.8  [0.1, 1.4] |  |
| **18** |  | **nvt** | 0.05, 0.8 | 0.5  [0.2, 0.7] |  |
| **19** | **Multiplier on male clearance from HPV infection to susceptible** | | 1.0, 3.5 | 2.4  [1.2, 3.4] | Data suggests decreased HPV persistence among men^29^. This parameter also allows flexibility around assumptions that female-to-male and male-to-female transmission are equal, and that men have no natural immunity after HPV infection clearance. |
| **20** | **Multiplier on CIN1 to CIN2 progression for women living with HIV** | | 0.8, 1.5 | 1.2  [0.8, 1.5] | Prior range based on Phase 0 with a lower bound ≥0.5x so that the progression multiplier for women with HIV remains ≥1 for any CD4 stage. |
| **21** | **Multiplier on CIN2 to CIN3 progression for women living with HIV** | | 0.5, 1.0 | 0.7  [0.5, 1.0] |  |
| **22** | **Multiplier on CIN2 to CIN1 regression for women living with HIV** | | 0.75, 1.4 | 1.1  [0.8, 1.4] | Prior range based on Phase 0 with an upper bound ≤1.66x so that the regression multiplier for women with HIV does not exceed 1 for any CD4 stage. |
| **23** | **Multiplier on CIN3 to CIN2 regression for women living with HIV** | | 1.2, 1.66 | 1.5  [1.3, 1.7] |  |

**Table S27. Annual HPV clearance and precancer progression and regression rates.** Baseline values were derived from Cancer Council New South Wales estimates previously described by (Tan et al., 2018) in Supplement Table S24 ^3^. This set of estimates defines rates for HPV 16, 18, and other high-risk types, and includes additional state transitions. We therefore reweighted types and consolidated transitions to better match our HPV type grouping and HPV natural history model structure. For reweighting purposes, we assumed the type distribution of CIN2 to be the average of the type distributions for low-grade and high-grade lesions. **Age groupings modified from 10-24 and 25-49 in baseline values to 10-29 and 30-49 in calibrated values.*

| Progression rates | | | | | |
| --- | --- | --- | --- | --- | --- |
| Transition rate | **Age group** | **Baseline** | | **Calibrated**  ***Mean [Uncertainty Range]***  ***Calculated as (baseline transition rate) * (Mean [Uncertainty Range] of calibrated rate multiplier)*** | |
|  |  | **Vaccine-type** | **Non-vaccine-type** | **Vaccine-type** | **Non-vaccine-type** |
| HPV infection to CIN1 progression | **10-29*** | 0.2663 | 0.0830 | 0.2065  [0.1114, 0.3612] | 0.1746  [0.0680, 0.3263] |
|  | ***30-49** | 0.1303 | 0.0393 | 0.2168  [0.1170, 0.3793] | 0.1833  [0.0714,0.3427] |
|  | **50-69** | 0.0926 | 0.0277 | 0.2271  [0.1226, 0.3974] | 0.1921  [0.0748, 0.3590] |
|  | **70-79** | 0.0926 | 0.0277 | Equal to 50-69 rate | Equal to 50-69 rate |
| CIN1 to CIN2 progression | **10-29*** | 0.0651 | 0.0129 | 0.2476  [0.0902, 0.5084] | 0.1197  [0.0320, 0.2421] |
|  | ***30-49** | 0.0764 | 0.0152 | 0.2897  [0.1055, 0.5948] | 0.1400  [0.0374, 0.2833] |
|  | **50-69** | 0.0923 | 0.0185 | 0.3516  [0.1280, 0.7219] | 0.1699  0.0454, 0.3438] |
|  | **70-79** | 0.0923 | 0.0185 | Equal to 50-69 rate | Equal to 50-69 rate |
| CIN2 to CIN3 progression | **10-29*** | 0.1206 | 0.0225 | 0.1989  [0.0603, 0.3377] | 0.1305  [0.0482, 0.2016] |
|  | ***30-49** | 0.2157 | 0.0438 | 0.3182  [0.0965, 0.5403] | 0.2088  [0.0772, 0.3225] |
|  | **50-69** | 0.3068 | 0.0661 | 0.3978  [0.1206, 0.6754] | 0.2610  [0.0965, 0.4032] |
|  | **70-79** | 0.3670 | 0.0816 | Equal to 50-69 rate | Equal to 50-69 rate |
| CIN3 to cervical cancer progression | **10-29*** | 0.0012 | 0.0001 | 0.0127  [0.0056, 0.0191] | 0.0028  [0.0012, 0.0038] |
|  | ***30-49** | 0.0126 | 0.0015 | 0.0318  [0.0140, 0.0479] | 0.0071  [0.0030, 0.0094] |
|  | **50-69** | 0.0298 | 0.0037 | 0.1146  [0.0503, 0.1723] | 0.0256  [0.0108, 0.0338] |
|  | **70-79** | 0.0355 | 0.0045 | Equal to 50-69 rate | Equal to 50-69 rate |
| Regression and clearance rates | | | | | |
| Transition rate | **Age group** | **Baseline** | | **Calibrated**  ***Mean [Uncertainty Range]***  ***Calculated as (baseline transition rate) * (age multiplier) * (Mean [Uncertainty Range] of calibrated rate multiplier)*** | |
|  |  | **Vaccine-type** | **Non-vaccine-type** | **Vaccine-type** | **Non-vaccine-type** |
| HPV infection clearance to naturally immune (women) or susceptible (men) | **10-29*** | 0.6113 | 0.9307 | 1.2496  [0.7678, 1.7061] | 1.2617  [0.7745, 1.6579] |
|  | ***30-49** | 0.4804 | 0.7758 | 1.2496  [0.7678, 1.7061] | 1.2617  [0.7745, 1.6579] |
|  | **50-69** | 0.3091 | 0.5256 | 0.6373  [0.3950, 0.8456] | 0.6435  [0.3950, 0.8456] |
|  | **70-79** | 0.2761 | 0.4741 | Equal to 50-69 rate | Equal to 50-69 rate |
| CIN1 to HPV infection regression | **10-29*** | 0.2120 | 0.4550 | 0.4553  [0.2235, 0.7188] | 0.3171  [0.0979, 0.4448] |
|  | ***30-49** | 0.2120 | 0.4550 | 0.4553  [0.2235, 0.7188] | 0.3171  [0.0979, 0.4448] |
|  | **50-69** | 0.2120 | 0.4550 | 0.4553  [0.2235, 0.7188] | 0.3171  [0.0979, 0.4448] |
|  | **70-79** | 0.2120 | 0.4550 | Equal to 50-69 rate | Equal to 50-69 rate |
| CIN2 to CIN1 regression | **10-29*** | 0.3951 | 0.8604 | 0.5490  [0.3280, 0.7518] | 0.4772  [0.0878, 0.7241] |
|  | ***30-49** | 0.3951 | 0.8604 | 0.5490  [0.3280, 0.7518] | 0.4772  [0.0878, 0.7241] |
|  | **50-69** | 0.3951 | 0.8604 | 0.5490  [0.3280, 0.7518] | 0.4772  [0.0878, 0.7241] |
|  | **70-79** | 0.3951 | 0.8604 | Equal to 50-69 rate | Equal to 50-69 rate |
| CIN3 to CIN2 regression | **10-29*** | 0.1020 | 0.2531 | 0.0809  [0.0108, 1398] | 0.1160  [0.0538, 0.1841] |
|  | ***30-49** | 0.0787 | 0.1959 | 0.0647  [0.0086, 0.1118] | 0.0928  [0.0430, 0.1473] |
|  | **50-69** | 0.0278 | 0.0698 | 0.0485  [0.0065, 0.0839] | 0.0696  [0.0323, 0.1104] |
|  | **70-79** | 0.0143 | 0.0360 | Equal to 50-69 rate | Equal to 50-69 rate |

**Table S28. Age multipliers on transition rates.** Varying rates by age are used to approximate the increased risks associated with HPV persistence since the model does not track infection duration within individuals. The multipliers are applied to both HPV types and scaled up linearly across each age grouping. The age groupings assumed at baseline are consistent with Cancer Council New South Wales estimates previously described by (Tan et al., 2018 ) in Supplement Table S26 ^3^, and the baseline multiplier values are calculated as the average of the vaccine-type and non-vaccine-type relative risks after rate adjustment. However, we found these age groupings to be incongruent with observed cervical cancer incidence trends by age in South Africa, and therefore adjusted them during Phase 0 of calibration.

| Transition rate | Age multiplier | | | | |
| --- | --- | --- | --- | --- | --- |
|  | **Baseline** | | | **Adjusted** | |
|  | **25-49** | **50-69** | **70-79** | **30-49** | **50-79** |
| HPV infection to CIN1 progression | 0.49 | 0.35 | 0.35 | 1.05 | 1.10 |
| CIN1 to CIN2 progression | 1.17 | 1.42 | 1.42 | 1.17 | 1.42 |
| CIN2 to CIN3 progression | 1.79 | 2.54 | 3.04 | 1.60 | 2.00 |
| CIN3 to cervical cancer progression | 10.86 | 25.72 | 30.64 | 2.50 | 9.00 |
| HPV infection clearance to naturally immune (women) or susceptible (men) | 0.79 | 0.51 | 0.45 | 1.00 | 0.51 |
| CIN1 to HPV infection regression | 1.00 | 1.00 | 1.00 | 1.00 | 1.00 |
| CIN2 to CIN1 regression | 1.00 | 1.00 | 1.00 | 1.00 | 1.00 |
| CIN3 to CIN2 regression | 0.77 | 0.27 | 0.14 | 0.80 | 0.60 |

**Table S29.** **CIN progression and regression multipliers for women living with HIV.** Baseline progression multipliers based on (Liu et al., 2018) and calibrated to the KwaZulu-Natal setting by Tan et al., 2018.^3^

| Baseline | | | | | |
| --- | --- | --- | --- | --- | --- |
|  | **CD4 Stage** | | | | |
|  | **On ART** | **CD4 ≥500** | **CD4 350-500** | **CD4 200-350** | **CD4 ≤200** |
| CIN1 to CIN2 progression | 1.00 | 1.00 | 1.90 | 2.40 | 2.70 |
| CIN2 to CIN3 progression | 1.00 | 1.00 | 2.00 | 2.70 | 3.50 |
| CIN2 to CIN1 regression | 0.60 | 0.60 | 0.55 | 0.45 | 0.30 |
| CIN3 to CIN2 regression | 0.60 | 0.60 | 0.55 | 0.45 | 0.30 |
| Calibrated  *Mean [Uncertainty Range]*  *Calculated as (baseline multiplier) * (Mean [Uncertainty Range] of calibrated multiplier)* | | | | | |
|  | **CD4 Stage** | | | | |
|  | **On ART** | **CD4 ≥500** | **CD4 350-500** | **CD4 200-350** | **CD4 ≤200** |
| CIN1 to CIN2 progression | 1.00 | 1.00 | 2.20  [1.53, 2.76] | 2.77  [1.93, 3.49] | 3.12  [2.17, 3.92] |
| CIN2 to CIN3 progression | 1.00 | 1.00 | 1.41  [1.01, 1.97] | 1.91  [1.36, 2.66] | 2.47  [1.76, 3.44] |
| CIN2 to CIN1 regression | 0.65  [0.46, 0.81] | 0.65  [0.46, 0.81] | 0.59  [0.43, 0.74] | 0.49  [0.35, 0.61] | 0.32  [0.23, 0.41] |
| CIN3 to CIN2 regression | 0.89  [0.75, 0.99] | 0.89  [0.75, 0.99] | 0.82  [0.69, 0.91] | 0.67  [0.56, 0.75] | 0.45  [0.38, 0.50] |

**Table S31: Phase 2 targets**

| **Phase 2: HPV calibration targets** | | | | | | | | |
| --- | --- | --- | --- | --- | --- | --- | --- | --- |
| **Criteria** | **HIV Status** | **Year** | **Age Group** | **Cases** | **N** | **Mean** | **Variance** | **Reference** |
| **HPV Prevalence (including CIN) among Women** | **HIV-positive** | **2002** | **17-19** | 36 | 48 | 0.7500 | 3.91E-03 | ^140^ |
|  |  |  | **20-24** | 134 | 221 | 0.6063 | 1.08E-03 |  |
|  |  |  | **25-29** | 145 | 243 | 0.5967 | 9.90E-04 |  |
|  |  |  | **30-34** | 96 | 175 | 0.5486 | 1.42E-03 |  |
|  |  |  | **35-39** | 189 | 407 | 0.4644 | 6.11E-04 |  |
|  |  |  | **40-44** | 62 | 147 | 0.4218 | 1.66E-03 |  |
|  |  |  | **45-49** | 33 | 76 | 0.4342 | 3.23E-03 |  |
|  |  |  | **50-54** | 15 | 28 | 0.5357 | 8.88E-03 |  |
|  |  |  | **55-65** | 9 | 26 | 0.3462 | 8.71E-03 |  |
|  | **HIV-negative** | **2002** | **17-19** | 115 | 191 | 0.6021 | 1.25E-03 | ^140^ |
|  |  |  | **20-24** | 261 | 693 | 0.3766 | 3.39E-04 |  |
|  |  |  | **25-29** | 158 | 662 | 0.2387 | 2.74E-04 |  |
|  |  |  | **30-34** | 135 | 666 | 0.2027 | 2.43E-04 |  |
|  |  |  | **35-39** | 439 | 2272 | 0.1932 | 6.86E-05 |  |
|  |  |  | **40-44** | 247 | 1400 | 0.1764 | 1.04E-04 |  |
|  |  |  | **45-49** | 130 | 982 | 0.1324 | 1.17E-04 |  |
|  |  |  | **50-54** | 102 | 617 | 0.1653 | 2.24E-04 |  |
|  |  |  | **55-65** | 83 | 567 | 0.1464 | 2.20E-04 |  |
| **HPV Prevalence among Men** | **HIV-positive** | **2008** | **18-25** | 6 | 8 | 0.7500 | 2.34E-02 | ^141^ |
|  |  |  | **26-35** | 39 | 63 | 0.6190 | 3.74E-03 |  |
|  |  |  | **36-45** | 33 | 66 | 0.5000 | 3.79E-03 |  |
|  |  |  | **46-66** | 6 | 21 | 0.2857 | 9.72E-03 |  |
|  | **HIV-negative** | **2008** | **18-25** | 15 | 35 | 0.4286 | 7.00E-03 | ^141^ |
|  |  |  | **26-35** | 22 | 93 | 0.2366 | 1.94E-03 |  |
|  |  |  | **36-45** | 22 | 101 | 0.2178 | 1.69E-03 |  |
|  |  |  | **46-66** | 16 | 84 | 0.1905 | 1.84E-03 |  |

| **Phase 2: CIN calibration targets** | | | | | | | | |
| --- | --- | --- | --- | --- | --- | --- | --- | --- |
| **Criteria** | **HIV Status** | **Year** | **Age Group** | **Cases** | **N** | **Mean** | **Variance** | **Reference** |
| **CIN2/CIN3 Prevalence** | **HIV-positive** | **2002** | **17–19** | 6 | 48 | 0.1250 | 2.28E-03 | ^140^ |
|  |  |  | **20–24** | 12 | 221 | 0.0543 | 2.32E-04 |  |
|  |  |  | **25–29** | 31 | 243 | 0.1276 | 4.58E-04 |  |
|  |  |  | **30–34** | 27 | 175 | 0.1543 | 7.46E-04 |  |
|  |  |  | **35–39** | 33 | 407 | 0.0811 | 1.83E-04 |  |
|  |  |  | **40–44** | 8 | 147 | 0.0544 | 3.50E-04 |  |
|  |  |  | **45–49** | 6 | 76 | 0.0789 | 9.57E-04 |  |
|  |  |  | **50–54** | 2 | 28 | 0.0714 | 2.37E-03 |  |
|  |  |  | **55–59** | 1 | 13 | 0.0769 | 5.46E-03 |  |
|  |  |  | **60–65** | 1 | 13 | 0.0769 | 5.46E-03 |  |
|  | **HIV-negative** | **2002** | **17–19** | 3 | 191 | 0.0157 | 8.09E-05 | ^140^ |
|  |  |  | **20–24** | 19 | 693 | 0.0274 | 3.85E-05 |  |
|  |  |  | **25–29** | 14 | 662 | 0.0211 | 3.13E-05 |  |
|  |  |  | **30–34** | 24 | 666 | 0.0360 | 5.22E-05 |  |
|  |  |  | **35–39** | 65 | 2272 | 0.0286 | 1.22E-05 |  |
|  |  |  | **40–44** | 44 | 1400 | 0.0314 | 2.17E-05 |  |
|  |  |  | **45–49** | 30 | 982 | 0.0305 | 3.02E-05 |  |
|  |  |  | **50–54** | 13 | 617 | 0.0211 | 3.34E-05 |  |
|  |  |  | **55–59** | 4 | 283.5 | 0.0141 | 4.91E-05 |  |
|  |  |  | **60–65** | 4 | 283.5 | 0.0141 | 4.91E-05 |  |
| **CIN1 Prevalence** | **HIV-positive** | **2015** | **30-65** | 45 | 333 | 0.1351 | 3.51E-04 | ^142^ |
|  | **HIV-negative** |  |  | 28 | 382 | 0.0733 | 1.78E-04 |  |
| **CIN2 Prevalence** | **HIV-positive** | **2015** | **30-65** | 28 | 333 | 0.0841 | 2.31E-04 |  |
|  | **HIV-negative** |  |  | 8 | 382 | 0.0209 | 5.37E-05 |  |
| **CIN3 Prevalence** | **HIV-positive** | **2015** | **30-65** | 23 | 333 | 0.0691 | 1.93E-04 |  |
|  | **HIV-negative** |  |  | 10 | 382 | 0.0262 | 6.67E-05 |  |

To approximate cervical cancer incidence in KZN rather than South Africa nationally, we adjusted the GLOBOCAN 2018^137^ rates by age to take into account higher HIV prevalence in KZN. We assumed women with HIV have four times increased risk of cervical cancer^30^ and then reweighted the overall cervical cancer incidence rate according to the HIV prevalence in KZN compared to SA nationally. HIV prevalence data in older ages are sparse, so we assumed prevalence is increased by the same proportion in women aged 50+.

| **Phase 2: Cervical cancer calibration targets** | | | | | | | |
| --- | --- | --- | --- | --- | --- | --- | --- |
| **Criteria** | **HIV Status** | **Year** | **Age Group** | **Mean** | **Variance** | **Mean adjusted for KZN** | **Reference** |
| **Cervical cancer incidence per 100K women** | **All** | **2018** | **15–19** | 3.91 | 3.91 | 4.25 | ^137^ |
|  |  |  | **20–24** | 19.72 | 19.72 | 26.31 |  |
|  |  |  | **25–29** | 35.82 | 35.82 | 44.29 |  |
|  |  |  | **30–34** | 53.75 | 53.75 | 74.76 |  |
|  |  |  | **35–39** | 71.18 | 71.18 | 96.94 |  |
|  |  |  | **40–44** | 85.09 | 85.09 | 109.33 |  |
|  |  |  | **45–49** | 95.44 | 95.44 | 149.82 |  |
|  |  |  | **50–54** | 96.10 | 96.10 | 150.85 |  |
|  |  |  | **55–59** | 94.85 | 94.85 | 148.61 |  |
|  |  |  | **60–64** | 97.49 | 97.49 | 152.74 |  |
|  |  |  | **65-69** | 101.99 | 101.99 | 159.80 |  |
|  |  |  | **70-74** | 110.43 | 110.43 | 173.02 |  |

| **Phase 2: HPV type distribution calibration targets** | | | | | | |
| --- | --- | --- | --- | --- | --- | --- |
| **Criteria** | **HPV Type** | **Year** | **Age Group** | **Mean** | **Variance** | **Reference** |
| **HPV** | **9v HPV** | **2011-2015** | **0-79** | 0.4682 | 1.05E-04 | ^34,41^ |
|  | **Non-9v HPV** |  |  | 0.5318 | 1.05E-04 |  |
| **CIN1** | **9v HPV** | **2011-2015** | **0-79** | 0.5192 | 1.02E-03 | ^34,40^ |
|  | **Non-9v HPV** |  |  | 0.4808 | 1.02E-03 |  |
| **CIN3** | **9v HPV** | **2011-2015** | **0-79** | 0.7371 | 2.46E-04 | ^34,38,39^ |
|  | **Non-9v HPV** |  |  | 0.2629 | 2.46E-04 |  |
| **Cervical cancer** | **9v HPV** | **2011-2015** | **0-79** | 0.8578 | 9.59E-05 | ^34-37^ |
|  | **Non-9v HPV** |  |  | 0.1422 | 9.59E-05 |  |


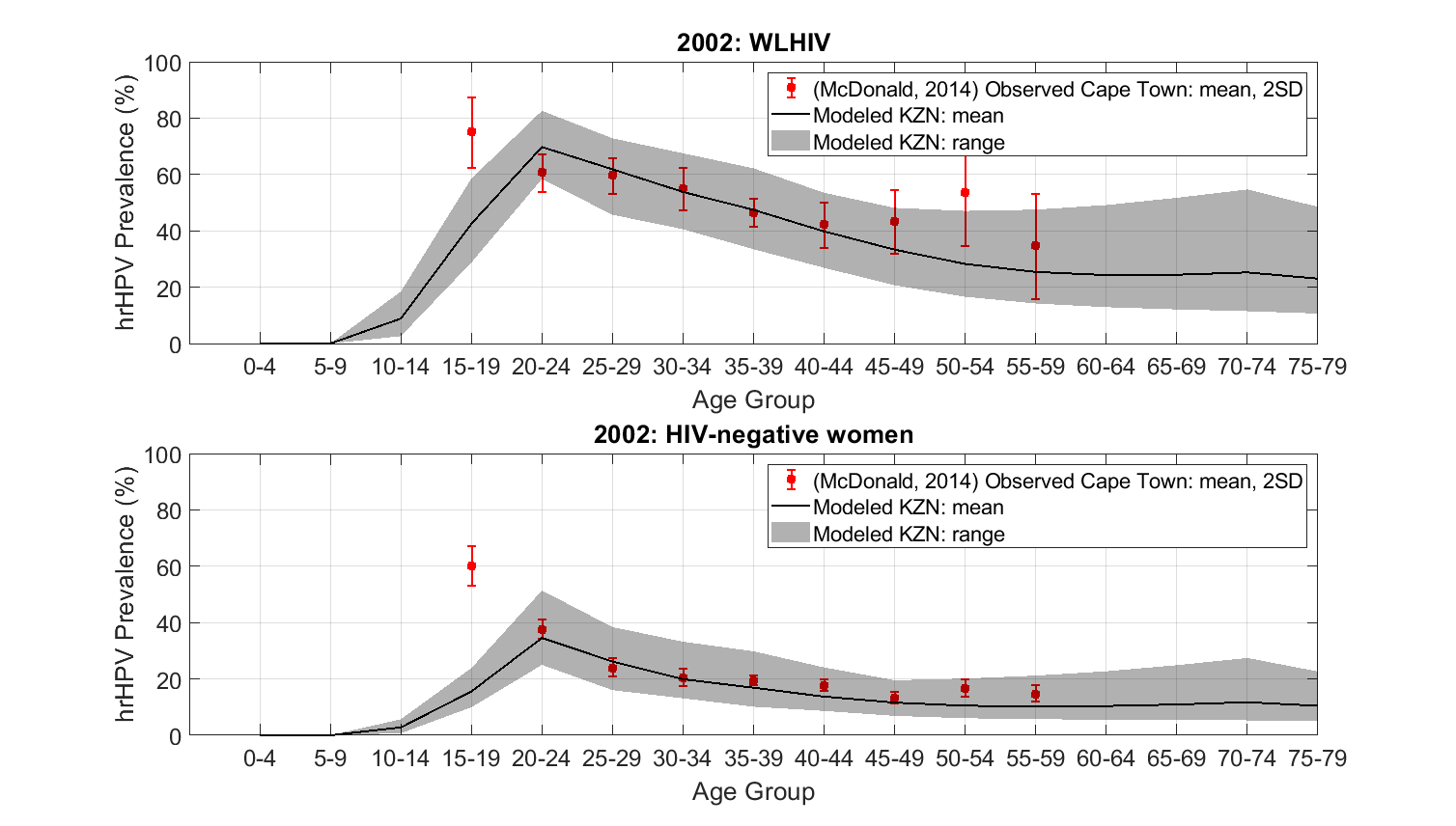


**Figure S11. Model fit to observed HPV prevalence data in women by age and HIV status in 2002.** Standard deviation of the observed data calculated assuming that prevalence proportions follow a normal approximation of the binomial distribution. Shaded regions represent the range of estimates using the 25 best-fitting model parameter sets.

***
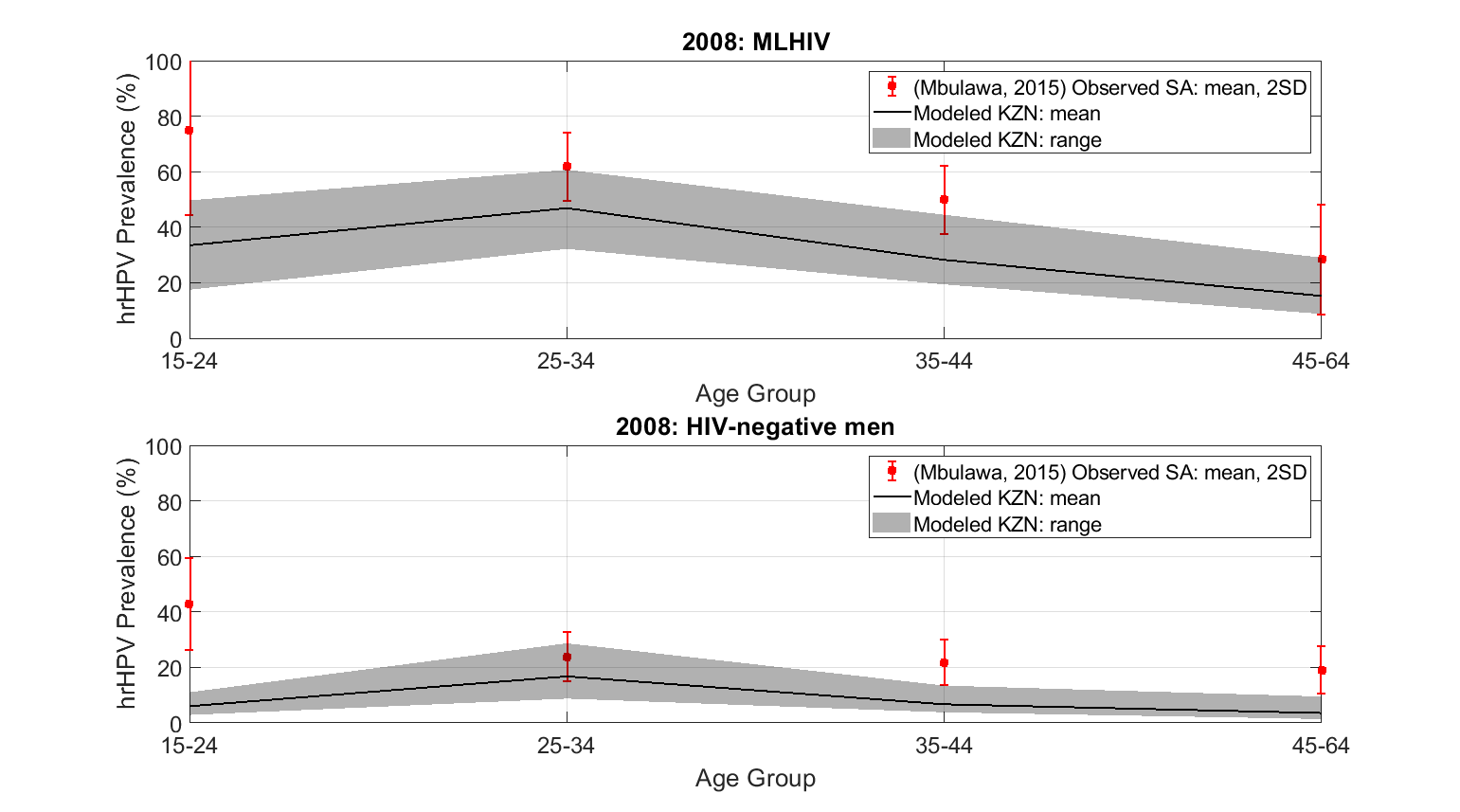
***

**Figure S12. Model fit to observed HPV prevalence data in men by age and HIV status in 2008.** Standard deviation of the observed data calculated assuming that prevalence proportions follow a normal approximation of the binomial distribution. Shaded regions represent the range of estimates using the 25 best-fitting model parameter sets.

***
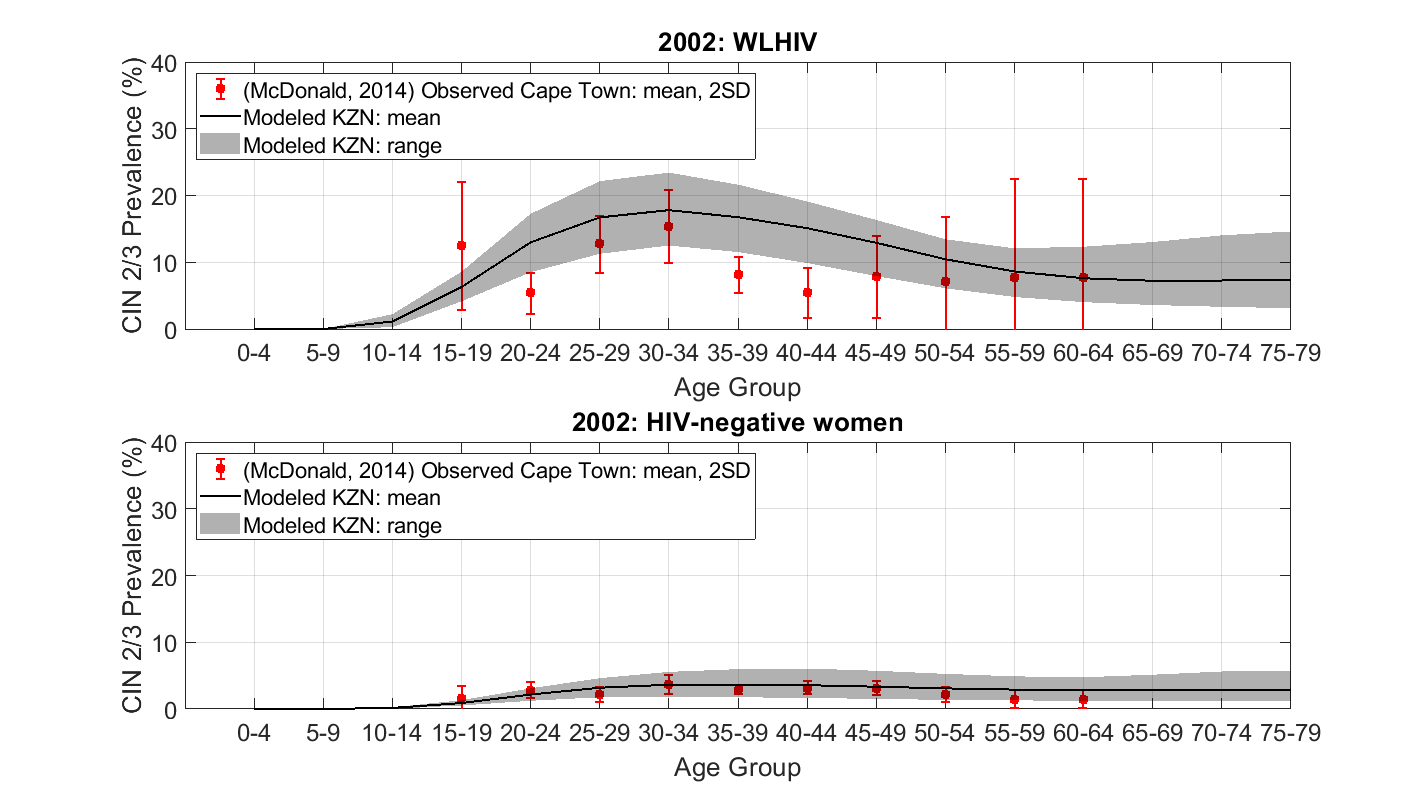
***

**Figure S13. Model fit to observed CIN2/3 prevalence data by age and HIV status in 2002.** Standard deviation of the observed data calculated assuming that prevalence proportions follow a normal approximation of the binomial distribution. Shaded regions represent the range of estimates using the 25 best-fitting model parameter sets.

***
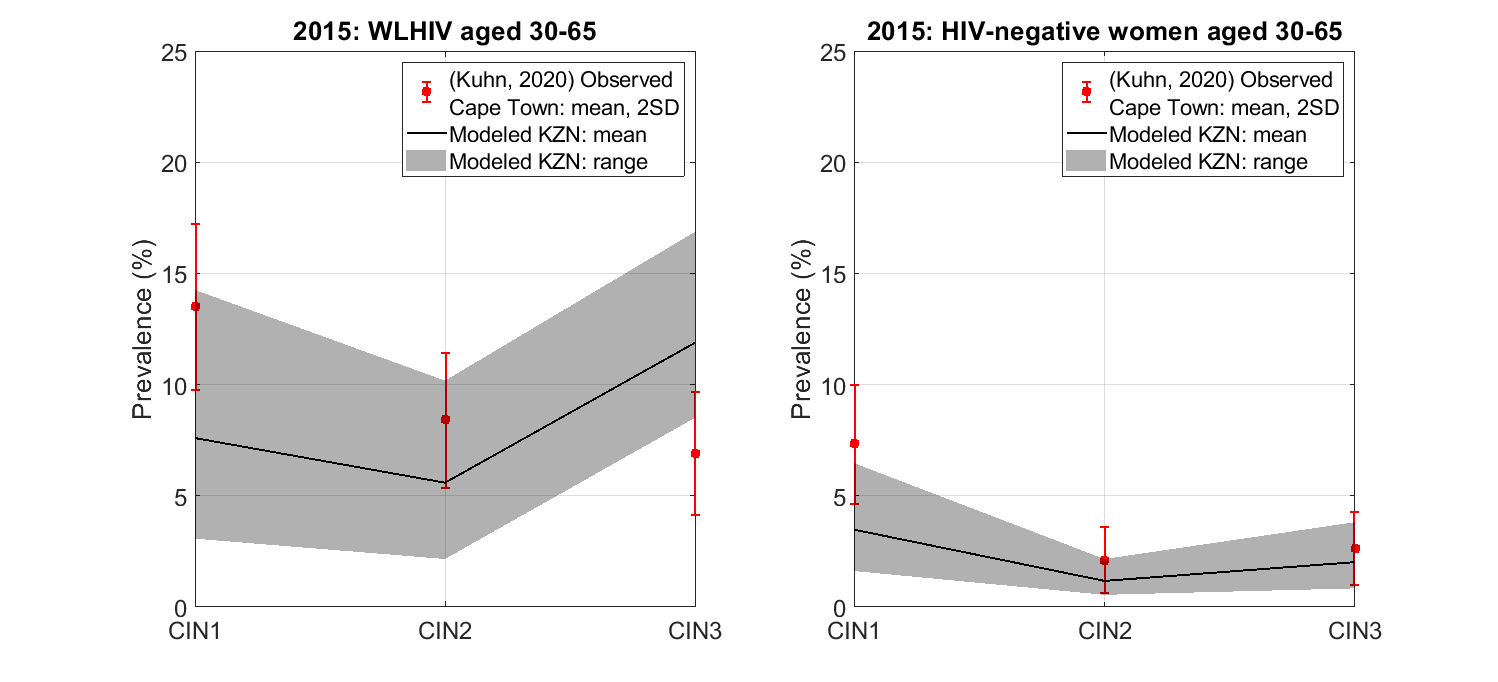
***

**Figure S14. Model fit to observed prevalence data by CIN stage and HIV status in 2015.** Standard deviation of the observed data calculated assuming that prevalence proportions follow a normal approximation of the binomial distribution. Shaded regions represent the range of estimates using the 25 best-fitting model parameter sets.

***
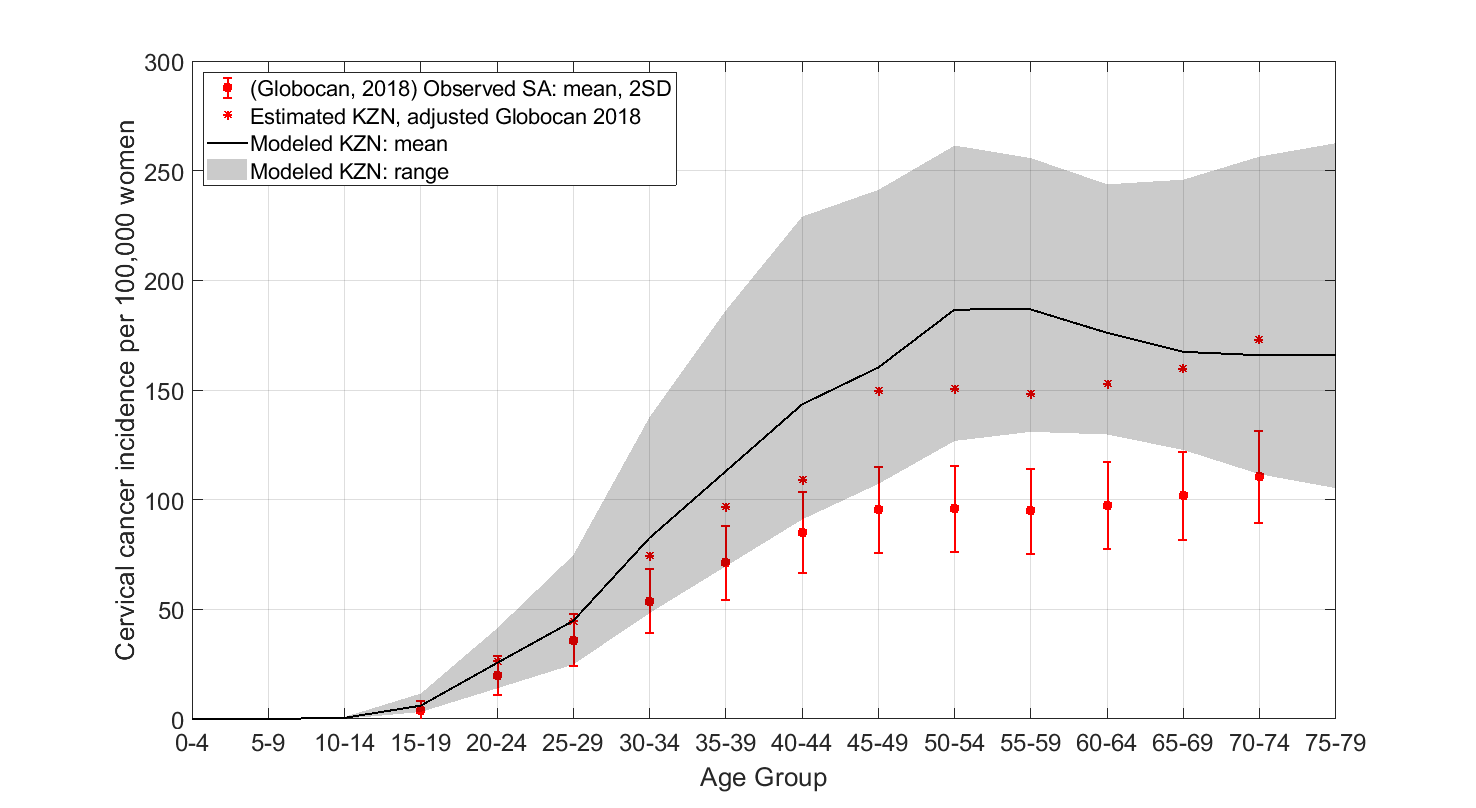
***

**Figure S15. Model fit to observed cervical cancer incidence data by age in 2018.** Asterisks represent South Africa Globocan 2018 rates adjusted to take into account higher HIV prevalence in KwaZulu-Natal. Standard deviation of the observed data calculated assuming that incidence follows a normal approximation of the Poisson distribution. Shaded region represents the range of estimates using the 25 best-fitting model parameter sets.

***
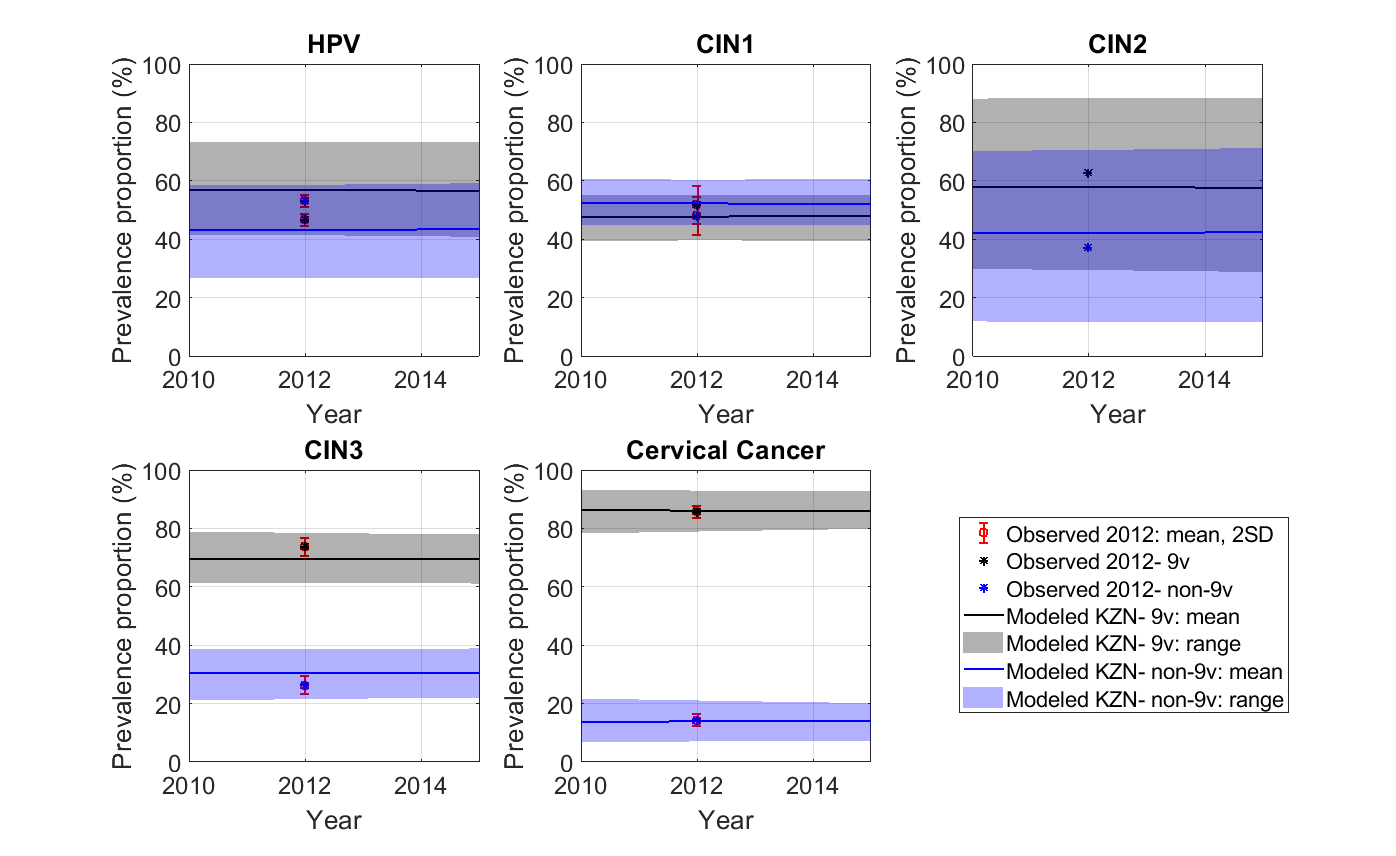
***

**Figure S16. Model fit to observed type distribution data by state in 2012 among all ages.** Vaccine-type (9v) and non-vaccine type (non-9v) HPV coinfections are classified as vaccine-type HPV. The type distribution for CIN2 was not fit to observed data, but is displayed relative to the average of observed CIN1 and CIN3 data. Standard deviation of the observed data calculated assuming that prevalence proportions follow a normal approximation of the binomial distribution. Shaded regions represent the range of estimates using the 25 best-fitting model parameter sets.

### III.a.v. Approximate Bayesian Computation-Sequential Monte Carlo (ABC-SMC) algorithm

The ABC-SMC algorithm repeats the following steps until approximate convergence is achieved^128-130^:

1. Sample the parameter space
2. Simulate model outputs for each potential parameter set
3. Calculate a likelihood for each parameter set
4. Retain parameter sets with the highest likelihood (that produce model outputs most similar to observed data)
5. Weight the retained parameter sets as the inverse of their probability of having been sampled
6. Re-sample from the retained parameter sets according to their weights, and then use a Gaussian perturbation kernel to avoid re-sampling the exact same sets.

The basic idea is to sequentially sample the parameter space, and then use maximum likelihood estimation to identify the parameter sets that best fit the observed data. At each iteration we retain only a proportion (*alpha*) of the best-fitting sets and then re-sample from those sets, therefore focusing sampling in the areas of highest likelihood. By assigning weights to the parameter sets in the re-sampling pool, we ensure thorough exploration of the parameter space. Eventually, the probability density distribution of accepted parameter sets converges on the posterior probability distribution of the parameters. Convergence is achieved when less than a set proportion (*p_acc_min_)* of accepted particles have a likelihood greater than *epsilon,* or the smallest likelihood of the accepted particles from the last iteration. Smaller values of *alpha* and *p_acc_min_* improve the quality of the final posterior approximation but increase the total number of model runs that will be needed. To balance quality with computational time, we choose intermediate values of *alpha* = 0.6 (phase 1) or 0.4 (phase 2), and *p_acc_min_* = 0.1.

### III.a.vi. Likelihood calculation

The model was calibrated to fit observed data on HIV, HPV, and population dynamics from 1996 to 2019 (Tables S24, S30). We defined likelihood equations for each target. By assuming that all outputs followed independent normal distributions, we were able to use the same equation form for all targets. For prevalence data, we assumed a normal approximation of the binomial where $\mu$ = prevalence proportion (*p*) and variance = (*p*(1-*p*))/*N* for a sample size *N*. For incidence data, we assumed a normal approximation of a Poisson distribution where $\mu$= *λ*, variance = *λ*, and *λ* = the annual CC incidence rate. We assumed a normal distribution for the total population size of KwaZulu-Natal and that the 2019 estimate had the same variance as the 2011 estimate. We believe this is conservative as trends indicate that variance will continue to decrease as sampling techniques improve. In most cases, mean values were derived from a single study. However, the proportions of vaccine and non-vaccine type HPV by disease stage were averaged across several studies. Therefore, we assumed a normal approximation of the binomial and calculated the variance of an average as = (1/*N*)^2 * (sum of the individual variances). When observed values were estimated from a multi-year sample, we compared to modeled results from the year at the middle of the time interval.

| **Equation variables** | |
| --- | --- |
| $n$ | The number of outcomes, or calibration targets |
| $\mu$ | Vector of observed outcome means of length $n$ |
| $\sigma^{2}$ | Vector of observed outcome variances of length $n$ |
| $x$ | Vector of model-generated outcome means of length $n$ |

We calculated the likelihood of the $n$ observed outcomes with mean $\mu$ and variance $\sigma^{2}$ given the model-generated set of means $x$ as:

$L\left( \mu,\sigma^{2}|x \right)=\left( 2\pi\sigma^{2} \right)^{\frac{-n}{2}}\cdot exp\left( -\frac{1}{2\sigma^{2}}\sum_{i=1}^{n} \left( x_{i}-\mu\right)^{2} \right)$

However, it is often more convenient to work with the natural logarithm of the likelihood function, or the log-likelihood:

$$ln \left( L\left( \mu,\sigma^{2}|x \right) \right)=-\frac{n}{2}\cdot\ln\left( 2\pi\sigma^{2} \right)-\frac{1}{2\sigma^{2}}\sum_{i=1}^{n} \left( x_{i}-\mu\right)^{2}$$

Since $L\left( \mu,\sigma^{2}|x \right)$ and $ln \left( L\left( \mu,\sigma^{2}|x \right) \right)$ are monotonically related, we maximize $ln \left( L\left( \mu,\sigma^{2}|x \right) \right)$ by searching for $(\mu,\sigma^{2})$ such that:

$$\nabla ln \left( L\left( \mu,\sigma^{2}|x \right) \right)=0$$

## III.b. Validation

To verify our ability to accurately predict future outcomes, we validated our HIV natural history module by comparing model output to additional time points and data sources. More specifically, we compared our model outputs to estimated HIV prevalence at later time points by gender and age from the same reference as our calibration data set ^14^. We also compared our modeled HIV prevalence for both genders combined in broad age groups to observed data for KZN from the 2017 SABSSM survey ^2^. Finally, we compared HIV incidence, a non-calibrated output, to observed data from the Africa Centre cohort in KZN, the same cohort captured by our prevalence data ^143^. Alignment of our model to these external data supports the robustness of our predicted HIV dynamics.

Ideally, we would also validate our HPV and cervical cancer modules to additional data on HPV and/or cervical cancer outcomes. However, data are sparse for these outcomes, and we used the best data available for calibration. Due to the limited reliability of local cancer registry data, GLOBOCAN estimates provided the best measure of cervical cancer incidence for our model. These estimates are updated every few years, but they are not suitable for direct use as a time series given that the methods used for estimation have changed over time. As such, we would be unable to discern whether differences between our model predictions and GLOBOCAN estimates for later years are attributable to the validity of the model or the uncertainty and variability in methods used for estimated incidence.

**Table S32. Validation targets.**

| **Validation: HIV prevalence targets by gender** | | | | | | | |
| --- | --- | --- | --- | --- | --- | --- | --- |
| **Criteria** | **Gender** | **Year** | **Age Group** | **Cases** | **N** | **Mean** | **References** |
| **HIV Prevalence among Men** | **Men** | **2010** | **15-19** | 17 | 1060 | 0.0160 | Africa Centre cohort (now on AHRI) data request ^14^ |
|  |  |  | **20-24** | 52 | 544 | 0.0956 |  |
|  |  |  | **25-29** | 69 | 238 | 0.2899 |  |
|  |  |  | **30-34** | 79 | 170 | 0.4647 |  |
|  |  |  | **35-39** | 77 | 148 | 0.5203 |  |
|  |  |  | **40-44** | 58 | 139 | 0.4173 |  |
|  |  |  | **45-49** | 48 | 131 | 0.3664 |  |
|  | **Men** | **2011** | **15-19** | 17 | 917 | 0.0185 |  |
|  |  |  | **20-24** | 41 | 511 | 0.0802 |  |
|  |  |  | **25-29** | 57 | 260 | 0.2192 |  |
|  |  |  | **30-34** | 77 | 173 | 0.4451 |  |
|  |  |  | **35-39** | 70 | 158 | 0.4430 |  |
|  |  |  | **40-44** | 49 | 118 | 0.4153 |  |
|  |  |  | **45-49** | 49 | 132 | 0.3712 |  |
|  | **Men** | **2012** | **15-19** | 23 | 837 | 0.0275 |  |
|  |  |  | **20-24** | 39 | 395 | 0.0987 |  |
|  |  |  | **25-29** | 52 | 209 | 0.2488 |  |
|  |  |  | **30-34** | 62 | 157 | 0.3949 |  |
|  |  |  | **35-39** | 63 | 127 | 0.4961 |  |
|  |  |  | **40-44** | 50 | 97 | 0.5155 |  |
|  |  |  | **45-49** | 34 | 103 | 0.3301 |  |
| **HIV Prevalence among Men** | **Men** | **2013** | **15-19** | 39 | 1128 | 0.0346 |  |
|  |  |  | **20-24** | 47 | 487 | 0.0965 |  |
|  |  |  | **25-29** | 74 | 248 | 0.2984 |  |
|  |  |  | **30-34** | 85 | 180 | 0.4722 |  |
|  |  |  | **35-39** | 95 | 150 | 0.6333 |  |
|  |  |  | **40-44** | 63 | 122 | 0.5164 |  |
|  |  |  | **45-49** | 46 | 115 | 0.4000 |  |
|  | **Men** | **2014** | **15-19** | 28 | 975 | 0.0287 |  |
|  |  |  | **20-24** | 51 | 430 | 0.1186 |  |
|  |  |  | **25-29** | 80 | 226 | 0.3540 |  |
|  |  |  | **30-34** | 89 | 192 | 0.4635 |  |
|  |  |  | **35-39** | 73 | 142 | 0.5141 |  |
|  |  |  | **40-44** | 79 | 133 | 0.5940 |  |
|  |  |  | **45-49** | 45 | 111 | 0.4054 |  |
|  | **Men** | **2015** | **15-19** | 53 | 1343 | 0.0395 |  |
|  |  |  | **20-24** | 43 | 598 | 0.0719 |  |
|  |  |  | **25-29** | 94 | 340 | 0.2765 |  |
|  |  |  | **30-34** | 122 | 293 | 0.4164 |  |
|  |  |  | **35-39** | 89 | 171 | 0.5205 |  |
|  |  |  | **40-44** | 88 | 167 | 0.5269 |  |
|  |  |  | **45-49** | 69 | 155 | 0.4452 |  |
|  | **Men** | **2016** | **15-19** | 43 | 956 | 0.0450 |  |
|  |  |  | **20-24** | 34 | 424 | 0.0802 |  |
|  |  |  | **25-29** | 65 | 238 | 0.2731 |  |
|  |  |  | **30-34** | 85 | 202 | 0.4208 |  |
|  |  |  | **35-39** | 76 | 148 | 0.5135 |  |
|  |  |  | **40-44** | 65 | 127 | 0.5118 |  |
|  |  |  | **45-49** | 48 | 92 | 0.5217 |  |
| **Criteria** | **Gender** | **Year** | **Age Group** | **Cases** | **N** | **Mean** | **References** |
| **HIV Prevalence among Women** | **Women** | **2010** | **15-19** | 114 | 1227 | 0.0929 | Africa Centre cohort (now on AHRI) data request ^14^ |
|  |  |  | **20-24** | 289 | 920 | 0.3141 |  |
|  |  |  | **25-29** | 375 | 704 | 0.5327 |  |
|  |  |  | **30-34** | 303 | 512 | 0.5918 |  |
|  |  |  | **35-39** | 265 | 491 | 0.5397 |  |
|  |  |  | **40-44** | 187 | 438 | 0.4269 |  |
|  |  |  | **45-49** | 184 | 569 | 0.3234 |  |
|  | **Women** | **2011** | **15-19** | 95 | 1053 | 0.0902 |  |
|  |  |  | **20-24** | 255 | 805 | 0.3168 |  |
|  |  |  | **25-29** | 316 | 611 | 0.5172 |  |
|  |  |  | **30-34** | 281 | 458 | 0.6135 |  |
|  |  |  | **35-39** | 245 | 453 | 0.5408 |  |
|  |  |  | **40-44** | 193 | 446 | 0.4327 |  |
|  |  |  | **45-49** | 166 | 484 | 0.3430 |  |
|  | **Women** | **2012** | **15-19** | 97 | 928 | 0.1045 |  |
|  |  |  | **20-24** | 201 | 656 | 0.3064 |  |
|  |  |  | **25-29** | 253 | 498 | 0.5080 |  |
|  |  |  | **30-34** | 210 | 358 | 0.5866 |  |
|  |  |  | **35-39** | 211 | 359 | 0.5877 |  |
|  |  |  | **40-44** | 154 | 340 | 0.4529 |  |
|  |  |  | **45-49** | 143 | 365 | 0.3918 |  |
| **HIV Prevalence among Women** | **Women** | **2013** | **15-19** | 119 | 1275 | 0.0933 |  |
|  |  |  | **20-24** | 275 | 810 | 0.3395 |  |
|  |  |  | **25-29** | 308 | 600 | 0.5133 |  |
|  |  |  | **30-34** | 331 | 510 | 0.6490 |  |
|  |  |  | **35-39** | 295 | 453 | 0.6512 |  |
|  |  |  | **40-44** | 206 | 419 | 0.4916 |  |
|  |  |  | **45-49** | 175 | 422 | 0.4147 |  |
|  | **Women** | **2014** | **15-19** | 114 | 1099 | 0.1037 |  |
|  |  |  | **20-24** | 264 | 764 | 0.3455 |  |
|  |  |  | **25-29** | 321 | 618 | 0.5194 |  |
|  |  |  | **30-34** | 336 | 537 | 0.6257 |  |
|  |  |  | **35-39** | 299 | 458 | 0.6528 |  |
|  |  |  | **40-44** | 230 | 424 | 0.5425 |  |
|  |  |  | **45-49** | 189 | 392 | 0.4821 |  |
|  | **Women** | **2015** | **15-19** | 171 | 1554 | 0.1100 |  |
|  |  |  | **20-24** | 360 | 1055 | 0.3412 |  |
|  |  |  | **25-29** | 468 | 867 | 0.5398 |  |
|  |  |  | **30-34** | 506 | 782 | 0.6471 |  |
|  |  |  | **35-39** | 397 | 614 | 0.6466 |  |
|  |  |  | **40-44** | 332 | 589 | 0.5637 |  |
|  |  |  | **45-49** | 289 | 583 | 0.4957 |  |
|  | **Women** | **2016** | **15-19** | 107 | 1144 | 0.0935 |  |
|  |  |  | **20-24** | 258 | 772 | 0.3342 |  |
|  |  |  | **25-29** | 348 | 664 | 0.5241 |  |
|  |  |  | **30-34** | 400 | 634 | 0.6309 |  |
|  |  |  | **35-39** | 310 | 463 | 0.6695 |  |
|  |  |  | **40-44** | 258 | 421 | 0.6128 |  |
|  |  |  | **45-49** | 217 | 432 | 0.5023 |  |

| **Validation: HIV prevalence targets, genders combined** | | | |
| --- | --- | --- | --- |
| **Criteria** | **Year** | **Mean [95% CI]** | **References** |
| **HIV Prevalence, ages 15-49** | **2010** | 0.2904 | Africa Centre cohort (now on AHRI) data request ^14^ |
|  | **2011** | 0.2905 |  |
|  | **2012** | 0.2932 |  |
|  | **2013** | 0.3119 |  |
|  | **2014** | 0.3381 |  |
|  | **2015** | 0.3382 |  |
|  | **2016** | 0.3445 |  |
|  | **2002** | 0.1570 [0.1160, 0.2110] | ^2^ |
|  | **2005** | 0.2190 [0.1830, 0.2590] |  |
|  | **2008** | 0.2580 [0.2210, 0.2980] |  |
|  | **2012** | 0.2790 [0.2520, 0.3080] |  |
|  | **2017** | 0.2700 [0.2390, 0.3040] |  |
| **HIV Prevalence, ages 25+** | **2002** | 0.1490 [0.1010, 0.2150] | ^2^ |
|  | **2005** | 0.2050 [0.1680, 0.2460] |  |
|  | **2008** | 0.2350 [0.1970, 0.2780] |  |
|  | **2012** | 0.3010 [0.2690, 0.3360] |  |
|  | **2017** | 0.3120 [0.2760, 0.3500] |  |
| **HIV Prevalence, ages 50+** | **2002** | 0.1100 [0.0450, 0.2430] | ^2^ |
|  | **2005** | 0.0950 [0.0590, 0.1480] |  |
|  | **2008** | 0.0610 [0.0370, 0.1010] |  |
|  | **2012** | 0.0980 [0.0740, 0.1280] |  |
|  | **2017** | 0.1790 [0.1390, 0.2280] |  |

| **Validation: HIV incidence targets by gender** | | | |
| --- | --- | --- | --- |
| **Criteria** | **Year** | **Mean [95% CI]** | **References** |
| **Men aged 15-29, HIV incidence** | **2005-2017** | Values digitized from reference | ^143^ |
| **Men aged 30-54, HIV incidence** | **2005-2017** | Values digitized from reference |  |
| **Men aged 15-54, HIV incidence** | **2005-2017** | Values digitized from reference |  |
| **Women aged 15-29, HIV incidence** | **2005-2017** | Values digitized from reference |  |
| **Women aged 30-49, HIV incidence** | **2005-2017** | Values digitized from reference |  |
| **Women aged 15-49, HIV incidence** | **2005-2017** | Values digitized from reference |  |


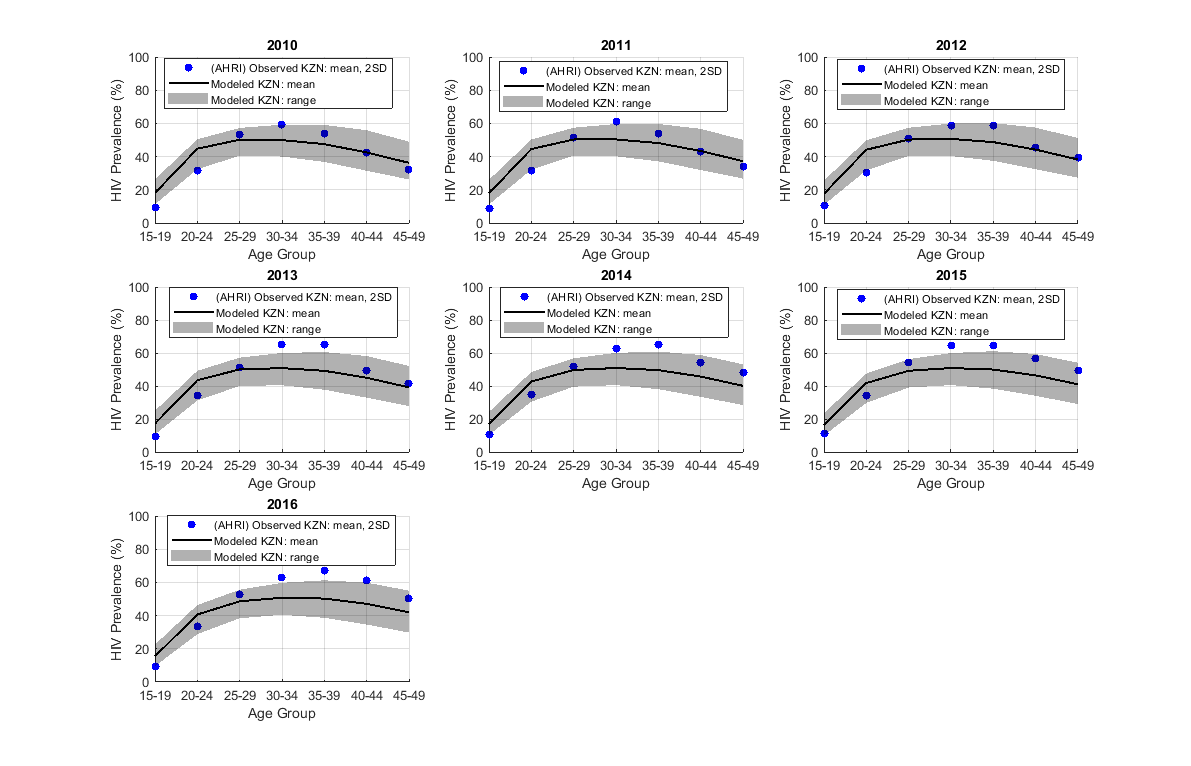


**Figure S17. Model validation to observed HIV prevalence data in women by age over time.** Shaded regions represent the range of estimates using the 25 best-fitting model parameter sets.

 
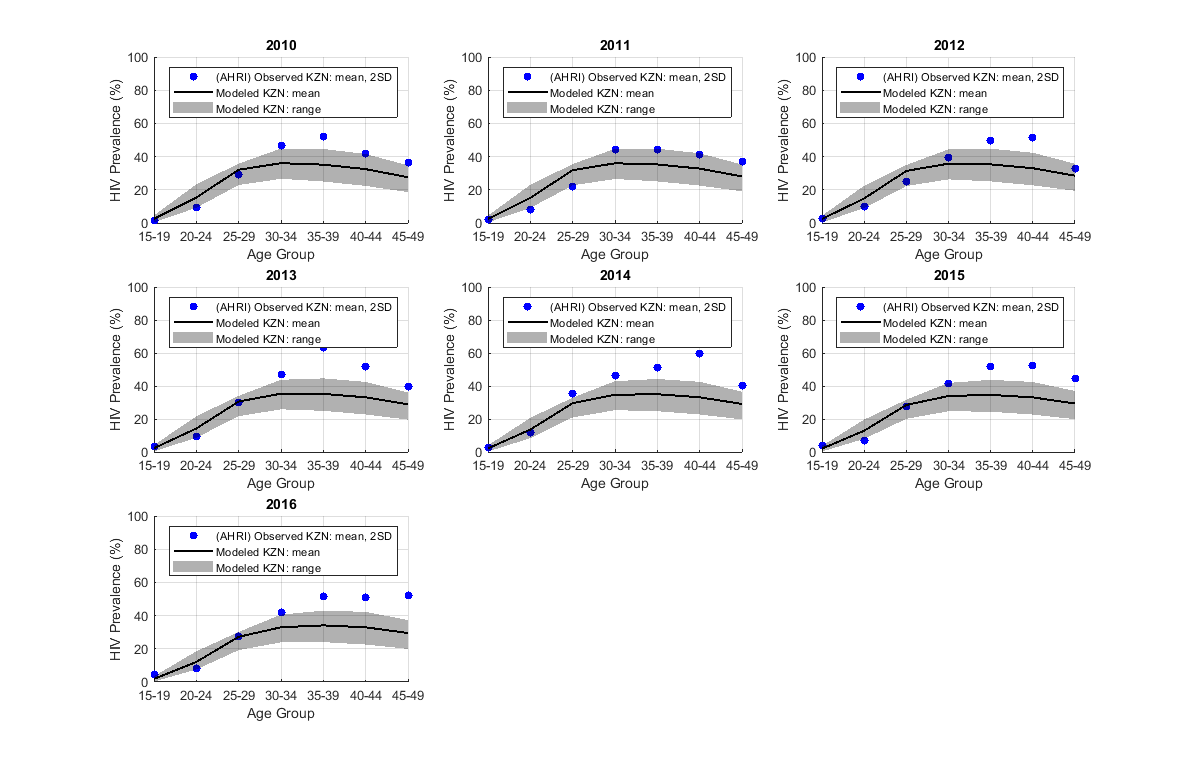


**Figure S18. Model validation to observed HIV prevalence data in men by age over time.** Shaded regions represent the range of estimates using the 25 best-fitting model parameter sets.

**
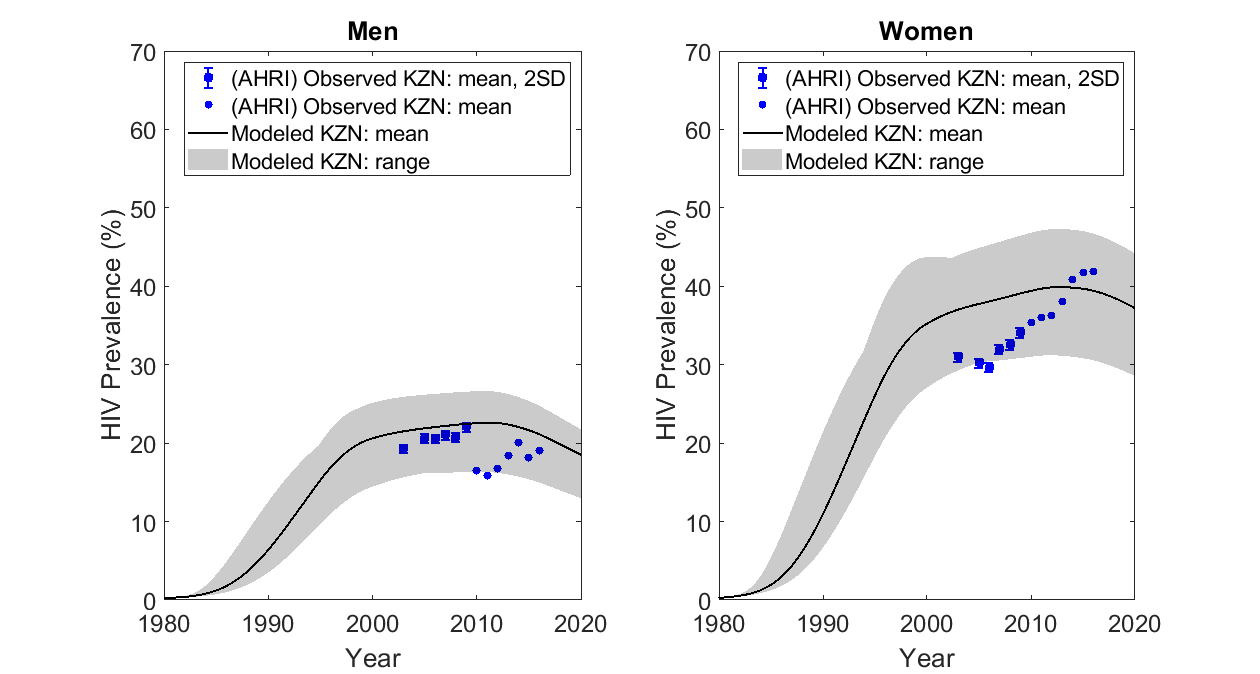
**

**Figure S19. Model validation to observed HIV prevalence data by gender over time.** Blue error bars from 2003-2009 recalculate HIV prevalence for ages 15-49 combined from the calibration dataset, while blue data from 2010-2016 represents later timepoints used only for validation. Shaded regions represent the range of estimates using the 25 best-fitting model parameter sets.


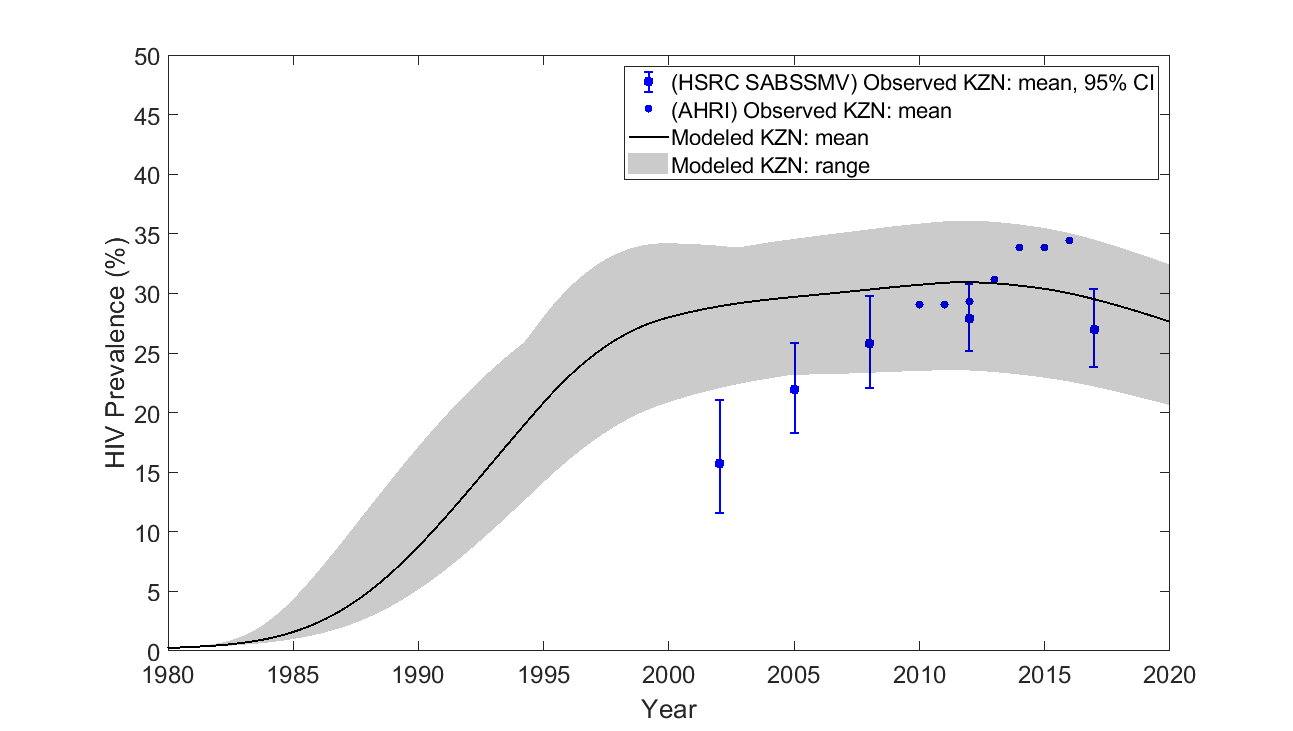


**Figure S20. Model validation to observed HIV prevalence data among ages 15-49 over time.** Shaded region represents the range of estimates using the 25 best-fitting model parameter sets.


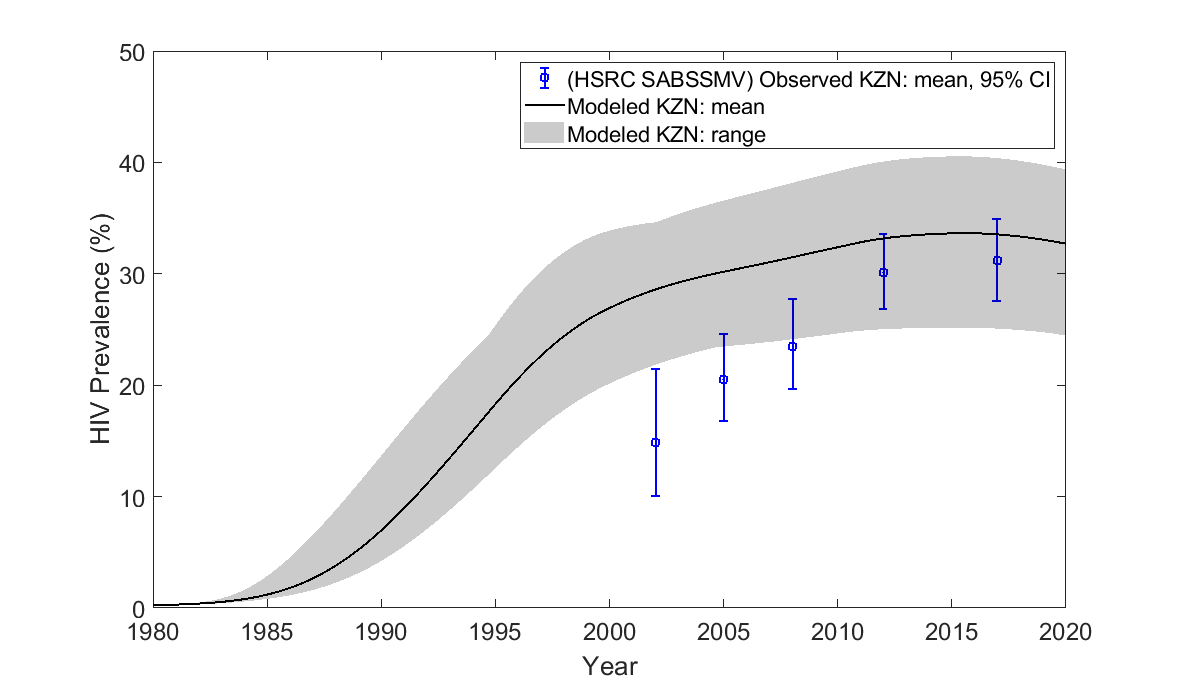


**Figure S21. Model validation to observed HIV prevalence data among ages 25+ over time.** Shaded region represents the range of estimates using the 25 best-fitting model parameter sets.


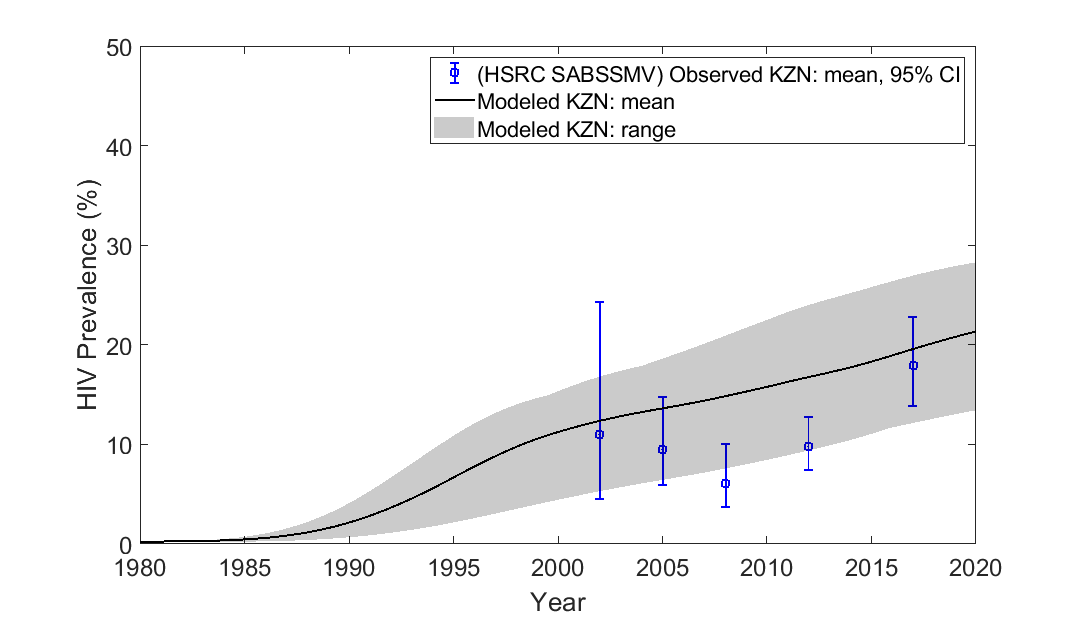


**Figure S22. Model validation to observed HIV prevalence data among ages 50+ over time.** Shaded region represents the range of estimates using the 25 best-fitting model parameter sets.

 
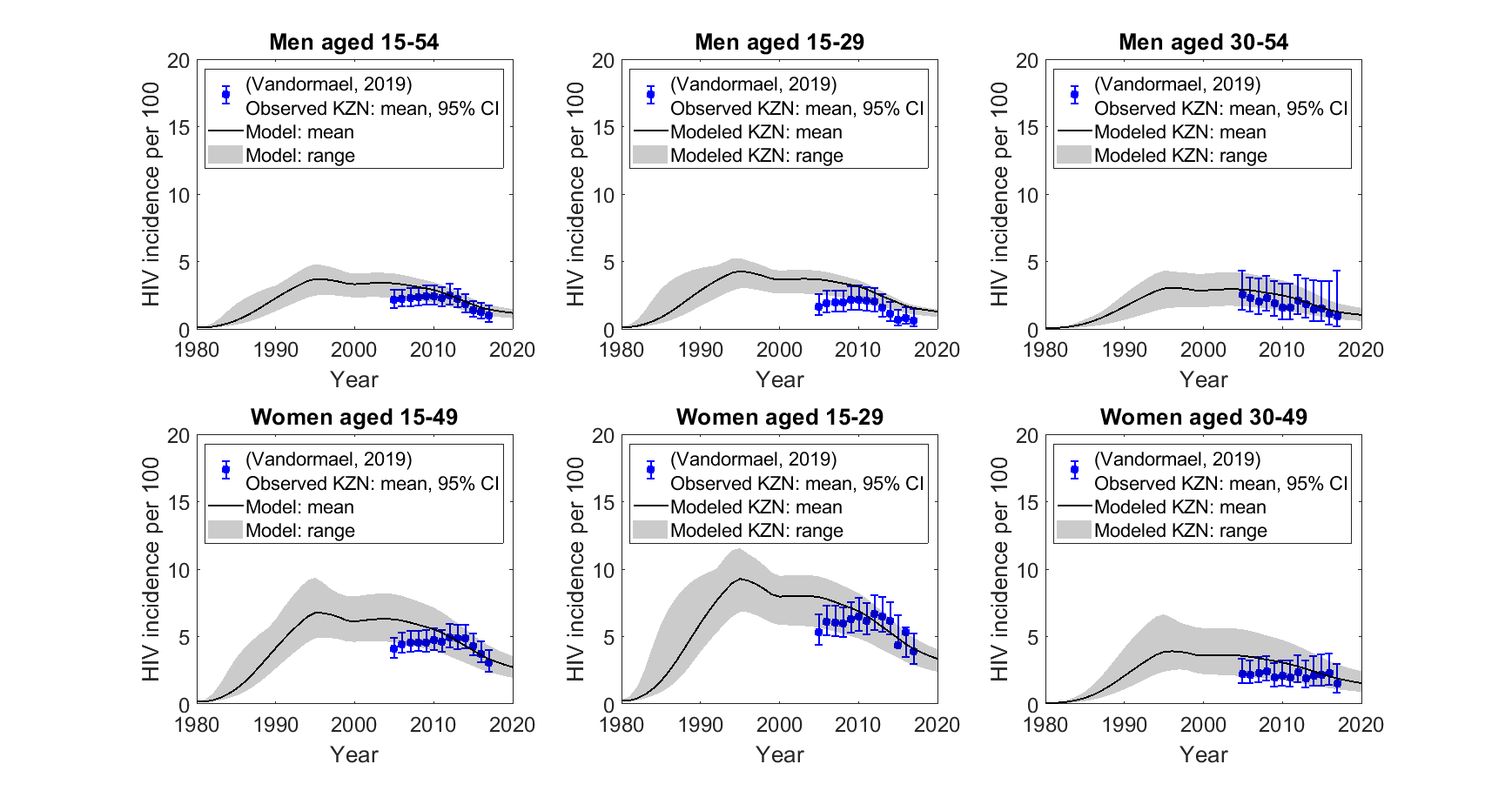


**Figure S23. Model validation to observed HIV incidence data by gender and age over time.** Shaded regions represent the range of estimates using the 25 best-fitting model parameter sets.

# IV. Differential equations

We use a system of differential equations to estimate changes in population and infection dynamics over each time step. We split the full system of equations into topic-based modules and solve each iteratively using a 4^th^-order Runge-Kutta numerical solver in MATLAB. The order is largely historical and based on the work of (Tan et al., 2018)^3^. The modules are:

1. HPV natural history
   1. Progression and clearance of HPV
   2. Progression and regression of precancerous lesions
   3. Development and progression of cervical cancer
   4. Cervical cancer- associated mortality
2. Cervical cancer screening and treatment
   1. Screening
   2. Treatment
3. HPV and HIV transmission
   1. Heterosexual mixing by gender, age, and risk group
   2. Partnership adjustment
   3. HPV infection by type
   4. HIV infection
4. HIV natural history and treatment
   1. CD4 progression
   2. Viral load progression
   3. ART initiation, discontinuation, and scale-up by CD4 count
   4. HIV-associated mortality
5. Demography
   1. Births
   2. Mother-to-child HIV transmission
   3. Aging and risk-group redistribution
   4. Natural deaths
6. Voluntary male medical circumcision
7. HPV vaccination
   1. School-based regimen
   2. Catch-up regimen

Ideally, each module would be completely independent from the others. This may not be the case here, but a comparative analysis revealed no significant difference in the results of split or combined approaches. We therefore chose the split approach for efficiency as it allows us to use a larger time step.

Throughout each simulation, we track population demographics and the number of persons with infection, with progressed disease, or with preventative or therapeutic treatment. We describe these states $X_{g,a,r}^{d,v,h,s,x,p}(t)$ with the following indices (using 1-based indexing):

| **Index** | **Description** | **Values** |
| --- | --- | --- |
| *d* | HIV disease state, CD4 count, circumcision status, and ART status | 1. HIV-negative, uncircumcised 2. HIV-negative, circumcised 3. HIV-positive, acute infection 4. HIV-positive, CD4 > 500 cells/µL 5. HIV-positive, CD4 350-500 cells/µL 6. HIV-positive, CD4 200-350 cells/µL 7. HIV-positive, CD4 ≤ 200 cells/µL 8. HIV-positive, on ART |
| *v* | HIV viral load | 1. If ( 2 ˂ *d* ˂ 8 ), Acute infection; if ( *d* = 1,2 ), HIV-negative: VL = 0.0 2. Asymptomatic: VL = 3.0-4.5 log_10_ 3. Pre-AIDS symptomatic: VL = 4.0-5.5 log_10_ 4. AIDS: VL = 5.5-7.0 log_10_ 5. Late-stage 6. On ART and virally suppressed: VL = 0.0 |
| *h* | Vaccine-type HPV precancer or disease state | 1. Susceptible 2. Infected 3. CIN1 4. CIN2 5. CIN3 6. Cervical cancer or hysterectomy 7. Immune |
| *s* | Non-vaccine type HPV precancer or disease state | 1. Susceptible 2. Infected 3. CIN1 4. CIN2 5. CIN3 6. Cervical cancer or hysterectomy 7. Immune |
| *x* | Cervical cancer or hysterectomy status | 1. If ( *h* = 6 or *s* = 6 ), Cervical cancer, local; else, no cancer or hysterectomy 2. Cervical cancer, regional 3. Cervical cancer, distant 4. Hysterectomy |
| *p* | Vaccination and screening history | 1. Non-vaccinated, non-screened 2. Vaccinated 3. Screened 4. Vaccinated and screened |
| *g* | Gender | 1. Men 2. Women |
| *a* | Age | 1. 0-4 2. 5-9 3. 10-14 4. 15-19 5. 20-24 6. 25-29 7. 30-34 8. 35-39 9. 40-44 10. 45-49 11. 50-54 12. 55-59 13. 60-64 14. 65-69 15. 70-74 16. 75-79 |
| *r* | Risk | 1. Low risk 2. Moderate risk 3. High risk |

## IV.a. Demography

| **Equation variables** | |
| --- | --- |
| $\gamma_{a}^{d}$(t) | The annual fertility rate for women by age *a* and HIV disease stage *d*.  Women aged 15-49 bear children. |
| $\eta\left( t \right)$ | The proportion of births from women living with HIV that result in vertical transmission. |
| $b\_s\left( t \right)$ | Number of births by HIV-negative women and women on ART. |
| $b\_i\left( t \right)$ | Number of births by women living with HIV. |
| $b_{g,a,r}^{d,1,h,s,x,p}(t)$ | Number of infant births of HIV disease stage *d* and gender *g.*  We assume an equal gender ratio at birth of 1:1, that all newborns are born as low risk, no vertical transmission of HPV, and that if HIV is vertically transmitted, that infected newborns are born into the acute stage of HIV. |
| $\phi_{g,a,r}$ | Distribution of sexual risk *r* by gender *g* and age *a.* (Currently, the risk distribution derived from male partner data is used for both men and women for simplicity.) |
| ${\mu\_bkrd}_{g,a}(t)$ | Annual background mortality rate by gender *g* and age *a.* |

### Fertility

The number of births by HIV status of the mother are calculated as:

*HIV-negative women and women on ART*

$$b\_s\left( t \right)=\sum_{h=1}^{7} \sum_{s=1}^{7} \sum_{x=1}^{3} \sum_{p=1}^{4} \sum_{a=4}^{10} \sum_{r=1}^{3} \left[ \gamma_{a}^{1}\left( t \right)\cdot X_{g,a,r}^{1,1,h,s,x,p}\left( t \right)+\gamma_{a}^{8}\left( t \right)\cdot X_{g,a,r}^{8,6,h,s,x,p}\left( t \right) \right]$$

*Women living with HIV*

$$b\_i\left( t \right)=\sum_{d=3}^{7} \sum_{v=1}^{5} \sum_{h=1}^{7} \sum_{s=1}^{7} \sum_{x=1}^{3} \sum_{p=1}^{4} \sum_{a=4}^{10} \sum_{r=1}^{3} \left[ \gamma_{a}^{d}\left( t \right)\cdot X_{g,a,r}^{d,v,h,s,x,p}(t) \right]$$

We then compute the number of births by gender and HIV status of the infant as:

*HIV-negative, uncircumcised births*

*For h = s = x = p = a = r = 1,*

$$b_{g,a,r}^{1,1,h,s,x,p}\left( t \right)=0.5 \left( b\_s\left( t \right)+\left( 1-\eta\left( t \right) \right)b\_i\left( t \right) \right)$$

else,

$$b_{g,a,r}^{1,1,h,s,x,p}\left( t \right)=0$$

*HIV-positive births*

*For h = s = x = p = a = r = 1,*

$$b_{g,a,r}^{3,1,h,s,x,p}\left( t \right)=0.5 \eta\left( t \right)\cdot b\_i(t)$$

else,

$$b_{g,a,r}^{3,1,h,s,x,p}\left( t \right)=0$$

### Aging

To age the population, one-fifth of each compartment moves to the next age group while maintaining the same gender, disease state, and sexual risk distribution $\phi_{g,a,r}$:

$$\frac{dX_{g,1,r}^{d,v,h,s,x,p}(t)}{dt}=-\frac{1}{5}\sum_{r=1}^{3} X_{g,1,r}^{d,v,h,s,x,p}\left( t \right) \cdot\phi_{g,a,r} (for a=1)$$

$$\frac{dX_{g,a,r}^{d,v,h,s,x,p}(t)}{dt}=-\frac{1}{5}\sum_{r=1}^{3} X_{g,a,r}^{d,v,h,s,x,p}\left( t \right) \cdot\phi_{g,a,r}+\frac{1}{5}\sum_{r=1}^{3} X_{g,a-1,r}^{d,v,h,s,x,p}\left( t \right) \cdot\phi_{g,a-1,r} (for a\neq1)$$

Upon aging to the next five-year group, individuals are re-distributed into the closest unfilled risk group to match observed data on the age distribution of low, moderate, and high-risk individuals.

### Mortality

We compute the number of deaths due to background mortality as:

$$\frac{dX_{g,a,r}^{d,v,h,s,x,p}(t)}{dt}=-{\mu\_bkrd}_{g,a}\left( t \right)\cdot X_{g,a,r}^{d,v,h,s,x,p}\left( t \right)$$

## IV.b. Sexual Behavior

### Mixing matrix

| **Equation variables** | |
| --- | --- |
| $c_{g,a,r}$ | Number of partners a person has per year of gender *g,* age *a,* and sexual-risk group *r* (ie. the partner exchange rate, or contact rate). |
| $\epsilon_{a}$ | Mixing parameter by age *a.*  We assume a mixing pattern that is partially random and partially off-diagonal (0 < $\epsilon_{a}$ < 1), where ($\epsilon_{a}=0)$ indicates completely off-diagonal mixing, and ($\epsilon_{a}=1)$ indicates completely random mixing. |
| $\epsilon_{r}$ | Mixing parameter by sexual-risk group *r.*  We assume a mixing pattern that is partially random and partially on-diagonal (0 < $\epsilon_{r}$ < 1), where ($\epsilon_{r}=0)$ indicates completely on-diagonal mixing, and ($\epsilon_{r}=1)$ indicates completely random mixing. |
| $\delta_{g,a,a^{'}}$ | Mixing pattern by age.  In completely non-random mixing by age, women are most likely to form partnerships with men of the next oldest age group*.* We represent this pattern using on off-diagonal matrix.  For men (*g* = 1) of age *a* mixing with women of age *a’*:  = 0.3 if ($a=a^{'}$)  = 0.7 if ($a=a^{'}+1$)  except for the following (correct for no sexual activity before age group 3):  = 0.0 if ($a=a^{'}=1$)  = 0.0 if ($a=$ 2) and (*a’* = 1)  = 0.0 if ($a=$2) and (*a’* = 2)  = 0.0 if ($a=$ 3) and (*a’* = 2)  For women (*g* = 2) of age *a* mixing with men of age *a’*:  = 0.3 if ($a=a^{'}$)  = 0.7 if ($a=a^{'}-1$)  except for the following (correct for no sexual activity before age group 3):  = 0.0 if ($a=a^{'}=$1)  = 0.0 if ($a=$1) and (*a’* = 2)  = 0.0 if ($a=a^{'}=$2)  = 0.0 if ($a=$2) and (*a’* = 3) |
| $\delta_{r,r^{'}}$ | Mixing pattern by risk.  Completely non-random mixing by risk confines sexual encounters to individuals within the same risk group. We represent this pattern using an identity matrix.  = 1.0 if ($r=r^{'}$)  = 0.0 if ($r\neq r^{'}$) |

For a person of gender *g,* age *a,* and sexual-risk group *r,* we use the mixing matrix $\rho_{g,a,a^{'},r,r^{'}}(t)$ to describe the proportion of sexual partners that come from age group *a’* and sexual-risk group *r’*. We assume that mixing is partially random and partially designated by a mixing pattern $\delta_{g,a,a^{'}}$ or $\delta_{r,r^{'}}$. The overall mixing matrix is therefore a weighted average of random mixing proportional to the number of available partnerships of each group, and mixing among groups with similar characteristics. Although an off-diagonal mixing pattern results in the first and last ages groups (ages 10-14 and 75-79) having fewer than 100% of their partnerships, these age groups have relatively few partnerships and contribute marginally to overall infection transmission.

$$\rho_{g,a,a^{'},r,r^{'}}\left( t \right)=\left( \epsilon_{a}\cdot\frac{\sum_{r^{'}=1}^{3} \left( c_{g^{'},a^{'},r^{'}}\cdot\sum_{d^{'}=1}^{8} \sum_{v^{'}=1}^{6} \sum_{h^{'}=1}^{7} \sum_{s^{'}=1}^{7} \sum_{x^{'}=1}^{4} \sum_{p^{'}=1}^{4} X_{g',a',r'}^{d',v',h',s',x',p'}\left( t \right) \right)}{\sum_{a^{'}=1}^{16} \sum_{r^{'}=1}^{3} \left( c_{g^{'},a^{'},r^{'}}\cdot\sum_{d^{'}=1}^{8} \sum_{v^{'}=1}^{6} \sum_{h^{'}=1}^{7} \sum_{s^{'}=1}^{7} \sum_{x^{'}=1}^{4} \sum_{p^{'}=1}^{4} X_{g',a',r'}^{d',v',h',s',x',p'}\left( t \right) \right)}+\left( 1-\epsilon_{a} \right)\delta_{g,a,a^{'}} \right)\cdot\left( \epsilon_{r}\cdot\frac{\left( c_{g^{'},a^{'},r^{'}}\cdot\sum_{d^{'}=1}^{8} \sum_{v^{'}=1}^{6} \sum_{h^{'}=1}^{7} \sum_{s^{'}=1}^{7} \sum_{x^{'}=1}^{4} \sum_{p^{'}=1}^{4} X_{g',a',r'}^{d',v',h',s',x',p'}\left( t \right) \right)}{\sum_{r^{'}=1}^{3} \left( c_{g^{'},a^{'},r^{'}}\cdot\sum_{d^{'}=1}^{8} \sum_{v^{'}=1}^{6} \sum_{h^{'}=1}^{7} \sum_{s^{'}=1}^{7} \sum_{x^{'}=1}^{4} \sum_{p^{'}=1}^{4} X_{g',a',r'}^{d',v',h',s',x',p'}\left( t \right) \right)}+\left( 1-\epsilon_{r} \right)\delta_{g,r,r^{'}} \right)$$

### Rate of partner change

| **Equation variables** | |
| --- | --- |
| $c_{g,a,r}$ | Number of partners a person has per year of gender *g,* age *a,* and sexual-risk group *r* (i.e., the partner exchange rate, or contact rate).  We assume zero partnerships for individuals below the age of sexual debut (age 10). |
| $\theta$ | Gender influence on contact rate adjustment.  We assume an adjusted contact rate equally driven by rates reported by men and women ($\theta$= 0.5), where ($\theta$ = 0) when completely female-driven, and ($\theta$ = 1) when completely male-driven. |
| $\rho_{g,a,a^{'},r,r^{'}}\left( t \right)$ | Mixing matrix for a person of gender *g,* age *a,* and sexual-risk group *r* that describes the proportion of sexual partners that come from age group *a’* and sexual-risk group *r’.*  We assume a solely heterosexual population and therefore that all contacts are with the opposite gender. |

Bias in observed data leads to contact rates $c_{g,a,r}$ that, when assuming solely heterosexual contact, are inconsistent between men and women. We account for this variability by using an adjusted contact rate $c_{g,a,a^{'},r,r^{'}}^{*}(t)$ that ensures that the number of partnerships of men of age *a* and risk group *r* with women of age *a’* and risk group *r’* equals the number of partnerships of women of age *a* and risk group *r* with men of age *a’* and risk group *r’*.

We first calculate the discrepancy between reported contacts among men and women as:

$$B_{a,a^{'},r,r^{'}}\left( t \right)=\frac{c_{1,a,r}\cdot\rho_{1,a,a^{'},r,r^{'}}\left( t \right)\cdot\sum_{d^{'}=1}^{8} \sum_{v^{'}=1}^{6} \sum_{h^{'}=1}^{7} \sum_{s^{'}=1}^{7} \sum_{x^{'}=1}^{4} \sum_{p^{'}=1}^{4} X_{1,a',r'}^{d',v',h',s',x',p'}\left( t \right)}{c_{2,a,r}\cdot\rho_{2,a,a^{'},r,r^{'}}\left( t \right)\cdot\sum_{d=1}^{8} \sum_{v=1}^{6} \sum_{h=1}^{7} \sum_{s=1}^{7} \sum_{x=1}^{4} \sum_{p=1}^{4} X_{2,a,r}^{d,v,h,s,x,p}\left( t \right)}$$

We then compute the adjusted contact rate for women as:

$$c_{2,a,a^{'},r,r^{'}}^{*}\left( t \right)=c_{2,a,r}\cdot\rho_{2,a,a^{'},r,r^{'}}\left( t \right)\cdot{B_{a,a^{'},r,r^{'}}\left( t \right)}^{\theta}\cdot\left( \frac{\sum_{d^{'}=1}^{8} \sum_{v^{'}=1}^{6} \sum_{h^{'}=1}^{7} \sum_{s^{'}=1}^{7} \sum_{x^{'}=1}^{4} \sum_{p^{'}=1}^{4} X_{1,a',r'}^{d',v',h',s',x',p'}\left( t \right)}{\sum_{d=1}^{8} \sum_{v=1}^{6} \sum_{h=1}^{7} \sum_{s=1}^{7} \sum_{x=1}^{4} \sum_{p=1}^{4} X_{2,a,r}^{d,v,h,s,x,p}\left( t \right)} \right)^{-\left( 1-\theta\right)}$$

and for men, an adjusted contact rate of:

$$c_{1,a,a^{'},r,r^{'}}^{*}(t)=c_{1,a,r}\cdot\rho_{1,a,a^{'},r,r^{'}}\left( t \right)\cdot{B_{a,a^{'},r,r^{'}}\left( t \right)}^{-\left( 1-\theta\right)}\cdot\left( \frac{\sum_{d^{'}=1}^{8} \sum_{v^{'}=1}^{6} \sum_{h^{'}=1}^{7} \sum_{s^{'}=1}^{7} \sum_{x^{'}=1}^{4} \sum_{p^{'}=1}^{4} X_{1,a',r'}^{d',v',h',s',x',p'}\left( t \right)}{\sum_{d=1}^{8} \sum_{v=1}^{6} \sum_{h=1}^{7} \sum_{s=1}^{7} \sum_{x=1}^{4} \sum_{p=1}^{4} X_{2,a,r}^{d,v,h,s,x,p}\left( t \right)} \right)^{\theta}$$

## IV.c. Transmission Probabilities

### Per-partnership probability of transmission

| **Equation variables** | |
| --- | --- |
| $A_{g,a,r}$ | Number of acts a person has per partnership of gender *g,* age *a,* and sexual-risk group *r*.  We assume zero acts for individuals below the age of sexual debut (age 10). |
| ${\chi\_HIV}_{g}^{v^{'},x^{'}}$ | Per-act probability of HIV transmission to a person of gender *g* based on the viral load *v’* of the partner living with HIV*.*  We assume the probability of female-to-male HIV transmission is equal to the probability of male-to-female transmission across all viral load stages (${\chi\_HIV}_{1}^{v^{'}}= {\chi\_HIV}_{2}^{v^{'}}$). We reduce HIV per-act transmission as a proxy for decreased sexual activity during late-stage HIV (*v’* = 5), regional or distant cervical cancer (*x’* = 2 or *x’* = 3), or hysterectomy (*x’* = 4). |
| ${\chi\_HPV}_{g}^{v^{'},x^{'}}$ | Per-act probability of HPV transmission to a person of gender *g* by an HPV-positive partner.  We assume the per-act probability of HPV transmission is the same for vaccine-type and non-vaccine-type HPV and across all stages of pre-cancer or cervical cancer. We reduce HPV per-act transmission as a proxy for decreased sexual activity during late-stage HIV (*v’* = 5) or regional or distant cervical cancer (*x’* = 2 or *x’* = 3). We assume no HPV transmission after hysterectomy (*x’* = 4). |

The per-partnership probability of HIV transmission ${\beta\_HIV}_{g,a,r}^{v^{'},x^{'}}$ is the cumulative risk of acquiring HIV from all sexual acts with a partner. This quantity depends on the per-act probability of HIV transmission and the number of acts per partnership.

We calculate the per-partnership probability of HIV transmission to a male partner:

$${\beta\_HIV}_{1,a,r}^{v^{'},x^{'}}=1-{(1-{\chi\_HIV}_{1}^{v^{'},x^{'}})}^{A_{1,a,r}}$$

Similarly, the per-partnership probability of HIV transmission to a female partner:

$${\beta\_HIV}_{2,a,r}^{v^{'},x^{'}}=1-{(1-{\chi\_HIV}_{2}^{v^{'},x^{'}})}^{A_{2,a,r}}$$

Likewise, the per-partnership probability of HPV transmission ${\beta\_HPV}_{g,a,r}^{v^{'},x^{'}}$ depends on the per-act probability of HPV transmission and the number of acts per partnership.

We calculate the per-partnership probability of HPV transmission to a male partner:

$${\beta\_HPV}_{1,a,r}^{v^{'},x^{'}}=1-{(1-{\chi\_HPV}_{1}^{v^{'},x^{'}})}^{A_{1,a,r}}$$

Similarly, the per-partnership probability of HPV transmission to a female partner:

$${\beta\_HPV}_{2,a,r}^{v^{'},x^{'}}=1-{(1-{\chi\_HPV}_{2}^{v^{'},x^{'}})}^{A_{2,a,r}}$$

### Force of infection

| **Equation variables** | |
| --- | --- |
| $c_{g,a,a^{'},r,r^{'}}^{*}(t)$ | Adjusted yearly contact rate for persons of gender *g,* age *a,* and risk group *r,* with persons of the opposite gender, age *a’,* and risk group *r’.* |
| ${\beta\_HIV}_{g,a,r}^{v^{'},x^{'}}$ | Annual per-partnership probability of HIV transmission from a person with HIV with viral load *v’* and cervical cancer stage *x’* to a HIV-susceptible partner with gender *g,* age *a,* and risk group *r.* |
| ${\beta\_HPV}_{g,a,r}^{v^{'},x^{'}}$ | Annual per-partnership probability of HPV transmission from a HPV-infected person with viral load *v’* and cervical cancer stage *x’* to a HPV-susceptible partner with gender *g,* age *a,* and risk group *r.* |

The force of infection represents the cumulative risk of acquiring HIV or HPV from all possible partners, and depends on the adjusted contact rate, the per-partnership probability of transmission, and the proportion of sexually active persons who are HIV- or HPV-infected.

$${\lambda\_HIV}_{g,a,r}(t)=\sum_{a^{'}=1}^{16} \sum_{r^{'}=1}^{3} \left( c_{g,a,a^{'},r,r^{'}}^{*}(t)\cdot\frac{-\sum_{v^{'}=1}^{6} \sum_{x^{'}=1}^{4} ln(1-{\beta\_HIV}_{g,a,r}^{v^{'},x^{'}})\cdot\sum_{d^{'}=3}^{8} \sum_{h^{'}=1}^{7} \sum_{s^{'}=1}^{7} \sum_{p^{'}=1}^{4} X_{g',a',r'}^{d',v',h',s',x',p'}\left( t \right)}{\sum_{d^{'}=1}^{8} \sum_{v^{'}=1}^{6} \sum_{h^{'}=1}^{7} \sum_{s^{'}=1}^{7} \sum_{x^{'}=1}^{4} \sum_{p^{'}=1}^{4} X_{g',a',r'}^{d',v',h',s',x',p'}\left( t \right)} \right)$$

Similarly, the force of infection ${\lambda\_vHPV}_{g,a,r}(t)$ determines vaccine-type HPV transmission:

$${\lambda\_vHPV}_{g,a,r}(t)=\sum_{a^{'}=1}^{16} \sum_{r^{'}=1}^{3} \left( c_{g,a,a^{'},r,r^{'}}^{*}(t)\cdot\frac{-\sum_{v^{'}=1}^{6} \sum_{x^{'}=1}^{4} ln(1-{\beta\_HPV}_{g,a,r}^{v^{'},x^{'}})\cdot\sum_{d^{'}=1}^{8} \sum_{h^{'}=2}^{6} \sum_{s^{'}=1}^{7} \sum_{p^{'}=1}^{4} X_{g',a',r'}^{d',v',h',s',x',p'}\left( t \right)}{\sum_{d^{'}=1}^{8} \sum_{v^{'}=1}^{6} \sum_{h^{'}=1}^{7} \sum_{s^{'}=1}^{7} \sum_{x^{'}=1}^{4} \sum_{p^{'}=1}^{4} X_{g',a',r'}^{d',v',h',s',x',p'}\left( t \right)} \right)$$

and $\lambda{\_nvHPV}_{g,a,r}(t)$ defines non-vaccine-type HPV transmission:

$$\lambda{\_nvHPV}_{g,a,r}\left( t \right)=\sum_{a^{'}=1}^{16} \sum_{r^{'}=1}^{3} \left( c_{g,a,a^{'},r,r^{'}}^{*}(t)\cdot\frac{-\sum_{v^{'}=1}^{6} \sum_{x^{'}=1}^{4} ln(1-{\beta\_HPV}_{g,a,r}^{v^{'},x^{'}})\cdot\sum_{d^{'}=1}^{8} \sum_{h^{'}=1}^{7} \sum_{s^{'}=2}^{6} \sum_{p^{'}=1}^{4} X_{g',a',r'}^{d',v',h',s',x',p'}\left( t \right)}{\sum_{d^{'}=1}^{8} \sum_{v^{'}=1}^{6} \sum_{h^{'}=1}^{7} \sum_{s^{'}=1}^{7} \sum_{x^{'}=1}^{4} \sum_{p^{'}=1}^{4} X_{g',a',r'}^{d',v',h',s',x',p'}\left( t \right)} \right)$$

## IV.d. Natural History and Interventions

### HIV

| **Equation variables** | |
| --- | --- |
| ${\mu\_HIV}_{g,a}^{d}$ | Annual HIV-associated mortality rate by gender *g*, age *a*, and HIV disease stage *d* for (3 ≤ *d* ≤ 8)*.* |
| ${\lambda\_HIV}_{g,a,r}(t)$ | Force of HIV infection for HIV-negative persons by gender *g,* age *a,* and risk *r*. |
| ${\rho\_HIV}_{g}$ | Reduction in HIV acquisition due to circumcision by gender.  Only men receive circumcision (${\rho\_HIV}_{2}=1$). |
| ${\psi\_HIV}_{g}$ | Reduction in HIV acquisition due to population-level condom use by gender. |
| $\omega^{d}$ | The rate of progressing from HIV stage *d* to stage *d + 1,* for (3 ≤ *d* ≤ 7)*.* |
| $l^{d}$ | The rate of progressing from viral load stage *v* to *v + 1,* for (1 ≤ *v* ≤ 5). |
| $P_{g,a}(t)$ | The proportion of HIV-negative persons of gender *g* and age *a* that are circumcised.  Only men receive circumcision ($P_{2,a}(t)$ = 0). |
| $A_{g,a}^{d}(t)$ | The proportion of persons living with HIV of disease stage *d,* gender *g,* and age *a* that initiate ART. |
| $\sigma_{g,a,r}^{d,v}(t)$ | The proportion of persons who discontinue ART based on the recent distribution of persons initiating ART by gender *g*, age *a,* risk *r,* disease *d,* and viral *v*. |

We calculate changes in HIV status and HIV stage defined by CD4 count, viral load, and treatment status. The HIV-negative population can acquire HIV after sexual debut with a force of infection reduced by circumcision among men and condom use by either gender. We only track circumcision among HIV-negative men. Individuals with HIV infection experience HIV-associated mortality, CD4 and viral load stage progression, and ART initiation and discontinuation. CD4 and viral load stage are not tracked among persons on treatment.

$$\frac{dX_{g,a,r}^{1,1,h,s,x,p}\left( t \right)}{dt}=-\left( {\psi\_HIV}_{g}{\cdot\lambda\_HIV}_{g,a,r}\left( t \right) +P_{g,a}\left( t \right) \right)X_{g,a,r}^{1,1,h,s,x,p}\left( t \right)$$

*HIV-negative, circumcised*

$$\frac{dX_{g,a,r}^{2,1,h,s,x,p}\left( t \right)}{dt}=P_{g,a}\left( t \right) \cdot X_{g,a,r}^{1,1,h,s,x,p}\left( t \right)$$

$$- \left( {\psi\_HIV}_{g}\cdot{\rho\_HIV}_{g}\cdot{\lambda\_HIV}_{g,a,r}(t) \right) X_{g,a,r}^{2,1,h,s,x,p}\left( t \right)$$

*HIV-positive, acute infection*

$$\frac{dX_{g,a,r}^{3,1,h,s,x,p}\left( t \right)}{dt}={\psi\_HIV}_{g}{\cdot\lambda\_HIV}_{g,a,r}(t)\cdot X_{g,a,r}^{1,1,h,s,x,p}\left( t \right)$$

$$+ {\psi\_HIV}_{g}\cdot{\rho\_HIV}_{g}\cdot{\lambda\_HIV}_{g,a,r}\left( t \right)\cdot X_{g,a,r}^{2,1,h,s,x,p}\left( t \right)+\sigma_{g,a,r}^{3,1}(t){\cdot X}_{g,a,r}^{8,6,h,s,x,p}\left( t \right) -\left( {\mu\_HIV}_{g,a}^{3}+\omega^{3}+A_{g,a}^{3}(t) \right)X_{g,a,r}^{3,1,h,s,x,p}\left( t \right)$$

*HIV-positive, CD4 > 500 cells/µL*

$$\frac{dX_{g,a,r}^{4,v,h,s,x,p}\left( t \right)}{dt}= \omega^{3}{\cdot X}_{g,a,r}^{3,v,h,s,x,p}\left( t \right)+ l^{v-1}{\cdot X}_{g,a,r}^{4,v-1,h,s,x,p}\left( t \right) +\sigma_{g,a,r}^{4,v}(t)\cdot X_{g,a,r}^{8,6,h,s,x,p}\left( t \right)-\left( {\mu\_HIV}_{g,a}^{4}+\omega^{4}+l^{v}+A_{g,a}^{4}(t) \right)X_{g,a,r}^{4,v,h,s,x,p}\left( t \right)$$

*HIV-positive, CD4 350-500 cells/µL*

$$\frac{dX_{g,a,r}^{5,v,h,s,x,p}\left( t \right)}{dt}= \omega^{4}\cdot X_{g,a,r}^{4,v,h,s,x,p}\left( t \right)+ l^{v-1}\cdot X_{g,a,r}^{5,v-1,h,s,x,p}\left( t \right) +\sigma_{g,a,r}^{5,v}(t){\cdot X}_{g,a,r}^{8,6,h,s,x,p}\left( t \right)-\left( {\mu\_HIV}_{g,a}^{5}+\omega^{5}+l^{v}+A_{g,a}^{5}(t) \right)X_{g,a,r}^{5,v,h,s,x,p}\left( t \right)$$

*HIV-positive, CD4 200-350 cells/µL*

$$\frac{dX_{g,a,r}^{6,v,h,s,x,p}\left( t \right)}{dt}= \omega^{5}{\cdot X}_{g,a,r}^{5,v,h,s,x,p}\left( t \right)+ l^{v-1}\cdot X_{g,a,r}^{6,v-1,h,s,x,p}\left( t \right) +\sigma_{g,a,r}^{6,v}(t){\cdot X}_{g,a,r}^{8,6,h,s,x,p}\left( t \right)-\left( {\mu\_HIV}_{g,a}^{6}+\omega^{6}+l^{v}+A_{g,a}^{6}(t) \right)X_{g,a,r}^{6,v,h,s,x,p}\left( t \right)$$

*HIV-positive, CD4 ≤ 200 cells/µL*

$$\frac{dX_{g,a,r}^{7,v,h,s,x,p}\left( t \right)}{dt}= \omega^{6}{\cdot X}_{g,a,r}^{6,v,h,s,x,p}\left( t \right)+ l^{v-1}{\cdot X}_{g,a,r}^{7,v-1,h,s,x,p}\left( t \right) +\sigma_{g,a,r}^{7,v}(t)\cdot X_{g,a,r}^{8,6,h,s,x,p}\left( t \right)-\left( {\mu\_HIV}_{g,a}^{7}+\omega^{7}+l^{v}+A_{g,a}^{7}(t) \right)X_{g,a,r}^{7,v,h,s,x,p}\left( t \right)$$

*HIV-positive, on ART*

$$\frac{dX_{g,a,r}^{8,6,h,s,x,p}\left( t \right)}{dt}= \sum_{d=3}^{7} \sum_{v=1}^{5} \left( A_{g,a}^{d}\left( t \right)\cdot X_{g,a,r}^{d,v,h,s,x,p}\left( t \right)-\sigma_{g,a,r}^{d,v}{\cdot X}_{g,a,r}^{8,6,h,s,x,p}\left( t \right) \right)$$

### HPV

| **Equation variables** | |
| --- | --- |
| ${\mu\_HPV}_{g}^{d,h,s,x}$ | Annual cervical cancer-associated mortality rate by gender *g*, HIV disease stage *d*, vaccine-type HPV stage *h,* non-vaccine-type HPV stage *s,* and cervical cancer stage *x* for (1 ≤ *x* ≤ 3).  Only women have cervical cancer-associated mortality (${\mu\_HPV}_{1}^{d,h,s,x}$ = 0) and only when (*h* = 6 or *s* = 6). |
| ${\lambda\_vHPV}_{g,a,r}(t)$ | Force of vaccine-type HPV infection for susceptible persons of gender *g,* age *a,* and risk *r*. |
| ${\lambda\_nvHPV}_{g,a,r}(t)$ | Force of non-vaccine-type HPV infection for susceptible persons of gender *g,* age *a,* and risk *r*. |
| $ж_{d}$ | HPV acquisition risk multiplier for individuals with HIV with CD4 count (4 ≤ *d* ≤ 7)*.* |
| ${\psi\_HPV}_{g}$ | HPV acquisition reduction multiplier due to population-level condom use by gender. |
| $\xi_{g,a}$ | HPV acquisition reduction multiplier by gender and age for individuals with type-specific natural immunity.  Only women temporarily develop partial natural immunity ($\xi_{1,a}=0$). Older women develop stronger natural immunity than young girls. |
| $\phi_{a}$ | Vaccine-type HPV acquisition reduction multiplier by age for vaccinated individuals.  We assume life-long protection with vaccination ($\phi_{a}$ is equivalent for all vaccinated ages). |
| ${k\_v}_{g,a}^{h,h^{'}}$ | Transition rate of progressing or regressing from vaccine-type HPV precancer or disease stage *h* to stage *h’*.  Only women develop precancerous lesions and cervical cancer (${k\_v}_{1,a}^{h,h^{'}}$= 0 except for HPV clearance when *h* = 2 and *h’* = 1). |
| ${k\_nv}_{g,a}^{s,s^{'}}$ | Transition rate of progressing or regressing from non-vaccine-type HPV precancer or disease stage *s* to stage *s’*.  Only women develop precancerous lesions and cervical cancer (${k\_v}_{1,a}^{s,s^{'}}$= 0 except for HPV clearance when *s* = 2 and s*’* = 1). |
| $r_{g}$ | Rate of waning type-specific natural immunity.  Only women temporarily develop partial natural immunity ($r_{1}=0$). |
| $\varphi_{g}^{h,s,x,x^{'}}$ | Progression rate of cervical cancer from stage *x* to stage *x’*.  Only women develop cervical cancer ($\varphi_{1}^{h,s,x,x^{'}}=0$) and ($\varphi_{2}^{h,s,x,x^{'}}>0$ only when *h* or *s* = 6) |
| ${\zeta\_v}^{d,h,h^{'}}$ | Transition rate multiplier for individuals with HIV progressing or regressing from vaccine-type precancer or disease stage *h* to stage *h’* with CD4 count *d.*  Transition rate multipliers for individuals with HIV are the same for vaccine-type and non-vaccine-type HPV (${\zeta\_v}^{d,h,h^{'}}= {\zeta\_nv}^{d,s,s^{'}}$ when *h* = *s* and *h’* = *s’*)*.* |
| ${\zeta\_nv}^{d,s,s^{'}}$ | Transition rate multiplier for individuals with HIV progressing or regressing from non-vaccine-type precancer or disease stage *s* to stage *s’* with gender *g* and CD4 count *d.*  Transition rate multipliers for individuals with HIV are the same for vaccine-type and non-vaccine-type HPV (${\zeta\_v}^{d,h,h^{'}}= {\zeta\_nv}^{d,s,s^{'}}$ when *h* = *s* and *h’* = *s’*)*.* |
| $\mathcal{l}_{g}$ | Additional multiplier for clearance of vaccine or non-vaccine-type HPV infection.  Only applied to men ($\mathcal{l}_{2}=1$). |
| $V_{g,a}^{d}$ | The proportion of persons with HIV disease status *d,* gender *g,* and age *a* vaccinated*.* |

We calculate changes in HPV status and precancer or disease stage for women without hysterectomy (1 ≤ *x* ≤ 3). We track vaccine-type and non-vaccine type HPV independently, but only enumerate cancer incidence for the first infection to progress to local cervical cancer. CIN1,2,3 can regress and HPV infection can clear naturally. Women who clear HPV temporarily develop partial natural immunity against reinfection with the same HPV type group while men who clear HPV do not develop natural immunity. Persons susceptible to type-specific HPV infection or with temporary natural immunity can acquire HPV after sexual debut. Individuals with HIV experience higher rates of HPV acquisition and disease progression, and lower rates of HPV clearance, immunity waning, and disease regression. Cervical cancer mortality varies by cancer stage and HIV status, and affects individuals regardless of type-specific etiology. We assume that the nonavalent HPV vaccine provides lifelong protection against vaccine-type HPV and no protection against non-vaccine-type HPV. We assume the vaccine is ineffective for persons with current vaccine-type HPV infection, and the equations therefore only reflect vaccination of persons susceptible or immune to vaccine-type HPV. Vaccination does not depend on non-vaccine-type HPV infection status. Although not shown in the equations below, persons are screened according to their age *a* and lose their screened status upon aging out of the screened age group.

### Vaccine-type HPV and precancer equations

*Men, susceptible*

$$\frac{dX_{1,a,r}^{d,v,1,s,1,1}\left( t \right)}{dt}= {\mathcal{l}_{1}\cdot\zeta\_v}^{d,2,1}\cdot{k\_v}_{1,a}^{2,1}\cdot X_{1,a,r}^{d,v,2,s,1,1}\left( t \right) -\left( ж_{d}\cdot{\psi\_HPV}_{1}{\cdot\lambda\_vHPV}_{1,a,r}(t)+V_{1,a}^{d} \right)X_{1,a,r}^{d,v,1,s,1,1}\left( t \right)$$

*Men, HPV-infected*

$$\frac{dX_{1,a,r}^{d,v,2,s,1,1}\left( t \right)}{dt}={ж_{d}\cdot{\psi\_HPV}_{1}{\cdot\lambda\_vHPV}_{1,a,r}(t)\cdot X}_{1,a,r}^{d,v,1,s,1,1}\left( t \right)$$

$$- {\mathcal{l}_{1}\cdot\zeta\_v}^{d,2,1}\cdot{k\_v}_{1,a}^{2,1}\cdot X_{1,a,r}^{d,v,2,s,1,1}\left( t \right)$$

*Men, susceptible, vaccinated*

$$\frac{dX_{1,a,r}^{d,v,1,s,1,2}\left( t \right)}{dt}= V_{1,a}^{d}\cdot X_{1,a,r}^{d,v,1,s,1,1}\left( t \right)+ {\mathcal{l}_{1}\cdot\zeta\_v}^{d,2,1}\cdot{k\_v}_{1,a}^{2,1}\cdot X_{1,a,r}^{d,v,2,s,1,2}\left( t \right) -{\phi_{a}\cdotж_{d}\cdot{\psi\_HPV}_{1}{\cdot\lambda\_vHPV}_{1,a,r}(t)\cdot X}_{1,a,r}^{d,v,1,s,1,2}\left( t \right)$$

*Men, HPV-infected, vaccinated*

$$\frac{dX_{1,a,r}^{d,v,2,s,1,2}\left( t \right)}{dt}={{\phi_{a}\cdotж}_{d}\cdot{\psi\_HPV}_{1}{\cdot\lambda\_vHPV}_{1,a,r}(t)\cdot X}_{1,a,r}^{d,v,1,s,1,2}\left( t \right)$$

$$- {\mathcal{l}_{1}\cdot\zeta\_v}^{d,2,1}\cdot{k\_v}_{1,a}^{2,1}\cdot X_{1,a,r}^{d,v,2,s,1,2}\left( t \right)$$

*Women, susceptible*

$$\frac{dX_{2,a,r}^{d,v,1,s,x,[1,3]}\left( t \right)}{dt}= {\zeta\_v}^{d,7,1}\cdot r_{2}\cdot X_{2,a,r}^{d,v,7,s,x,[1,3]}\left( t \right) -\left( ж_{d}\cdot{\psi\_HPV}_{2}{\cdot\lambda\_vHPV}_{2,a,r}(t)+V_{2,a}^{d}+{\mu\_HPV}_{2}^{d,1,s,x} \right)X_{2,a,r}^{d,v,1,s,x,[1,3]}\left( t \right)$$

*Women, immune*

$$\frac{dX_{2,a,r}^{d,v,7,s,x,[1,3]}\left( t \right)}{dt}= V_{2,a}^{d}\cdot X_{2,a,r}^{d,v,7,s,x,[1,3]}\left( t \right)+{\zeta\_v}^{d,2,7}\cdot{k\_v}_{2,a}^{2,7}\cdot X_{2,a,r}^{d,v,2,s,x,[1,3]}\left( t \right) -\left( {\zeta\_v}^{d,7,1}\cdot{k\_v}_{2,a}^{7,1}+\xi_{2,a}{\cdotж}_{d}\cdot{\psi\_HPV}_{2}{\cdot\lambda\_vHPV}_{2,a,r}(t)+V_{2,a}^{d}+{\mu\_HPV}_{2}^{d,7,s,x} \right)X_{2,a,r}^{d,v,7,s,x,[1,3]}\left( t \right)$$

*Women, HPV-infected*

$$\frac{dX_{2,a,r}^{d,v,2,s,x,[1,3]}\left( t \right)}{dt}= {\zeta\_v}^{d,3,2}\cdot{k\_v}_{2,a}^{3,2}\cdot X_{2,a,r}^{d,v,3,s,x,\left[ 1,3 \right]}\left( t \right) +ж_{d}\cdot{\psi\_HPV}_{2}{\cdot\lambda\_vHPV}_{2,a,r}\left( t \right)\cdot X_{2,a,r}^{d,v,1,s,x,\left[ 1,3 \right]}\left( t \right)+\xi_{2,a}{\cdotж}_{d}\cdot{\psi\_HPV}_{2}{\cdot\lambda\_vHPV}_{2,a,r}\left( t \right)\cdot X_{2,a,r}^{d,v,7,s,x,\left[ 1,3 \right]}\left( t \right)-\left( {\zeta\_v}^{d,2,7}\cdot{k\_v}_{2,a}^{2,7}+{\zeta\_v}^{d,2,3}\cdot{k\_v}_{2,a}^{2,3}+{\mu\_HPV}_{2}^{d,2,s,x} \right)X_{2,a,r}^{d,v,2,s,x,[1,3]}\left( t \right)$$

*Women, susceptible, vaccinated*

$$\frac{dX_{2,a,r}^{d,v,1,s,x,[2,4]}\left( t \right)}{dt}= {\zeta\_v}^{d,7,1}\cdot r_{2}\cdot X_{2,a,r}^{d,v,7,s,x,\left[ 2,4 \right]}\left( t \right)+V_{2,a}^{d}\cdot X_{2,a,r}^{d,v,1,s,x,[1,3]}\left( t \right)-\left( \phi_{a}\cdotж_{d}\cdot{\psi\_HPV}_{2}{\cdot\lambda\_vHPV}_{2,a,r}(t)+{\mu\_HPV}_{2}^{d,1,s,x} \right)X_{2,a,r}^{d,v,1,s,x,[2,4]}\left( t \right)$$

*Women, immune, vaccinated*

$$\frac{dX_{2,a,r}^{d,v,7,s,x,[2,4]}\left( t \right)}{dt}= {\zeta\_v}^{d,2,7}\cdot{k\_v}_{2,a}^{2,7}\cdot X_{2,a,r}^{d,v,2,s,x,\left[ 2,4 \right]}\left( t \right)+V_{2,a}^{d}\cdot X_{2,a,r}^{d,v,7,s,x,[1,3]}\left( t \right) -\left( {\zeta\_v}^{d,7,1}\cdot{k\_v}_{2,a}^{7,1}+{\phi_{a}\cdot\xi}_{2,a}{\cdotж}_{d}\cdot{\psi\_HPV}_{2}{\cdot\lambda\_vHPV}_{2,a,r}(t)+{\mu\_HPV}_{2}^{d,7,s,x} \right)X_{2,a,r}^{d,v,7,s,x,[2,4]}\left( t \right)$$

*Women, HPV-infected, vaccinated*

$$\frac{dX_{2,a,r}^{d,v,2,s,x,[2,4]}\left( t \right)}{dt}= {\zeta\_v}^{d,3,2}\cdot{k\_v}_{2,a}^{3,2}\cdot X_{2,a,r}^{d,v,3,s,x,\left[ 2,4 \right]}\left( t \right) +{\phi_{a}\cdotж}_{d}\cdot{\psi\_HPV}_{2}{\cdot\lambda\_vHPV}_{2,a,r}\left( t \right)\cdot X_{2,a,r}^{d,v,2,s,x,\left[ 2,4 \right]}\left( t \right)+{\phi_{a}\cdot\xi}_{2,a}{\cdotж}_{d}\cdot{\psi\_HPV}_{2}{\cdot\lambda\_vHPV}_{2,a,r}\left( t \right)\cdot X_{2,a,r}^{d,v,7,s,x,\left[ 2,4 \right]}\left( t \right)-\left( {\zeta\_v}^{d,2,7}\cdot{k\_v}_{2,a}^{2,7}+{\zeta\_v}^{d,2,3}\cdot{k\_v}_{2,a}^{2,3}+{\mu\_HPV}_{2}^{d,2,s,x} \right)X_{2,a,r}^{d,v,2,s,x,[2,4]}\left( t \right)$$

*Women, CIN1*

$$\frac{dX_{2,a,r}^{d,v,3,s,x,p}\left( t \right)}{dt}= {\zeta\_v}^{d,4,3}\cdot{k\_v}_{2,a}^{4,3}\cdot X_{2,a,r}^{d,v,4,s,x,p}\left( t \right) +{\zeta\_v}^{d,2,3}\cdot{k\_v}_{2,a}^{2,3}\cdot X_{2,a,r}^{d,v,2,s,x,p}\left( t \right)-\left( {\zeta\_v}^{d,3,4}\cdot{k\_v}_{2,a}^{3,4}+{\zeta\_v}^{d,3,2}\cdot{k\_v}_{2,a}^{3,2}+{\mu\_HPV}_{2}^{d,3,s,x} \right)X_{2,a,r}^{d,v,3,s,x,p}\left( t \right)$$

*Women, CIN2*

$$\frac{dX_{2,a,r}^{d,v,4,s,x,p}\left( t \right)}{dt}= {\zeta\_v}^{d,5,4}\cdot{k\_v}_{2,a}^{5,4}\cdot X_{2,a,r}^{d,v,5,s,x,p}\left( t \right) +{\zeta\_v}^{d,3,4}\cdot{k\_v}_{2,a}^{3,4}\cdot X_{2,a,r}^{d,v,3,s,x,p}\left( t \right)-\left( {\zeta\_v}^{d,4,5}\cdot{k\_v}_{2,a}^{4,5}+{\zeta\_v}^{d,4,3}\cdot{k\_v}_{2,a}^{4,3}+{\mu\_HPV}_{2}^{d,4,s,x} \right)X_{2,a,r}^{d,v,4,s,x,p}\left( t \right)$$

*Women, CIN3*

$$\frac{dX_{2,a,r}^{d,v,5,s,x,p}\left( t \right)}{dt}= {\zeta\_v}^{d,4,5}\cdot{k\_v}_{2,a}^{4,5}\cdot X_{2,a,r}^{d,v,4,s,x,p}\left( t \right)-\left( {\zeta\_v}^{d,5,6}\cdot{k\_v}_{2,a}^{5,6}+{\zeta\_v}^{d,5,4}\cdot{k\_v}_{2,a}^{5,4}+{\mu\_HPV}_{2}^{d,5,s,x} \right)X_{2,a,r}^{d,v,5,s,x,p}\left( t \right)$$

### Non-vaccine-type HPV and precancer equations

The non-vaccine-type HPV and precancer equations follow the same pattern as the vaccine-type HPV equations with a few updates. All values of *s* equal the values of *h* in the vaccine-type equations, and *h* equals any value. The appropriate force of infection, transition rates, and transition rate multipliers for individuals living with HIV should be used. Vaccination does not depend on non-vaccine-type HPV infection status.

### Cervical cancer equations

*Women, cervical cancer, local*

*(where h=6)*

$$\frac{dX_{2,a,r}^{d,v,6,s,x,p}\left( t \right)}{dt}= {\zeta\_v}^{d,5,6}\cdot{k\_v}_{2,a}^{5,6}\cdot X_{2,a,r}^{d,v,5,s,x,p}\left( t \right)$$

*(where s=6)*

$$\frac{dX_{2,a,r}^{d,v,h,6,x,p}\left( t \right)}{dt}= {\zeta\_v}^{d,5,6}\cdot{k\_v}_{2,a}^{5,6}\cdot X_{2,a,r}^{d,v,h,6,x,p}\left( t \right)$$

*(where h=6 or s=6)*

$$\frac{dX_{2,a,r}^{d,v,h,s,1,p}\left( t \right)}{dt}=-\left( \varphi_{2}^{h,s,1,2}+{\mu\_HPV}_{2}^{d,h,s,1} \right)X_{2,a,r}^{d,v,h,s,1,p}\left( t \right)$$

*Women, cervical cancer, regional (where h=6 or s=6)*

$$\frac{dX_{2,a,r}^{d,v,h,s,2,p}\left( t \right)}{dt}= \varphi_{2}^{h,s,1,2}\cdot X_{2,a,r}^{d,v,h,s,1,p}\left( t \right)-\left( \varphi_{2}^{h,s,2,3}+{\mu\_HPV}_{2}^{d,h,s,2} \right)X_{2,a,r}^{d,v,h,s,2,p}\left( t \right)$$

*Women, cervical cancer, distant (where h=6 or s=6)*

$$\frac{dX_{2,a,r}^{d,v,h,s,3,p}\left( t \right)}{dt}= \varphi_{2}^{h,s,2,3}\cdot X_{2,a,r}^{d,v,h,s,2,p}\left( t \right)-\left( {\mu\_HPV}_{2}^{d,h,s,3} \right)X_{2,a,r}^{d,v,h,s,3,p}\left( t \right)$$

# V. Additional Model Output

## V.a. Sensitivity analyses

### Isolating the impact of differences in screening test performance

Results from scenarios defined by 90% HPV vaccination coverage and single-visit strategies with the same frequency, coverage, and high loss to follow-up for treatment as in the baseline scenario (36% of screen-positive individuals treated) are presented in Figure S24 and Table S33. If high loss to follow-up continues, changing to the single-visit strategies will have limited impact on cervical cancer incidence or the timeline to elimination relative to the scenario with baseline screening and 90% vaccination. As shown in Figure S24 in the dashed lines, the reduction in cervical cancer incidence with single-visit strategies comes from the expected decrease in loss to follow-up that these strategies are expected to facilitate. There are, however, some differences in the efficiency of screening due to differences in specificity, with the number needed to treat to avert one cancer case ranging from 1.0 with HPV genotyping to 8.8 with AVE under scenarios with high loss to follow-up and 2 to 28 under scenarios with lower loss to follow-up.


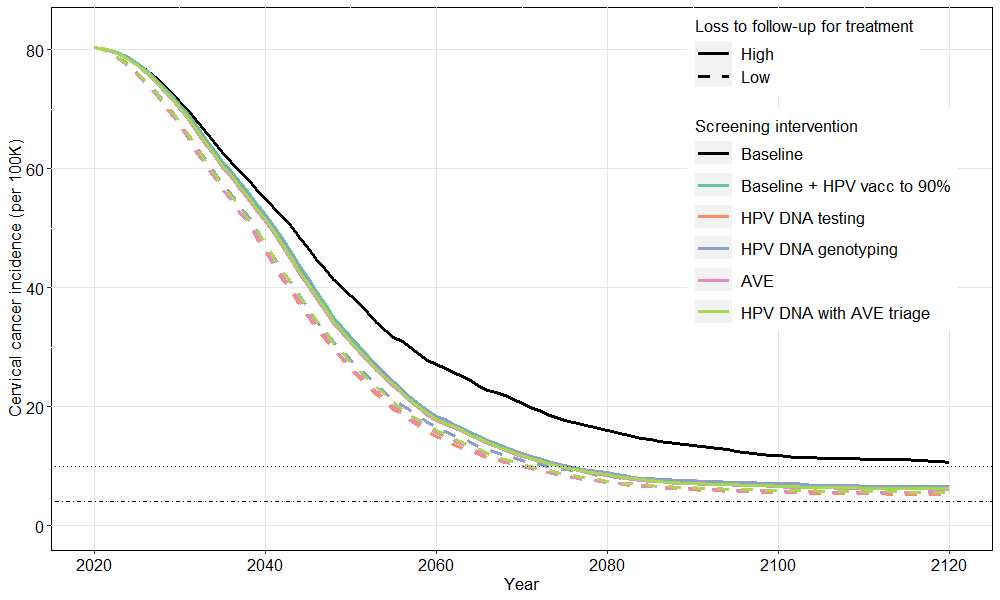


**Figure S24. Model-estimated age-standardized cervical cancer incidence under scenarios with one-time screening using a range of interventions with variable sensitivity, specificity, and loss to follow-up.** Plotted lines show the median estimated cancer incidence rates over time from the 25 simulations, standardized to the 2015 World Population. The solid lines show the results from scenarios with screening at 48% coverage in ages 35-39 and high loss to follow-up, with 36% of screen-positive individuals receiving treatment. HPV vaccine coverage is 57% in the baseline scenario and increases to 90% in all other scenarios. The dashed lines show the projected cancer incidence with the single-visit strategies (HPV DNA testing, HPV DNA genotyping, automated visual evaluation [AVE], and HPV DNA testing with AVE triage) if retention for treatment were increased to 95% for thermocoagulation and 80% for LLETZ.

### Moderate loss to follow-up

The high retention for treatment with single-visit strategies assumed in the main model scenarios may not be achieved; some programs have observed lower retention, particularly if clients do not wait at the clinic for their HPV results. We compared model predictions from the main model scenario using repeat HIV-targeted HPV DNA testing and low loss to follow-up (the most effective modeled strategy) with those from a scenario increasing loss to follow-up to more moderate levels of 30% for thermocoagulation (70% retention) and 50% for LLETZ (Figure S25, Table S33). With this moderate loss to follow-up scenario, an estimated 46.4% of cancer cases are expected to be averted by 2120 relative to baseline (range 42.2%, 52.5%). For comparison, 54.3% (48.7%, 60.2%) are expected to be averted in the scenario with low loss to follow-up (95% retention for thermocoagulation and 80% for LLETZ). In the moderate loss to follow-up scenario, median age-standardized incidence reaches 4.2 per 100,000 (2.1, 9.8) in 2120, just above the elimination threshold.

### Screening coverage increased to 70%

The main model scenarios assume that 48% of individuals in each targeted age group are screened. To evaluate the impact of meeting the World Health Organization target of 70% screening coverage by 2030,^144^ we increased the proportion of women screened in each age group to 70% using a strategy of repeat HIV-targeted HPV DNA testing (Figure S25, Table S33). Compared to the expected 54.3% (48.7%, 60.2%) of cancer cases averted relative to baseline by 2120 under this same strategy with 48% coverage, 63.0% (54.9%, 67.9%) of cancer cases are expected to be averted with 70% coverage. Higher coverage also increases the speed and likelihood of reaching elimination, moving from a median predicted elimination date of 2095 to 2072. However, even with this higher coverage, median incidence does not cross below the elimination threshold for women with HIV (Table S33).


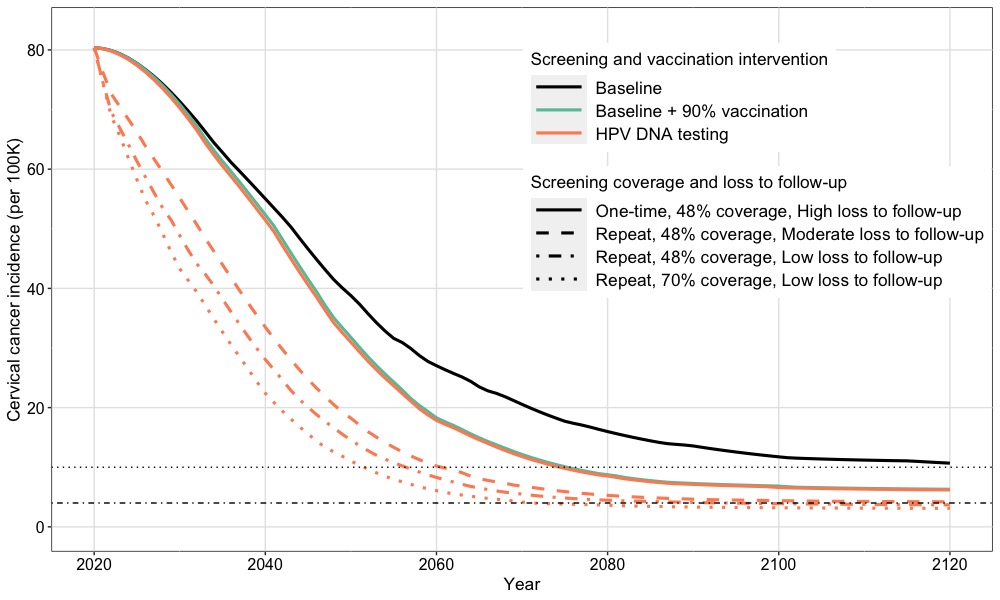


**Figure S25. Model-estimated age-standardized cervical cancer incidence under scenarios varying in screening intervention, coverage, and loss to follow-up for treatment.** Plotted lines show the median estimated cancer incidence rates over time from the 25 simulations, standardized to the 2015 World Population. The solid lines show the results from scenarios with once-lifetime screening at 48% coverage in ages 35-39 and high loss to follow-up (36% of screen-positive individuals receiving treatment). HPV vaccine coverage is 57% in the baseline scenario and increases to 90% in all other scenarios. The dashed and dotted lines show the results with varying assumptions regarding loss to follow-up for treatment and coverage using repeat HIV-targeted screening with HPV DNA testing. In the HIV-targeted scenario, women without HIV are screened at ages 35-39 and 45-49, and women with HIV are screened every 5 years from 25-49. Low loss to follow-up assumes 95% retention for thermocoagulation and 80% for LLETZ. Moderate loss to follow-up reduces these to 70% for thermocoagulation and 50% for LLETZ.

### Variable AVE screening and diagnostic performance

Automated visual evaluation (AVE) is a new technology that is still under development and has not yet been rigorously evaluated. As such, there is substantial uncertainty regarding the performance of the AVE, particularly for women with HIV.^110^ We defined scenarios varying the sensitivity and specificity of AVE and exploring differential performance by HIV status (Table S23). Figure S26, below, presents the median projected incidence from simulations using these performance inputs in scenarios with repeat HIV-targeted screening. For comparison, we show the median estimates from HPV DNA testing alone and HPV DNA genotyping. Incidence rates in 2120 with range from 4.0 to 4.2 per 100,000 with the varying inputs for performance of AVE as primary screening and 4.2 to 4.3 per 100,000 for AVE as triage following HPV DNA testing (Table S33).

With the base inputs for AVE performance (98% sensitivity for CIN2+ and specificity ranging from 78% for untreated women with HIV to 84% for women without HIV; Table S22), 53% (48%, 60%) of cervical cancer cases are averted over the 100-year simulation with AVE as primary screening. With both specificity and sensitivity reduced by 20% for all groups, this drops modestly to 48% (44%, 56%). The number needed to treat to avert one cervical cancer case relative to baseline increases from 30.9 (15.5, 51.3) to 58.7 (27.4, 100.0). In the base strategy using AVE as triage with HPV DNA testing, 50% (46%, 57%) of cases are averted, compared to 46% (42%, 53%) with 20% reductions in sensitivity and specificity. The number needed to treat increases from 7.2 (4.7, 11.9) to 9.1 (5.9, 16.9).


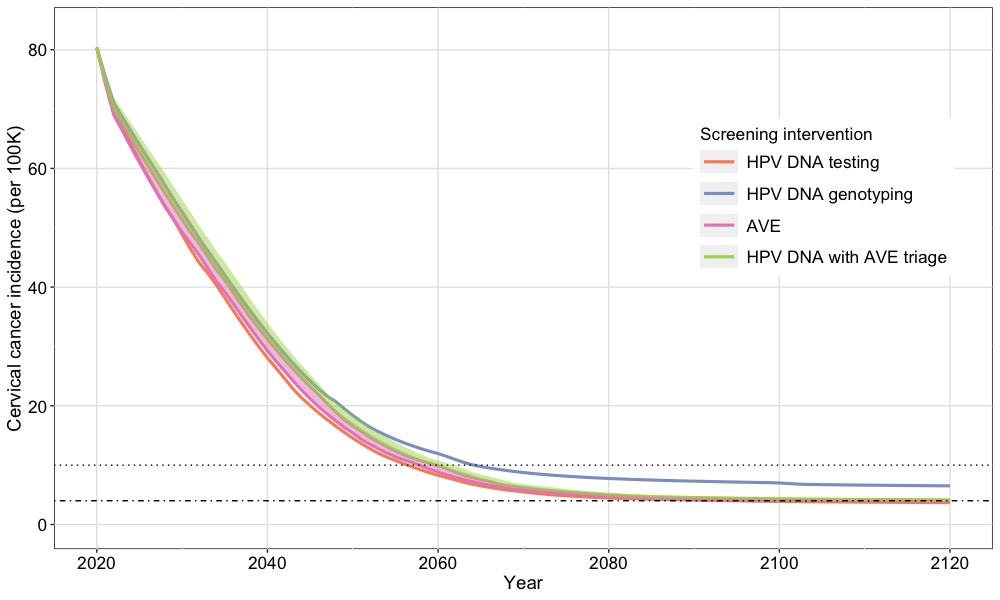


**Figure S26. Model-estimated age-standardized cervical cancer incidence under scenarios with single-visit strategies and varying performance of automated visual evaluation (AVE).** Plotted lines show the median estimated cancer incidence rates over time from the 25 simulations, standardized to the 2015 World Population. All scenarios presented assume 90% 9v HPV vaccine coverage, repeat screening at ages 35-39 and 45-49 for women without HIV and every 5 years from 25-49 years for women with HIV, with 48% of women screened in each targeted age group, and low loss to follow-up (95% retention for thermocoagulation and 80% for LLETZ). The results using the base assumptions for AVE performance are shown in solid lines, and the shaded pink and green areas show the range of the median estimates from scenarios varying the performance of AVE as primary screening and as triage following HPV diagnosis, respectively.

| **Table S33. Projected age-standardized cervical cancer incidence and time to elimination under sensitivity analysis scenarios for all women, women living without HIV, and women living with HIV** | | | | | | |
| --- | --- | --- | --- | --- | --- | --- |
|  | Age-standardized^a^ cervical cancer incidence (per 100K)  *Median (range)^b^* | | | Year elimination threshold reached^c^  *Median (range)^b^* | | |
|  | Year 2030 (after 10 years) | Year 2070 (after 50 years) | Year 2120 (after 100 years) | | <10/100K | <4/100K |
| **All women** |  |  |  | |  |  |
| *Three-visit screening and treatment between ages 35-39 (baseline, for comparison)* |  |  |  | |  |  |
| Baseline (cytology with colposcopy triage) | 71.3 (50.4, 106.4) | 20.5 (10.3, 39.3) | 10.7 (4.2, 29.9) | | X (2071, X) | X (X, X) |
| Baseline with vaccine scale-up^d^ | 70.9 (50.1, 105.8) | 12.1 (6.2, 21.0) | 6.3 (3.3, 13.6) | | 2076 (2060, X) | X (2096, X) |
| *Single-visit interventions between ages 35-39, same treatment retention as baseline* |  |  |  | |  |  |
| HPV DNA testing | 70.3 (49.7, 104.5) | 11.8 (6.1, 20.3) | 6.2 (3.2, 13.3) | | 2075 (2059, X) | X (2093, X) |
| HPV DNA genotyping | 70.5 (49.8, 105.0) | 12.1 (6.3, 21.2) | 6.5 (3.4, 14.1) | | 2076 (2060, X) | X (2100, X) |
| AVE | 70.1 (49.6, 104.2) | 11.7 (6.0, 20.1) | 6.1 (3.2, 13.2) | | 2075 (2059, X) | X (2092, X) |
| HPV DNA with AVE triage | 70.3 (49.7, 104.6) | 11.8 (6.1, 20.3) | 6.2 (3.2, 13.3) | | 2075 (2059, X) | X (2093, X) |
| *HPV DNA testing with HIV-targeted screening^e^* |  |  |  | |  |  |
| Screening coverage to 70% | 43.3 (31.6, 62.7) | 4.2 (2.3, 8.3) | 3.1 (1.3, 7.1) | | 2052 (2044, 2063) | 2072 (2055, X) |
| Moderate treatment retention^f^ | 55.2 (39.1, 77.9) | 6.8 (3.6, 12.8) | 4.2 (2.1, 9.8) | | 2061 (2050, 2102) | X (2067, X) |
| *AVE with HIV-targeted screening^e^, variable performance* |  |  |  | |  |  |
| Relative 10% lower performance for untreated women with HIV | 50.1 (36.4, 70.8) | 5.9 (3.2, 11.2) | 4.0 (2.1, 9.0) | | 2059 (2048, 2078) | X (2063, X) |
| Relative 10% lower performance for all | 51.1 (36.8, 71.9) | 6.1 (3.3, 11.6) | 4.1 (2.1, 9.2) | | 2060 (2048, 2081) | X (2094, X) |
| Relative 20% lower performance for untreated women with HIV, 10% lower for HIV-negative women | 52.1 (37.4, 73.1) | 6.2 (3.3, 11.7) | 4.1 (2.1, 9.2) | | 2060 (2049, 2083) | X (2064, X) |
| Relative 20% lower performance for all | 53.1 (37.9, 74.3) | 6.4 (3.5, 12.1) | 4.2 (2.1, 9.5) | | 2061 (2049, 2088) | X (2065, X) |
| *HPV DNA with AVE triage, HIV-targeted screening^e^, variable performance of AVE* |  |  |  | |  |  |
| Relative 10% lower performance for untreated women with HIV | 52.3 (37.6, 73.4) | 6.3 (3.4, 12.0) | 4.2 (2.2, 9.4) | | 2061 (2049, 2087) | X (2065, X) |
| Relative 10% lower performance for all | 53.2 (38.0, 74.4) | 6.5 (3.5, 12.3) | 4.2 (2.2, 9.6) | | 2062 (2049, 2093) | X (2066, X) |
| Relative 20% lower performance for untreated women with HIV, 10% lower for HIV-negative women | 54.0 (38.6, 75.5) | 6.6 (3.6, 12.5) | 4.3 (2.2, 9.7) | | 2062 (2050, 2097) | X (2066, X) |
| Relative 20% lower performance for all | 54.8 (39.0, 76.7) | 6.8 (3.7, 12.9) | 4.3 (2.2, 9.9) | | 2062 (2050, 2109) | X (2067, X) |
| **Women without HIV** |  |  |  | |  |  |
| *Three-visit screening and treatment between ages 35-39 (baseline, for comparison)* |  |  |  | |  |  |
| Baseline (cytology with colposcopy triage) | 32.8 (15.7, 51.3) | 10.9 (4.5, 23.8) | 6.3 (2.2, 18.9) | | 2074 (2046, X) | X (2075, X) |
| Baseline with vaccine scale-up^d^ | 32.4 (15.5, 50.9) | 6.1 (2.7, 12.3) | 4.1 (1.7, 9.3) | | 2058 (2042, 2085) | X (2059, X) |
| *Single-visit interventions between ages 35-39, same treatment retention as baseline* |  |  |  | |  |  |
| HPV DNA testing | 32.2 (15.4, 50.4) | 6.0 (2.6, 12.0) | 4.1 (1.7, 9.2) | | 2058 (2042, 2083) | X (2059, X) |
| HPV DNA genotyping | 32.3 (15.5, 50.6) | 6.1 (2.8, 12.5) | 4.3 (1.8, 9.6) | | 2058 (2042, 2093) | X (2059, X) |
| AVE | 32.1 (15.4, 50.2) | 5.9 (2.6, 11.9) | 4.1 (1.7, 9.1) | | 2058 (2042 2082) | X (2058, X) |
| HPV DNA with AVE triage | 32.2 (15.4, 50.4) | 6.0 (2.7, 12.0) | 4.1 (1.7, 9.2) | | 2058 (2042, 2083) | X (2059, X) |
| *HPV DNA testing with HIV-targeted screening^e^* |  |  |  | |  |  |
| Screening coverage to 70% | 25.6 (12.1, 38.5) | 2.9 (1.4, 6.5) | 2.4 (1.0, 5.9) | | 2046 (2034, 2057) | 2062 (2047, X) |
| Moderate treatment retention^f^ | 29.0 (13.8, 44.4) | 4.3 (2.0, 9.1) | 3.2 (1.3, 7.5) | | 2053 (2038, 2067) | 2074 (2053, X) |
| *AVE with HIV-targeted screening^e^, variable performance* |  |  |  | |  |  |
| Relative 10% lower performance for untreated women with HIV | 27.4 (12.9, 40.8) | 4.0 (1.8, 8.2) | 3.1 (1.3, 7.0) | | 2051 (2036, 2063) | 2071 (2050, X) |
| Relative 10% lower performance for all | 27.9 (13.2, 41.8) | 4.1 (1.8, 8.4) | 3.1 (1.3, 7.2) | | 2051 (2037, 2064) | 2072 (2051, X) |
| Relative 20% lower performance for untreated women with HIV, 10% lower for HIV-negative women | 27.9 (13.2, 41.8) | 4.1 (1.8, 8.4) | 3.1 (1.3, 7.2) | | 2051 (2037, 2064) | 2072 (2051, X) |
| Relative 20% lower performance for all | 28.3 (13.4, 42.7) | 4.3 (1.9, 8.7) | 3.2 (1.3, 7.3) | | 2052 (2037, 2065) | 2073 (2052, X) |
| *HPV DNA with AVE triage, HIV-targeted screening^e^, variable performance of AVE* |  |  |  | |  |  |
| Relative 10% lower performance for untreated women with HIV | 28.3 (13.3, 42.4) | 4.3 (1.9, 8.7) | 3.2 (1.3, 7.3) | | 2052 (2037, 2065) | 2073 (2052, X) |
| Relative 10% lower performance for all | 28.6 (13.5, 43.2) | 4.4 (2.0, 8.9) | 3.2 (1.4, 7.5) | | 2052 (2037, 2066) | 2074 (2052, X) |
| Relative 20% lower performance for untreated women with HIV, 10% lower for HIV-negative women | 28.6 (13.5, 43.2) | 4.4 (2.0, 9.0) | 3.2 (1.4, 7.5) | | 2052 (2037, 2066) | 2074 (2052, X) |
| Relative 20% lower performance for all | 29.0 (13.7, 44.1) | 4.5 (2.0, 9.2) | 3.2 (1.4, 7.6) | | 2053 (2038, 2067) | 2076 (2053, X) |
| **Women with HIV** |  |  |  | |  |  |
| *Three-visit screening and treatment between ages 35-39 (baseline, for comparison)* |  |  |  | |  |  |
| Baseline (cytology with colposcopy triage) | 158.1 (116.0, 244.8) | 48.1 (24.6, 98.5) | 24.3 (10.5, 74.6) | | X (X, X) | X (X, X) |
| Baseline with vaccine scale-up^d^ | 157.1 (115.5, 243.8) | 29.3 (15.2, 57.1) | 14.9 (8.2, 35.4) | | X (2092, X) | X (X, X) |
| *Single-visit interventions between ages 35-39, same treatment retention as baseline* |  |  |  | |  |  |
| HPV DNA testing | 155.9 (114.6, 241.2) | 28.6 (14.7, 55.0) | 14.6 (8.0, 34.6) | | X (2089, X) | X (X, X) |
| HPV DNA genotyping | 156.3 (114.9, 242.1) | 29.3 (15.3, 57.4) | 15.3 (8.5, 36.7) | | X (2095, X) | X (X, X) |
| AVE | 155.7 (114.5, 240.7) | 28.4 (14.6, 54.5) | 14.6 (8.0, 34.4) | | X (2089, X) | X (X, X) |
| HPV DNA with AVE triage | 155.9 (114.7, 241.4) | 28.6 (14.7, 55.1) | 14.6 (8.0, 34.6) | | X (2089, X) | X (X, X) |
| *HPV DNA testing with HIV-targeted screening^e^* |  |  |  | |  |  |
| Screening coverage to 70% | 96.6 (70.2, 140.2) | 7.7 (4.6, 16.4) | 6.1 (2.2, 14.1) | | 2063 (2052, X) | X (2083, X) |
| Moderate treatment retention^f^ | 120.8 (89.4, 175.4) | 14.4 (7.9, 29.0) | 8.5 (4.9, 21.6) | | 2084 (2063, X) | X (X, X) |
| *AVE with HIV-targeted screening^e^, variable performance* |  |  |  | |  |  |
| Relative 10% lower performance for untreated women with HIV | 111.6 (81.3, 160.0) | 12.5 (6.9, 24.8) | 8.3 (4.4, 19.7) | | 2078 (2059, X) | X (X, X) |
| Relative 10% lower performance for all | 113.4 (82.5, 162.4) | 12.7 (7.0, 25.5) | 8.3 (4.6, 20.0) | | 2079 (2060, X) | X (X, X) |
| Relative 20% lower performance for untreated women with HIV, 10% lower for HIV-negative women | 115.6 (84.5, 165.8) | 13.0 (7.2, 26.3) | 8.4 (4.8, 20.5) | | 2081 (2061, X) | X (X, X) |
| Relative 20% lower performance for all | 117.1 (85.7, 168.2) | 13.3 (7.4, 27.1) | 8.5 (4.9, 20.9) | | 2082 (2061, X) | X (X, X) |
| *HPV DNA with AVE triage, HIV-targeted screening^e^, variable performance of AVE* |  |  |  | |  |  |
| Relative 10% lower performance for untreated women with HIV | 115.8 (84.4, 165.7) | 13.2 (7.3, 26.7) | 8.6 (4.9, 20.7) | | 2083 (2061, X) | X (X, X) |
| Relative 10% lower performance for all | 117.1 (85.5, 168.0) | 13.7 (7.5, 27.4) | 8.7 (5.0, 21.1) | | 2085 (2062, X) | X (X, X) |
| Relative 20% lower performance for untreated women with HIV, 10% lower for HIV-negative women | 119.0 (87.3, 171.2) | 14.2 (7.7, 28.3) | 8.9 (5.1, 21.9) | | 2087 (2062, X) | X (X, X) |
| Relative 20% lower performance for all | 120.4 (88.5, 173.5) | 14.9 (7.9, 29.1) | 9.0 (5.1, 21.5) | | 2088 (2063, X) | X (X, X) |
| ^a^Standardized to the 2015 World Population; ^b^Median and range of estimates from simulations using the 25 best-fitting parameter sets; ^c^X denotes that the elimination threshold was not reached in the simulated time horizon; ^d^Nonavalent hrHPV vaccination of girls aged 9-14 scaled up from 57% to 90% coverage. Vaccination coverage remains at 90% for all single-visit scenarios; ^e^Ages 35-39 and 45-49 for women living without HIV and every 5 years from 25 to 49 for women living with HIV. ^f^70% retention for thermalcoagulation, 50% for large loop excision of the transformation zone (LLETZ). | | | | | | |

## V.b. Cervical cancer incidence under additional counterfactual scenarios

Under the baseline scenario, our model projects that cervical cancer incidence will decline 87% over the coming century. To identify the factors contributing to this decline, we defined four counterfactual scenarios: 1) the baseline scenario without HPV vaccination, 2) the baseline scenario without HPV vaccination or cervical cancer screening, 3) the baseline scenario without HPV vaccination, cervical cancer screening, or VMMC, and 4) the baseline scenario without HPV vaccination, cervical cancer screening, VMMC, or ART.

The plot below shows the age-standardized cervical cancer incidence under these scenarios from 1980 (before the generalized HIV epidemic) to 2020. The black line shows the baseline scenario as defined for the main analysis, which includes 57% coverage with pre-adolescent nonavalent HPV vaccination, low levels of cervical cancer screening and treatment, VMMC, condoms, and ART (see sections [II.e.i](#ART), [II.e.ii.](#condoms), [II.e.iii](#circumcision), [II.e.iv](#historical_HPV), and [II.f.](#Scenarios)). In a scenario with all these interventions except HPV vaccination (the green line below), incidence is projected to decline to a lesser extent, reaching an age-standardized rate of 45.6 per 100,000 women in 2120 compared to the rate of 10.7 per 100,000 projected in the baseline scenario. The yellow line shows that incidence would be very slightly higher without baseline screening. Additionally removing VMMC would increase incidence historically and into the future, reaching a rate of 63.6 per 100,000 in 2120 (purple line). Lastly, the orange line shows estimated incidence in a scenario without ART. Because ART has effects on diverse model dynamics, including HIV transmission, HPV acquisition and progression, mortality, and fertility, the impact of removing this intervention is not as straightforward. The model estimates that cervical cancer incidence would not rise as high initially, but that incidence would be higher in the long term. Together, these scenarios indicate that the projected decrease in cancer incidence in the baseline scenario reflects the combined effects of HPV vaccination, VMMC, ART, and, to a lesser extent, screening.


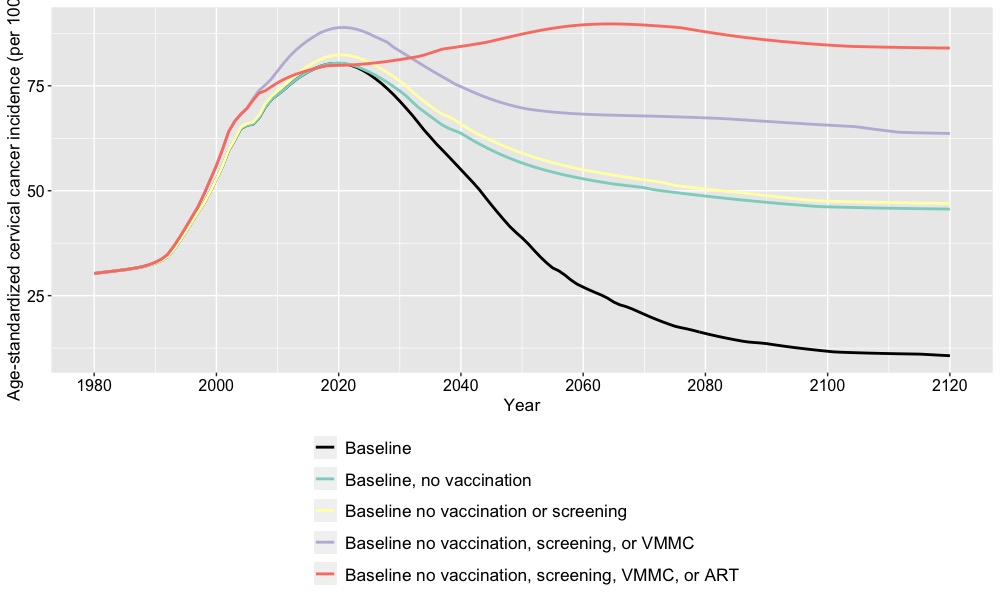


**Figure S27. Model-estimated age-standardized cervical cancer incidence under the baseline and counterfactual scenarios.** Plotted lines show the median estimated cancer incidence rates over time from the 25 simulations, standardized to the 2015 World Population.

## V.c. Projected HIV prevalence

All modeled scenarios assume continued use of ART, VMMC, and condoms, which contribute to reductions in HIV prevalence. Figure S28 shows the median and range of estimates for HIV prevalence over time among women aged 15 and older in the baseline scenario. Prevalence is estimated to be 35% in 2021 and to decline to 22% in 2120.


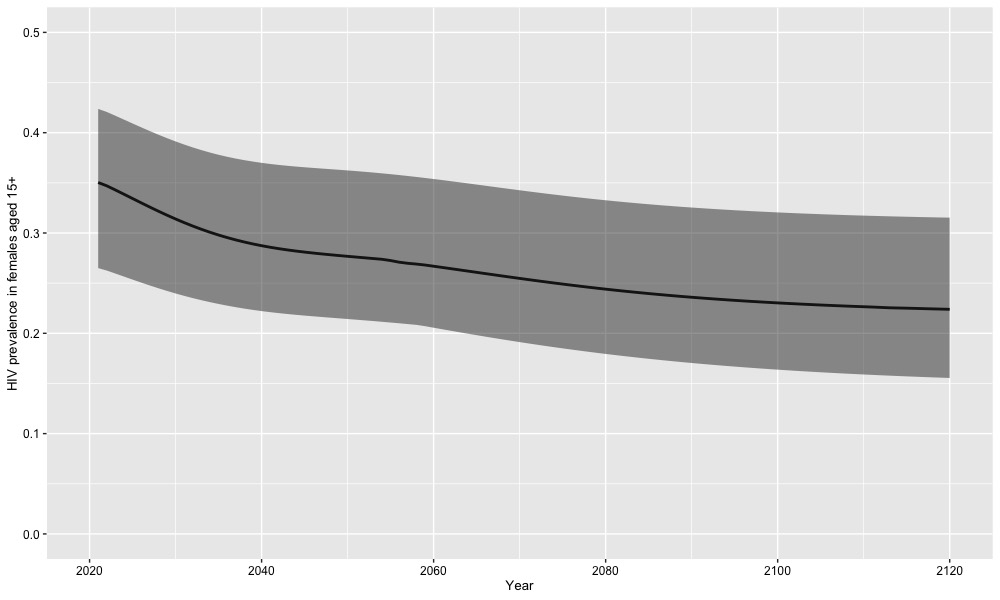


**Figure S28. Model-estimated HIV prevalence among women aged 15 and older from 2021 to 2120 under the baseline scenario.** The dark line shows the median HIV prevalence, and the shaded region shows the range of estimates from the 25 simulations.

# VI. References

1. National Department of Health (NDoH) SSASS, South African Medical Research, Council (SAMRC) I. South Africa Demographic and Health Survey 2016. Pretoria, South Africa, and Rockville, Maryland, USA, 2019.

2. Simbayi LC ZK, Zungu N, Moyo S, Marinda E, Jooste S, Mabaso M, Ramlagan S, North A, van Zyl J, Mohlabane N, Dietrich C, Naidoo I, SABSSM V Team. The Fifth South African National HIV Prevalence, Incidence, Behaviour and Communications Survey, 2017. Cape Town, 2019.

3. Tan N, Sharma M, Winer R, Galloway D, Rees H, Barnabas RV. Model-estimated effectiveness of single dose 9-valent HPV vaccination for HIV-positive and HIV-negative females in South Africa. *Vaccine* 2018; **36**(32 Pt A): 4830-6.

4. Department of Economic and Social Affairs PD. World Population Prospects 2019. In: Nations U, editor. Rev. 1 ed. Online Edition; 2019.

5. Africa SS. Mid-year population estimates 2019. Statistical Release P0302. Pretoria, South Africa; 2019.

6. Moultrie TA, Hosegood V, McGrath N, Hill C, Herbst K, Newell ML. Refining the criteria for stalled fertility declines: an application to rural KwaZulu-Natal, South Africa, 1990-2005. *Stud Fam Plann* 2008; **39**(1): 39-48.

7. Moultrie TA, Timaeus IM. The South African fertility decline: Evidence from two censuses and a Demographic and Health Survey. *Popul Stud (Camb)* 2003; **57**(3): 265-83.

8. Anderson RM, May RM, Ng TW, Rowley JT. Age-dependent choice of sexual partners and the transmission dynamics of HIV in Sub-Saharan Africa. *Philos Trans R Soc Lond B Biol Sci* 1992; **336**(1277): 135-55.

9. Ross A, Van der Paal L, Lubega R, Mayanja BN, Shafer LA, Whitworth J. HIV-1 disease progression and fertility: the incidence of recognized pregnancy and pregnancy outcome in Uganda. *AIDS* 2004; **18**(5): 799-804.

10. Bobat R, Coovadia H, Coutsoudis A, Moodley D. Determinants of mother-to-child transmission of human immunodeficiency virus type 1 infection in a cohort from Durban, South Africa. *Pediatr Infect Dis J* 1996; **15**(7): 604-10.

11. Horwood C, Vermaak K, Butler L, Haskins L, Phakathi S, Rollins N. Elimination of paediatric HIV in KwaZulu-Natal, South Africa: large-scale assessment of interventions for the prevention of mother-to-child transmission. *Bull World Health Organ* 2012; **90**(3): 168-75.

12. Rollins N, Little K, Mzolo S, Horwood C, Newell ML. Surveillance of mother-to-child transmission prevention programmes at immunization clinics: the case for universal screening. *AIDS* 2007; **21**(10): 1341-7.

13. Network GBoDC. Global Burden of Disease Study 2017 (GBD 2017) Results. In: (IHME) IfHMaE, editor. Seattle, United States; 2018.

14. Institute AHR. Africa Centre cohort data from KwaZulu-Natal, SA Surveillance data repository. Durban, South Africa.

15. Rao DW, Wheatley MM, Goodreau SM, Enns EA. Partnership dynamics in mathematical models and implications for representation of sexually transmitted infections: a review. *Ann Epidemiol* 2021; **59**: 72-80.

16. Garnett GP, Gregson S. Monitoring the course of the HIV-1 epidemic: The influence of patterns of fertility on HIV-1 prevalence estimates. *Mathematical Population Studies* 2000; **8**: 251-77.

17. Ott MQ, Barnighausen T, Tanser F, Lurie MN, Newell ML. Age-gaps in sexual partnerships: seeing beyond 'sugar daddies'. *AIDS* 2011; **25**(6): 861-3.

18. de Oliveira T, Kharsany AB, Graf T, et al. Transmission networks and risk of HIV infection in KwaZulu-Natal, South Africa: a community-wide phylogenetic study. *Lancet HIV* 2017; **4**(1): e41-e50.

19. Hubert JB, Burgard M, Dussaix E, et al. Natural history of serum HIV-1 RNA levels in 330 patients with a known date of infection. The SEROCO Study Group. *AIDS* 2000; **14**(2): 123-31.

20. Lodi S, Phillips A, Touloumi G, et al. Time from human immunodeficiency virus seroconversion to reaching CD4+ cell count thresholds <200, <350, and <500 Cells/mm(3): assessment of need following changes in treatment guidelines. *Clin Infect Dis* 2011; **53**(8): 817-25.

21. Lyles RH, Munoz A, Yamashita TE, et al. Natural history of human immunodeficiency virus type 1 viremia after seroconversion and proximal to AIDS in a large cohort of homosexual men. Multicenter AIDS Cohort Study. *J Infect Dis* 2000; **181**(3): 872-80.

22. Pantazis N, Morrison C, Amornkul PN, et al. Differences in HIV natural history among African and non-African seroconverters in Europe and seroconverters in sub-Saharan Africa. *PLoS One* 2012; **7**(3): e32369.

23. Badri M, Lawn SD, Wood R. Short-term risk of AIDS or death in people infected with HIV-1 before antiretroviral therapy in South Africa: a longitudinal study. *Lancet* 2006; **368**(9543): 1254-9.

24. Lewden C, Gabillard D, Minga A, et al. CD4-specific mortality rates among HIV-infected adults with high CD4 counts and no antiretroviral treatment in West Africa. *J Acquir Immune Defic Syndr* 2012; **59**(2): 213-9.

25. Maduna PH, Dolan M, Kondlo L, et al. Morbidity and mortality according to latest CD4+ cell count among HIV positive individuals in South Africa who enrolled in project Phidisa. *PLoS One* 2015; **10**(4): e0121843.

26. Adler WH, Baskar PV, Chrest FJ, Dorsey-Cooper B, Winchurch RA, Nagel JE. HIV infection and aging: mechanisms to explain the accelerated rate of progression in the older patient. *Mech Ageing Dev* 1997; **96**(1-3): 137-55.

27. Newell ML, Coovadia H, Cortina-Borja M, et al. Mortality of infected and uninfected infants born to HIV-infected mothers in Africa: a pooled analysis. *Lancet* 2004; **364**(9441): 1236-43.

28. Beachler DC, Jenkins G, Safaeian M, Kreimer AR, Wentzensen N. Natural Acquired Immunity Against Subsequent Genital Human Papillomavirus Infection: A Systematic Review and Meta-analysis. *J Infect Dis* 2016; **213**(9): 1444-54.

29. Kong X, Wang MC, Gray R. Analysis of longitudinal multivariate outcome data from couples cohort studies: application to HPV transmission dynamics. *J Am Stat Assoc* 2015; **110**(510): 472-85.

30. Liu G, Sharma M, Tan N, Barnabas RV. HIV-positive women have higher risk of human papilloma virus infection, precancerous lesions, and cervical cancer. *AIDS* 2018; **32**(6): 795-808.

31. Sankaranarayanan R, Swaminathan R, Brenner H, et al. Cancer survival in Africa, Asia, and Central America: a population-based study. *Lancet Oncol* 2010; **11**(2): 165-73.

32. Dryden-Peterson S, Bvochora-Nsingo M, Suneja G, et al. HIV Infection and Survival Among Women With Cervical Cancer. *J Clin Oncol* 2016; **34**(31): 3749-57.

33. Campos NG, Burger EA, Sy S, et al. An updated natural history model of cervical cancer: derivation of model parameters. *Am J Epidemiol* 2014; **180**(5): 545-55.

34. Bruni L AG, Serrano B, Mena M, Gómez D, Muñoz J, Bosch FX, de Sanjosé S. Human Papillomavirus and Related Diseases in South Africa: Summary Report 17 June 2019.

35. de Sanjose S, Quint WG, Alemany L, et al. Human papillomavirus genotype attribution in invasive cervical cancer: a retrospective cross-sectional worldwide study. *Lancet Oncol* 2010; **11**(11): 1048-56.

36. Denny L, Adewole I, Anorlu R, et al. Human papillomavirus prevalence and type distribution in invasive cervical cancer in sub-Saharan Africa. *Int J Cancer* 2014; **134**(6): 1389-98.

37. van Aardt MC, Dreyer G, Pienaar HF, et al. Unique human papillomavirus-type distribution in South African women with invasive cervical cancer and the effect of human immunodeficiency virus infection. *Int J Gynecol Cancer* 2015; **25**(5): 919-25.

38. Dartell M, Rasch V, Kahesa C, et al. Human papillomavirus prevalence and type distribution in 3603 HIV-positive and HIV-negative women in the general population of Tanzania: the PROTECT study. *Sex Transm Dis* 2012; **39**(3): 201-8.

39. Van Aardt MC, Dreyer G, Snyman LC, Richter KL, Becker P, Mojaki SM. Oncogenic and incidental HPV types associated with histologically confirmed cervical intraepithelial neoplasia in HIV-positive and HIV-negative South African women. *S Afr Med J* 2016; **106**(6).

40. Clifford GM, Rana RK, Franceschi S, Smith JS, Gough G, Pimenta JM. Human papillomavirus genotype distribution in low-grade cervical lesions: comparison by geographic region and with cervical cancer. *Cancer Epidemiol Biomarkers Prev* 2005; **14**(5): 1157-64.

41. Van Aardt MC, Dreyer G, Richter KL, Becker P. Human papillomavirus-type distribution in South African women without cytological abnormalities: a peri-urban study. *Southern African Journal of Gynaecological Oncology* 2013; **5**(sup1): S21-S7.

42. Johnson HC, Elfstrom KM, Edmunds WJ. Inference of type-specific HPV transmissibility, progression and clearance rates: a mathematical modelling approach. *PLoS One* 2012; **7**(11): e49614.

43. Quinn TC, Wawer MJ, Sewankambo N, et al. Viral load and heterosexual transmission of human immunodeficiency virus type 1. Rakai Project Study Group. *N Engl J Med* 2000; **342**(13): 921-9.

44. Lingappa JR, Hughes JP, Wang RS, et al. Estimating the impact of plasma HIV-1 RNA reductions on heterosexual HIV-1 transmission risk. *PLoS One* 2010; **5**(9): e12598.

45. Hollingsworth TD, Anderson RM, Fraser C. HIV-1 transmission, by stage of infection. *J Infect Dis* 2008; **198**(5): 687-93.

46. Boily MC, Baggaley RF, Wang L, et al. Heterosexual risk of HIV-1 infection per sexual act: systematic review and meta-analysis of observational studies. *Lancet Infect Dis* 2009; **9**(2): 118-29.

47. Rodger AJ, Cambiano V, Bruun T, et al. Sexual Activity Without Condoms and Risk of HIV Transmission in Serodifferent Couples When the HIV-Positive Partner Is Using Suppressive Antiretroviral Therapy. *JAMA* 2016; **316**(2): 171-81.

48. Eisinger RW, Dieffenbach CW, Fauci AS. HIV Viral Load and Transmissibility of HIV Infection: Undetectable Equals Untransmittable. *JAMA* 2019; **321**(5): 451-2.

49. Brinkhof MW, Boulle A, Weigel R, et al. Mortality of HIV-infected patients starting antiretroviral therapy in sub-Saharan Africa: comparison with HIV-unrelated mortality. *PLoS Med* 2009; **6**(4): e1000066.

50. Cornell M, Johnson LF, Wood R, et al. Twelve-year mortality in adults initiating antiretroviral therapy in South Africa. *J Int AIDS Soc* 2017; **20**(1): 21902.

51. de Coninck Z, Hussain-Alkhateeb L, Bratt G, et al. Non-AIDS Mortality Is Higher Among Successfully Treated People Living with HIV Compared with Matched HIV-Negative Control Persons: A 15-Year Follow-Up Cohort Study in Sweden. *AIDS Patient Care STDS* 2018; **32**(8): 297-305.

52. Lilian RR, Rees K, Mabitsi M, McIntyre JA, Struthers HE, Peters RPH. Baseline CD4 and mortality trends in the South African human immunodeficiency virus programme: Analysis of routine data. *South Afr J HIV Med* 2019; **20**(1): 963.

53. Meyer-Rath G, Johnson LF, Pillay Y, et al. Changing the South African national antiretroviral therapy guidelines: The role of cost modelling. *PLoS One* 2017; **12**(10): e0186557.

54. UNAIDS. 2020. <https://www.unaids.org/en/regionscountries/countries/southafrica>.

55. Kharsany ABM, Cawood C, Lewis L, et al. Trends in HIV Prevention, Treatment, and Incidence in a Hyperendemic Area of KwaZulu-Natal, South Africa. *JAMA Netw Open* 2019; **2**(11): e1914378.

56. Takuva S, Brown AE, Pillay Y, Delpech V, Puren AJ. The continuum of HIV care in South Africa: implications for achieving the second and third UNAIDS 90-90-90 targets. *AIDS* 2017; **31**(4): 545-52.

57. Zaidi J, Grapsa E, Tanser F, Newell ML, Barnighausen T. Dramatic increase in HIV prevalence after scale-up of antiretroviral treatment. *AIDS* 2013; **27**(14): 2301-5.

58. Weller S, Davis K. Condom effectiveness in reducing heterosexual HIV transmission. *Cochrane Database Syst Rev* 2002; (1): CD003255.

59. Pierce Campbell CM, Lin HY, Fulp W, et al. Consistent condom use reduces the genital human papillomavirus burden among high-risk men: the HPV infection in men study. *J Infect Dis* 2013; **208**(3): 373-84.

60. Winer RL, Hughes JP, Feng Q, et al. Condom use and the risk of genital human papillomavirus infection in young women. *N Engl J Med* 2006; **354**(25): 2645-54.

61. Gray RH, Kigozi G, Serwadda D, et al. Male circumcision for HIV prevention in men in Rakai, Uganda: a randomised trial. *Lancet* 2007; **369**(9562): 657-66.

62. Weiss HA, Quigley MA, Hayes RJ. Male circumcision and risk of HIV infection in sub-Saharan Africa: a systematic review and meta-analysis. *AIDS* 2000; **14**(15): 2361-70.

63. Tobian AA, Kacker S, Quinn TC. Male circumcision: a globally relevant but under-utilized method for the prevention of HIV and other sexually transmitted infections. *Annu Rev Med* 2014; **65**: 293-306.

64. Gray RH, Serwadda D, Kong X, et al. Male circumcision decreases acquisition and increases clearance of high-risk human papillomavirus in HIV-negative men: a randomized trial in Rakai, Uganda. *J Infect Dis* 2010; **201**(10): 1455-62.

65. Albero G, Castellsague X, Lin HY, et al. Male circumcision and the incidence and clearance of genital human papillomavirus (HPV) infection in men: the HPV Infection in men (HIM) cohort study. *BMC Infect Dis* 2014; **14**: 75.

66. Albero G, Villa LL, Lazcano-Ponce E, et al. Male circumcision and prevalence of genital human papillomavirus infection in men: a multinational study. *BMC Infect Dis* 2013; **13**: 18.

67. Vanbuskirk K, Winer RL, Hughes JP, et al. Circumcision and acquisition of human papillomavirus infection in young men. *Sex Transm Dis* 2011; **38**(11): 1074-81.

68. Vardas E, Giuliano AR, Goldstone S, et al. External genital human papillomavirus prevalence and associated factors among heterosexual men on 5 continents. *J Infect Dis* 2011; **203**(1): 58-65.

69. Connolly C, Simbayi LC, Shanmugam R, Nqeketo A. Male circumcision and its relationship to HIV infection in South Africa: results of a national survey in 2002. *S Afr Med J* 2008; **98**(10): 789-94.

70. Doyle D. Ritual male circumcision: a brief history. *J R Coll Physicians Edinb* 2005; **35**(3): 279-85.

71. HIV/AIDS WHOaJUNPo. Male circumcision: global trends and determinants of prevalence, safety and acceptability. Switzerland; 2007.

72. Shisana O RT, Simbayi LC, Zuma K, Jooste S, Zungu N, Labadarios D,, Onoya D ea. South African National HIV Prevalence, Incidence and Behaviour Survey, 2012. Cape Town, 2014.

73. Shisana O SL, Rehle T, Zungu NP, Zuma K, Ngogo N, Jooste S, PillayVan Wyk V, Parker W, Pezi S, Davids A, Nwanyanwu O, Dinh TH, SABSSM III Implementation, Team. South African National HIV Prevalence, Incidence, Behaviour and Communication Survey, 2008: The health of our children. Cape Town, 2010.

74. WHO/UNICEF. Update on global and national programme coverage April 2020, 2020.

75. de Sanjose S, Serrano B, Tous S, et al. Burden of Human Papillomavirus (HPV)-Related Cancers Attributable to HPVs 6/11/16/18/31/33/45/52 and 58. *JNCI Cancer Spectr* 2018; **2**(4): pky045.

76. Kreimer AR, Sampson JN, Porras C, et al. Evaluation of Durability of a Single Dose of the Bivalent HPV Vaccine: The CVT Trial. *J Natl Cancer Inst* 2020; **112**(10): 1038-46.

77. Denny L. Prevention of cervical cancer. *Reprod Health Matters* 2008; **16**(32): 18-31.

78. Godfrey MAL, Mathenjwa S, Mayat N. Rural Zulu women's knowledge of and attitudes towards Pap smears and adherence to cervical screening. *Afr J Prim Health Care Fam Med* 2019; **11**(1): e1-e6.

79. Gakidou E, Nordhagen S, Obermeyer Z. Coverage of cervical cancer screening in 57 countries: low average levels and large inequalities. *PLoS Med* 2008; **5**(6): e132.

80. Jordaan S, P M, K R, Simoens C, Bogers J-P. A Review of Cervical Cancer in South Africa: Previous, Current and Future. *Health Care : Current Reviews* 2016; **04**.

81. Africa NDoHRoS. Cervical Cancer Prevention and Control Policy, 2017.

82. Arbyn M, Sankaranarayanan R, Muwonge R, et al. Pooled analysis of the accuracy of five cervical cancer screening tests assessed in eleven studies in Africa and India. *Int J Cancer* 2008; **123**(1): 153-60.

83. Khozaim K, Orang'o E, Christoffersen-Deb A, et al. Successes and challenges of establishing a cervical cancer screening and treatment program in western Kenya. *Int J Gynaecol Obstet* 2014; **124**(1): 12-8.

84. Msyamboza KP, Phiri T, Sichali W, Kwenda W, Kachale F. Cervical cancer screening uptake and challenges in Malawi from 2011 to 2015: retrospective cohort study. *BMC Public Health* 2016; **16**(1): 806.

85. Ouedraogo Y, Furlane G, Fruhauf T, et al. Expanding the Single-Visit Approach for Cervical Cancer Prevention: Successes and Lessons From Burkina Faso. *Glob Health Sci Pract* 2018; **6**(2): 288-97.

86. Catarino R, Petignat P, Dongui G, Vassilakos P. Cervical cancer screening in developing countries at a crossroad: Emerging technologies and policy choices. *World J Clin Oncol* 2015; **6**(6): 281-90.

87. Sherris J, Wittet S, Kleine A, et al. Evidence-based, alternative cervical cancer screening approaches in low-resource settings. *Int Perspect Sex Reprod Health* 2009; **35**(3): 147-54.

88. Debeaudrap P, Sobngwi J, Tebeu PM, Clifford GM. Residual or recurrent precancerous lesions after treatment of cervical lesions in HIV-infected women: a systematic review and meta-analysis of treatment failure. *Clin Infect Dis* 2019.

89. Hoffman SR, Le T, Lockhart A, et al. Patterns of persistent HPV infection after treatment for cervical intraepithelial neoplasia (CIN): A systematic review. *Int J Cancer* 2017; **141**(1): 8-23.

90. National Department of Health RoSA. National Guideline for Cervical Cancer Control and Management. 2019.

91. Jordaan S, Michelow P, Simoens C, Bogers J. Challenges and Progress of Policies on Cervical Cancer in South Africa. *Health Care : Current Reviews* 2017; **05**(01).

92. National Department of H, Statistics South A, South African Medical Research C, Icf. South Africa Demographic and Health Survey 2016. Pretoria, South Africa and Rockville, Maryland, USA: NDoH, Stats SA, SAMRC, and ICF, 2019.

93. World Health O. WHO estimates of human papillomavirus immunization coverage, 2019 update. World Health Organization; 2019.

94. Whitworth HS, Gallagher KE, Howard N, et al. Efficacy and immunogenicity of a single dose of human papillomavirus vaccine compared to no vaccination or standard three and two-dose vaccination regimens: A systematic review of evidence from clinical trials. *Vaccine* 2020; **38**(6): 1302-14.

95. Johnson LG, Saidu R, Mbulawa Z, et al. Selecting human papillomavirus genotypes to optimize the performance of screening tests among South African women. *Cancer Med* 2020; **9**(18): 6813-24.

96. van Schalkwyk C, Moodley J, Welte A, Johnson LF. Modelling the impact of prevention strategies on cervical cancer incidence in South Africa. *International Journal of Cancer* 2021.

97. Maiman M, Tarricone N, Vieira J, Suarez J, Serur E, Boyce JG. Colposcopic evaluation of human immunodeficiency virus-seropositive women. *Obstet Gynecol* 1991; **78**(1): 84-8.

98. Spinillo A, Capuzzo E, Tenti P, De Santolo A, Piazzi G, Iasci A. Adequacy of screening cervical cytology among human immunodeficiency virus-seropositive women. *Gynecol Oncol* 1998; **69**(2): 109-13.

99. Anderson JR, Paramsothy P, Heilig C, et al. Accuracy of Papanicolaou Test among HIV-Infected Women. *Clinical Infectious Diseases* 2006; **42**(4): 562-8.

100. Boardman LA, Peipert JF, Cooper AS, Cu-Uvin S, Flanigan T, Raphael SI. Cytologic-histologic discrepancy in human immunodeficiency virus-positive women referred to a colposcopy clinic. *Obstet Gynecol* 1994; **84**(6): 1016-20.

101. Firnhaber C, Mayisela N, Mao L, et al. Validation of cervical cancer screening methods in HIV positive women from Johannesburg South Africa. *PloS One* 2013; **8**(1): e53494.

102. Arbyn M, Bergeron C, Klinkhamer P, Martin-Hirsch P, Siebers AG, Bulten J. Liquid compared with conventional cervical cytology: a systematic review and meta-analysis. *Obstet Gynecol* 2008; **111**(1): 167-77.

103. Republic of South Africa National Department of H. Cervical Cancer Prevention and Control Policy. 2017.

104. Kelly H, Mayaud P, Segondy M, Pant Pai N, Peeling RW. A systematic review and meta-analysis of studies evaluating the performance of point-of-care tests for human papillomavirus screening. *Sex Transm Infect* 2017; **93**(S4): S36-S45.

105. Saidu R, Kuhn L, Tergas A, et al. Performance of Xpert HPV on Self-collected Vaginal Samples for Cervical Cancer Screening Among Women in South Africa. *J Low Genit Tract Dis* 2020; **25**(1): 15-21.

106. Castle PE, Ajeh R, Dzudie A, et al. A comparison of screening tests for detection of high-grade cervical abnormalities in women living with HIV from Cameroon. *Infect Agent Cancer* 2020; **15**: 45.

107. Chung MH, McKenzie KP, De Vuyst H, et al. Comparing Papanicolau smear, visual inspection with acetic acid and human papillomavirus cervical cancer screening methods among HIV-positive women by immune status and antiretroviral therapy. *AIDS (London, England)* 2013; **27**(18): 2909-19.

108. Magdi R, Elshafeey F, Elshebiny M, et al. A systematic review and meta-analysis of diagnostic accuracy of HPV tests for the screening of cervical cancer in low-resource settings. *International Journal of Gynaecology and Obstetrics: The Official Organ of the International Federation of Gynaecology and Obstetrics* 2020.

109. Hu L, Bell D, Antani S, et al. An Observational Study of Deep Learning and Automated Evaluation of Cervical Images for Cancer Screening. *J Natl Cancer Inst* 2019; **111**(9): 923-32.

110. Desai KT, Befano B, Xue Z, et al. The development of "automated visual evaluation" for cervical cancer screening: The promise and challenges in adapting deep-learning for clinical testing: Interdisciplinary principles of automated visual evaluation in cervical screening. *Int J Cancer* 2022; **150**(5): 741-52.

111. Katanga J, Kjaer SK, Manongi R, et al. Performance of careHPV, hybrid capture 2 and visual inspection with acetic acid for detection of high-grade cervical lesion in Tanzania: A cross-sectional study. *PLoS ONE* 2019; **14**(6).

112. Mapanga W, Girdler-Brown B, Feresu SA, Chipato T, Singh E. Prevention of cervical cancer in HIV-seropositive women from developing countries through cervical cancer screening: a systematic review. *Syst Rev* 2018; **7**(1): 198.

113. Moscicki A-B, Durako SJ, Ma Y, Darragh T, Vermund SH. Utility of cervicography in HIV-infected and uninfected adolescents. *Journal of Adolescent Health* 2003; **32**(3): 204-13.

114. de Fouw M, Oosting RM, Rutgrink A, Dekkers OM, Peters AAW, Beltman JJ. A systematic review and meta-analysis of thermal coagulation compared with cryotherapy to treat precancerous cervical lesions in low- and middle-income countries. *International Journal of Gynaecology and Obstetrics: The Official Organ of the International Federation of Gynaecology and Obstetrics* 2019; **147**(1): 4-18.

115. Pinder LF, Parham GP, Basu P, et al. Thermal ablation versus cryotherapy or loop excision to treat women positive for cervical precancer on visual inspection with acetic acid test: pilot phase of a randomised controlled trial. *Lancet Oncol* 2020; **21**(1): 175-84.

116. Blumenthal PD, Gaffikin L, Deganus S, et al. Cervical cancer prevention: safety, acceptability, and feasibility of a single-visit approach in Accra, Ghana. *Am J Obstet Gynecol* 2007; **196**(4): 407.e1-8; discussion .e8-9.

117. Cubie HA, Campbell C. Cervical cancer screening – The challenges of complete pathways of care in low-income countries: Focus on Malawi. *Womens Health (Lond)* 2020; **16**.

118. Shiferaw N, Salvador-Davila G, Kassahun K, et al. The Single-Visit Approach as a Cervical Cancer Prevention Strategy Among Women With HIV in Ethiopia: Successes and Lessons Learned. *Global Health, Science and Practice* 2016; **4**(1): 87-98.

119. World Health O, International Agency for Research on C, African P, Health Research C. Prevention of cervical cancer through screening using visual inspection with acetic acid (VIA) and treatment with cryotherapy. A demonstration project in six African countries: Malawi, Madagascar, Nigeria, Uganda, the United Republic of Tanzania, & Zambia. Geneva: WHO, 2012.

120. Anderson J, Wysong M, Estep D, et al. Evaluation of Cervical Cancer Screening Programs in Côte d’Ivoire, Guyana, and Tanzania: Effect of HIV Status. *PLoS ONE* 2015; **10**(9).

121. Lewis KDC, Sellors JW, Dawa A, Tsu VD, Kidula NA. Report on a cryotherapy service for women with cervical intraepithelial neoplasia in a district hospital in western Kenya. *Afr Health Sci* 2011; **11**(3): 370-6.

122. Oga EA, Brown JP, Brown C, et al. Recurrence of cervical intraepithelial lesions after thermo-coagulation in HIV-positive and HIV-negative Nigerian women. *BMC Womens Health* 2016; **16**.

123. Pfaff C, Singano V, Akello H, et al. Early experiences in integrating cervical cancer screening and treatment into HIV services in Zomba Central Hospital, Malawi. *Malawi Med J* 2018; **30**(3): 211-4.

124. Debeaudrap P, Sobngwi J, Tebeu P-M, Clifford GM. Residual or recurrent precancerous lesions after treatment of cervical lesions in HIV-infected women: a systematic review and meta-analysis of treatment failure. *Clin Infect Dis* 2019.

125. Hoffman SR, Le T, Lockhart A, et al. Patterns of persistent HPV infection after treatment for cervical intraepithelial neoplasia (CIN): A systematic review. *International Journal of Cancer* 2017; **141**(1): 8-23.

126. Khozaim K, Orang'o E, Christoffersen-Deb A, et al. Successes and challenges of establishing a cervical cancer screening and treatment program in western Kenya. *Int J Gynaecol Obstet* 2014; **124**(1): 12-8.

127. Forhan SE, Godfrey CC, Watts DH, Langley CL. A Systematic Review of the Effects of Visual Inspection With Acetic Acid, Cryotherapy, and Loop Electrosurgical Excision Procedures for Cervical Dysplasia in HIV-Infected Women in Low- and Middle-Income Countries. *JAIDS Journal of Acquired Immune Deficiency Syndromes* 2015; **68**: S350.

128. Filippi S, Barnes CP, Cornebise J, Stumpf MPH. On optimality of kernels for approximate Bayesian computation using sequential Monte Carlo. *Stat Appl Genet Mol* 2013; **12**(1).

129. Lenormand M, Jabot F, Deffuant G. Adaptive approximate Bayesian computation for complex models. *Computation Stat* 2013; **28**(6): 2777-96.

130. Toni T, Welch D, Strelkowa N, Ipsen A, Stumpf MPH. Approximate Bayesian computation scheme for parameter inference and model selection in dynamical systems. *J R Soc Interface* 2009; **6**(31): 187-202.

131. Beauclair R, Helleringer S, Hens N, Delva W. Age differences between sexual partners, behavioural and demographic correlates, and HIV infection on Likoma Island, Malawi. *Sci Rep* 2016; **6**: 36121.

132. Mabaso M, Mlangeni L, Makola L, et al. Factors associated with age-disparate sexual partnerships among males and females in South Africa: a multinomial analysis of the 2012 national population-based household survey data. *Emerg Themes Epidemiol* 2021; **18**(1): 3.

133. Africa SS. Census 2001: Post-enumeration survey: Results and methodology. Pretoria, South Africa; 2004.

134. Africa SS. Census 2011: Post-enumeration Survey: Results and methodology. Pretoria, South Africa; 2012.

135. Africa SS. Census 2001: Primary tables KwaZulu-Natal: 1996 and 2001 compared. Pretoria, South Africa; 2004.

136. Africa SS. Community Survey 2016. Statistical release P0301. Pretoria, South Africa; 2016.

137. Bray F, Ferlay J, Soerjomataram I, Siegel RL, Torre LA, Jemal A. Global cancer statistics 2018: GLOBOCAN estimates of incidence and mortality worldwide for 36 cancers in 185 countries. *CA: A Cancer Journal for Clinicians* 2018; **68**(6): 394-424.

138. Burchell AN, Richardson H, Mahmud SM, et al. Modeling the sexual transmissibility of human papillomavirus infection using stochastic computer simulation and empirical data from a cohort study of young women in Montreal, Canada. *Am J Epidemiol* 2006; **163**(6): 534-43.

139. Rosillon D, Baril L, Del Rosario-Raymundo MR, et al. Risk of newly detected infections and cervical abnormalities in adult women seropositive or seronegative for naturally acquired HPV-16/18 antibodies. *Cancer Med* 2019; **8**(10): 4938-53.

140. McDonald AC, Tergas AI, Kuhn L, Denny L, Wright TC, Jr. Distribution of Human Papillomavirus Genotypes among HIV-Positive and HIV-Negative Women in Cape Town, South Africa. *Front Oncol* 2014; **4**: 48.

141. Mbulawa ZZ, Coetzee D, Williamson AL. Human papillomavirus prevalence in South African women and men according to age and human immunodeficiency virus status. *BMC Infect Dis* 2015; **15**: 459.

142. Kuhn L, Saidu R, Boa R, et al. Clinical evaluation of modifications to a human papillomavirus assay to optimise its utility for cervical cancer screening in low-resource settings: a diagnostic accuracy study. *Lancet Glob Health* 2020; **8**(2): e296-e304.

143. Vandormael A, Akullian A, Siedner M, de Oliveira T, Barnighausen T, Tanser F. Declines in HIV incidence among men and women in a South African population-based cohort. *Nat Commun* 2019; **10**(1): 5482.

144. World Health Organization. Global strategy to accelerate the elimination of cervical cancer as a public health problem. Geneva: World Health Organization, 2020.
